# Evidence-Based Guidelines for the Diagnosis and Treatment of Pediatric CKD-Mineral and Bone Disorder (Version 2024)

**DOI:** 10.1016/j.ekir.2026.106492

**Published:** 2026-06-03

**Authors:** Mo Wang, Hua-Ying Xiong, Chunhua Zhu, Xinyi Yu, Jia Jiao, Liwen Tan, Ying Shen, Hong Xu, Jianhua Mao, Jianhua Zhou, Xiao-yun Jiang, Qian Shen, Fang Wang, Songming Huang, Wenyan Huang, Zheng-kun Xia, Xiao-rong Liu, Hui Wang, Yu-bin Wu, Li Yu, Yu-hong Tao, Chun-lin Gao, Ying-jie Li, Shu-Zhen Sun, Dong-feng Zhang, Xiao-shan Shao, Li-Jun Zhao, Xiaowen Wang, Xi-qiang Dang, Hui-mei Huang, Yaolong Chen, Qiu Li, Ai-Hua Zhang, Subspecialty Group of Nephrology, the Society of Pediatrics, Chinese Medical Association, Ai-Hua Zhang, Qui Li, Mo Wang, Hong Xu, Hui Wang, Chun-lin Gao, Xiao-rong Liu, Yu-bin Wu, Su-Zhen Sun, Yu-hong Tao, Xiao-shan Shao, Fang Wang, Li-Jun Zhao, Xiao-yun Jiang, Ying-jie Li, Li Yu, Zheng-kun Xia, Xi-qiang Dang, Jian-hua Mao, Jian-hua Zhou, Qian Shen, Wen-yan Huang, Xiao-wen Wang, Ying Shen, Song-ming Huang, Hui-mei Huang, Dong-feng Zhang

**Affiliations:** 1 Department of Nephrology, Children’s Hospital of Chongqing Medical University, Chongqing, China; 2 National Clinical Research Center for Children and Adolescents' Health and Diseases, Chongqing, China; 3 Department of Pediatric Nephrology, Children’s Hospital of Nanjing Medical University, Nanjing, China; 4 Department of Nephrology, Beijing Children’s Hospital, Capital Medical University, Beijing, China; 5 Department of Nephrology, Children’s Hospital of Fudan University, Shanghai, China; 6 Department of Nephrology, Children’s Hospital, Zhejiang University School of Medicine, Hangzhou, China; 7 Department of Pediatrics, Tongji Hospital, Tongji Medical College, Huazhong University of Science & Technology, Wuhan, China; 8 Department of Pediatric Rheumatology and Nephrology, Sun Yat-sen University First Affiliated Hospital, Guangzhou, China; 9 Department of Pediatrics, Peking University First Hospital, Beijing, China; 10 Department of Nephrology, Rheumatology and Immunology, Shanghai Children’s Hospital, Shanghai Jiao Tong University, Shanghai, China; 11 Department of Pediatrics, Jinling Hospital, School of Medicine, Nanjing University, Nanjing, China; 12 Department of Pediatric Nephrology, Shengjing Hospital of China Medical University, Shenyang, China; 13 Department of Pediatrics, Guangzhou First People’s Hospital, Guangzhou, China; 14 Department of Pediatric Nephrology, West China Second University Hospital of Sichuan University, Chengdu, China; 15 Department of Nephrology, Guangzhou Women and Children’s Medical Center, Guangzhou, China; 16 Department of Pediatrics, Shandong Provincial Hospital, Jinan, China; 17 Department of Nephrology and Immunology, Hebei Children’s Hospital, Shijiazhuang, China; 18 Department of Pediatric Rheumatology and Immunology, Guiyang Maternal and Child Health Hospital, Guiyang, China; 19 Department of Nephrology, Shanxi Children’s Hospital, Taiyuan, China; 20 Department of Nephrology, Wuhan Children’s Hospital, Wuhan, China; 21 Department of Pediatrics Nephrology, Second Xiangya Hospital of Central South University, Changsha, China; 22 Department of Nephrology, Xi’an Children’s Hospital, Xi’an, China; 23 The Centre of Evidence-based Social Science, School of Public Health, Lanzhou University, Lanzhou, China

**Keywords:** children, CKD-MBD, diagnosis, evaluation, guideline, treatment

## Abstract

Chronic Kidney Disease-Mineral and Bone Disorder (CKD-MBD) in children refers to the systemic mineral and bone metabolism disorders caused by CKD, including biochemical abnormalities, abnormalities in bone turnover, mineralization, quality, and ectopic calcification. Like the adult patients, mineral metabolism and bone structure abnormalities are universally present in children with CKD. But there have been no guidelines specifically for the children in the past. The medication treatment and clinical practices for children are usually based on those for adults, lacking targeted guidance. On the one hand, the children are in the process of growth and development, and the adult guidelines do not take into account the characteristics of children. On the other hand, the medication range for children is different from that of adults, and new drugs have been verified and tested in children. Hence, based on the developmental characteristics of children, combined with expert opinions from adults, children, kidney, nutrition, and medicine, this guideline was initiated by the Subspecialty Group of Nephrology, the Society of Pediatrics, Chinese Medical Association. It answered 15 important clinical questions related to the diagnosis and treatment of pediatric CKD-MBD, aimed to provide individualized treatment plans considering the clinical characteristics of CKD children and their treatment goals, while also addressing the needs of their growth and development.

## Introduction

Chronic kidney disease (CKD) refers to the chronic and persistent structural and functional impairment of the kidneys caused by various reasons. CKD-mineral and bone disorder (CKD-MBD) is a systemic mineral and bone metabolism disorder caused by CKD, including biochemical abnormalities, abnormalities in bone turnover, mineralization, quality, and ectopic calcification.^1^ Globally, the prevalence of CKD in children is approximately 14.9 to 118.8 per million population, and the end-stage renal disease (ESRD) is around 4.9 to 38.7 per million population.^2^ Like the adult patients, mineral metabolism and bone structure abnormalities are universally present in children with CKD. As the renal function declines, ectopic calcification gradually occurs. Studies have reported that 60% of children with ESKD present with ectopic calcification, most commonly in the vessels, lungs, kidneys, heart, and coronary arteries.^3^^,^^4^ Among all their peers, children with CKD have the highest risk of developing cardiovascular disease (CVD), which is closely associated with cardiovascular events and death, resulting in about 700 times the risk of death in children with CKD with CVD.^5^ At the same time, because children are in the process of growth and development, mineral and bone metabolism disorders caused by CKD may appear earlier than renal failure. Therefore, the management of CKD-MBD in children is crucial for the long-term prognosis of CKD. Moreover, because of the critical period of growth and development of children with CKD, there are specific characteristics in their diagnosis and treatment.

Therefore, based on the current best available evidence, the Subspecialty Group of Nephrology, the Society of Pediatrics in Chinese Medical Association initiated the development of the “Evidence-based Guideline for Diagnosis and Treatment of Pediatric CKD-MBD (version 2024)”, to answer the clinical key issues which concerned the healthcare professionals most and to further improve the comprehensive management of pediatric CKD in China. This guideline aims to provide individualized treatment plans considering the clinical characteristics of children with CKD and their treatment goals, while also addressing the needs of their growth and development.

The target population of this guideline is children aged 0 to 18 years with CKD, and the guideline applies to all children with CKD, but does not currently cover post-transplant recipients. The intended users are clinical physicians, clinical pharmacists, and medical laboratory staff from all medical institutions.

## Methodological approach

### Application Scope of the Guideline

This guideline applies to all children with CKD. The target audience for this guideline is clinical physicians, clinical pharmacists, and medical laboratory staff in various levels of medical institutions. This guideline focuses on the diagnosis, assessment, and treatment of mineral and bone abnormalities in children with CKD at all stages.

### Guideline Development Process

This guideline strictly followed the requirements of the World Health Organization Guideline Development Handbook^6^ and the Comprehensive Checklist for Successful Guidelines Enterprise version 2.0,^7^ designed and implemented guidelines development protocols, and complied with the 6 areas of the Appraisal of Guidelines for Research and Evaluation II (AGREE II).^8^ This guideline was written in accordance with the Reporting Items for Practice Guidelines in Healthcare (RIGHT).^9^ This guide has been registered in both Chinese and English on the International Practice Guide Registration Platform (2021CN086).

### Guideline Development Organization

**Figure 1:**
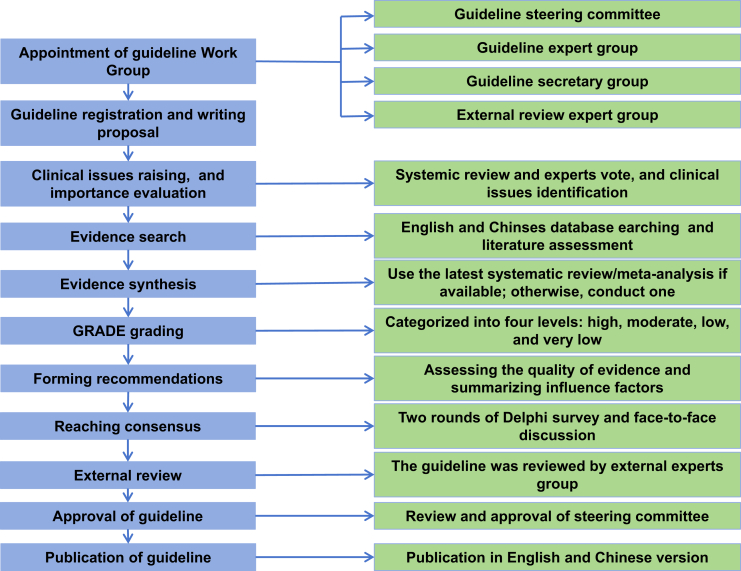
Flow diagram of developing this guideline. GRADE, Grading of Recommendations, Assessment, Development, and Evaluation.

This guideline was initiated by the subspecialty group of nephrology, the society of pediatrics, Chinese Medical Association. The methodological support was provided by the Guidelines and Standards Research Center of the Institute of Health Data Science in Lanzhou University, the Guidelines and Standards Research Center of the Chinese Medical Association Journal, the World Health Organization Collaborating Centre for Guideline Implementation and Knowledge Translation, the Grading of Recommendations, Assessment, Development, and Evaluation Center of Lanzhou University, and the Cochrane Lanzhou University Center (Figure 1).

### Appointment of the Guideline Work Group

The experts from the subspecialty Group of Nephrology, the Society of Pediatrics, Chinese Medical Association formed the steering committee. The steering committee appointed 3 chairs for the Guideline Work Group. Its main responsibilities included: (ⅰ) determining the theme and application scope of the guideline; (ⅱ) establishing expert group, secretary group, and external review expert group; (ⅲ) collecting, reviewing, and managing conflicts of interest; (ⅳ) comprehensive supervision guide development process; (ⅴ) approving the guideline proposal; (ⅵ) approving clinical issues; and (ⅶ) approving the final recommendations and the full text of the guideline.

The expert group was composed of multidisciplinary members from the pediatric field, including experts in nephrology, pharmacy, medical imaging, and guideline methodology. The responsibilities included the following: (ⅰ) forming clinical issues; (ⅱ) reviewing guideline proposal; (ⅲ) identifying population, intervention measures, control, and outcome of the clinical issues; (ⅳ) sorting the importance of outcome indicators; (ⅴ) guiding the secretary group to complete evidence collection and evaluation, evidence grading, and form decision tables; (ⅵ) reaching consensus on recommendations; and (ⅶ) revising the initial draft of the guideline and submitting it to the committee for review.

The secretary group was composed of members with backgrounds in pediatric clinical and evidence-based medicine research. The main responsibilities included the following: (ⅰ) completing the registration of the guidelines; (ⅱ) drafting guideline proposal; (ⅲ) conducting preliminary research on clinical issues related to CKD-MBD, designing questionnaire survey on clinical issues, collecting clinical issues, and ranking the importance of clinical issues; (ⅳ) completing literature search and evidence grading, and writing evidence summary table; (ⅴ) drafting the full text of the guideline; and (ⅵ) implementing the publication and promotion of guideline, and recording in detail the entire process of guideline development.

The external review expert group was composed of 3 to 5 experts in the fields of pediatric nephrology and clinical epidemiological methodology who did not directly participate in the development of the guideline. External experts reviewed the recommendations and preliminary guidelines and provided revision suggestions.

### Clinical Issues, Raising, Collection, Identification, and Importance Evaluation

The secretary group conducted an extensive literature review, including guidelines and systematic reviews related to CKD-MBD, initially generated clinical issues, developed a pool of clinical question issues, and submitted it to the steering committee for discussion. Initially, 25 clinical questions were included. After preliminary literature searching and discussion, the questions were excluded if they were considered unimportant by the steering committee or if their clinical evidence was very weak. Ultimately, 19 clinical questions were selected. Then, the importance evaluation questionnaire of clinical issues was formed. The secretary group sent the Delphi questionnaire survey to the members of the expert group. The questionnaire survey and importance assessment were completed using the 3-point Likert scale.^10^ Three points indicated that it was crucial for decision-making and recommendations, and should be included in the guideline; 2 points indicated that it was important; 1 point indicated that it was not important. If 75% of experts scored 3 points, it indicated that most experts believed that the question was of high importance and needed to be included in the guideline. If 75% of experts scored 1 point, it indicated that most experts believed that the importance of the issue was low, and it should not be included in the guideline. If 50% to 75% of experts scored 2 points, the second round of opinion collection was performed. The final clinical issues included in this guideline were formed based on the results of expert questionnaire surveys. According to the results of the questionnaire survey, 15 clinical questions were determined and subjected to the population, intervention, comparison, and outcome deconstruction.

### Search Strategy

The work group searched PubMed, Embase, Cochrane Library, Web of Science, Sinomed, CNKI, WanFang Database, and other Chinese and English databases, as well as the Guidelines International Network, National Institute for Health and Clinical Excellence, and Kidney Disease: Improving Global Outcomes (KDIGO) official websites. The words “chronic kidney disease,” “abnormal mineral and bone metabolism,” “diagnosis,” and “treatment” were search terms in both Chinese and English, and we combined relevant search equations with logical symbols (Supplementary Material A). The language was limited to Chinese or English, and the research types included systematic reviews or meta-analyses, randomized controlled trials (RCTs), cohort studies, case-control studies, etc. At the same time, manual supplementary searches were conducted on CKD-MBD-related guidelines, references included in the study, and grey literature (i.e., nonpublic publications such as nonpublic dissertations). The retrieval time was from the establishment of the database to December 2023.

### Evidence Profiles and Data Extraction

Three independent groups were established to screen the retrieved literature step by step based on the title, abstract, and full-text order. The screening and information extraction of each literature was independently completed by 2 individuals back-to-back. If there were any disagreements, they discussed and resolved them together or consulted with a member of the steering committee. Based on the predesigned data extraction table, the evidence profiles and data extract were completed.

### Risk Assessment of Literature Bias

A Systematic Review Assessment tool (AMSTAR) was used to assess the systematic reviews or meta-analyses,^11^ the Cochrane Collaboration’s tool was used to assess the RCTs,^12^ and the Newcastle Ottawa Scale was used to assess the cohort or case-control studies.^13^^,^^14^ The evaluation process was independently completed by 2 individuals. If there were any disagreements, they were discussed and resolved together or consulted with a member of the steering committee.

### Inclusion Criteria of the Literature

If the systematic review had been conducted within the past 5 years for a certain population, intervention, comparison, and outcome issue, and the AMSTAR score was 9 to 11 points, it indicated a low risk of bias. If multiple systematic reviews published within the past 5 years were found to score ≥ 9 points and the conclusions were consistent, the evaluation was based on the year of publication, the number and quality of included literature, key items, fitting degree with the corresponding population, intervention, comparison, and outcomes, comprehensiveness, etc. If the conclusions were inconsistent, the reasons for heterogeneity were explored and presented with different conclusions simultaneously. If the AMSTAR score were 0 to 4 points, it indicated a high risk of bias. If no systematic review was found after screening, high-quality RCTs were selected. If multiple RCTs had inconsistent conclusions, systematic reviews and meta-analyses were conducted. If the AMSTAR score was between 5 and 8 points, it indicated a moderate risk of bias. Further investigations were conducted to determine if there were high-quality RCTs available. If there was no systematic review or RCT, the observational studies were included.

### Grading of Evidence

The Grading of Recommendations Assessment, Development, and Evaluation was used to assess the quality of evidence for each clinical question and categorizes the quality of evidence into 4 levels as follows: high, moderate, low, and very low. RCTs were initially considered high-quality evidence, whereas observational studies were considered low-quality evidence. In the evidence grading process, 5 factors, including limitations, imprecision, inconsistency, indirectness, and publication bias, were downgrading factors, whereas 3 factors, including large effect size, dose-response relationship, and possible confounding factors (negative bias), were upgrading factors. Finally, a summary table of evidence was formed to present the evidence. The included guidelines have been evaluated by the Appraisal of Guidelines for Research and Evaluation Instrument II and the Reporting Items for Practice Guidelines in Healthcare checklist.

### Forming Recommendations and Reaching Consensus

The work group formed preliminary recommendations using the Grading of Recommendations Assessment, Development, and Evaluation decision table based on factors such as evidence quality, values, economic analysis, balance of advantages and disadvantages, and drug accessibility.^15^ Through 2 rounds of Delphi survey and further face-to-face discussion, a consensus was reached on the recommendations. The rules for reaching consensus were as follows: (ⅰ) if more than two-thirds of the experts participating in the consensus vote agreed with the recommendations, consensus was reached; (ⅱ) for the recommendations that had not reached consensus, a second round of expert consensus was conducted after modifications based on expert opinions until consensus was reached.

### External Review and Approval of Recommendations

The recommendations were reviewed by an external expert group before being submitted to the steering committee for approval. The work group had improved them based on their feedback and finally submitted them to the steering committee for approval.

### Promotion and Updating of the Guideline

After the guidelines are published, it will be promoted in the following ways: (ⅰ) dissemination through the official website of the journal publishing the guidelines, the official website of the sponsoring units, WeChat forwarding, etc.; (ⅱ) organizing nephrologists, laboratory physicians, radiologists, pediatricians, and other relevant medical staff to study the contents of the guidelines in a planned way nationwide; (ⅲ) introducing and promoting the guidelines at pediatric academic conferences; and (ⅳ) publishing articles on the interpretation of the guidelines for further dissemination.

The members of the guidelines work group plan to update this guideline 3 to 5 years after its publication, following the international guideline update process.

The protocol of this evidence-based guideline had been published in the Chinese Journal of Evidence-Based Pediatrics.^16^

### Definition and Categories of CKD

Childhood CKD is defined as the abnormality of kidney structure or function lasting for more than 3 months, and is classified as G1 to G5 based on glomerular filtration rate (GFR) categories. The criteria for CKD diagnosis are as follows (1 or more of the following conditions persisting for more than 3 months): (ⅰ) proteinuria (urine albumin excretion rate ≥ 30 mg/24 h; urine albumin creatinine ratio ≥ 30 mg/g); (ⅱ) abnormal urine sediment; (ⅲ) electrolyte and other abnormalities because of tubular disorders; (ⅳ) abnormal histological findings; (ⅴ) structural abnormalities detected by imaging; (ⅵ) history of kidney transplantation; (ⅶ) decline in GFR (< 60 ml/min per 1.73 m^2^) (GFR is classified as G3a–G5). The updated KDIGO guidelines apply to pediatric populations; however, the criterion of kidney injury duration exceeding 3 months does not apply to infants under 3 months of age. Additionally, the criterion of GFR < 60 ml/min per 1.73 m^2^ does not apply to children under 2 years of age. To date, there remains no consensus regarding the diagnostic criteria for CKD in children younger than 2 years (Table 1).

**Table 1:** Prognosis of CKD by GFR categories: KDIGO 2012^17^

GFR stages	GFR	Description
G1	≥ 90	Normal or increase
G2	60–89	Mild decrease
G3a	45–59	Mild-moderate decrease
G3b	30–44	Moderate-severe decrease
G4	15–29	Severe decrease
G5	< 15	Kidney failure

CKD, chronic kidney disease; GFR, glomerular filtration rate; KDIGO, Kidney Disease: Improving Global Clinical Outcomes; SCr, serum creatinine.

Considering the differences in height and gender of children, the estimated glomerular filtration rate formula mainly uses the Schwartz equation: estimated glomerular filtration rate (ml/min per 1.73 m^2^) = K × height × 88.4/ SCr. The SCr was determined by the picric acid method, and K is a constant, K = 0.33 for 0 to 28 days of children’s age, K = 0.45 for > 28 days and < 2 years, K = 0.55 for 2 to 12 years, and K = 0.77 for males and 0.55 for females over 12 years of age. Height is measured in centimeters, and SCr is measured in μmol/l.

## Summary of CKD-MBD Recommendations

### Section 1: Diagnosis of Pediatric CKD-MBD

Clinical question 1: Which serum biochemical indexes can be used to diagnose CKD-MBD in children?

1.1. We recommend serum calcium, phosphorus, alkaline phosphatase (ALP), intact parathyroid hormone (iPTH), and 25-hydroxyvitamin D_3_ (25[OH]D_3_) as the biochemical indicators for diagnosing CKD-MBD in children with CKD G2 to G5D (1B).
1.2. We recommend monitoring the above-mentioned biochemical indicators in children with CKD from stage 2, and adjusting the frequency of testing based on the stage of CKD, rate of progression, and medication treatment during the G2 to G5D period (1B).

Clinical question 2: How to evaluate linear growth disorder as one of the characteristic manifestations of CKD-MBD in children?

2.1. We suggest that infants aged 0 to 1 years with CKD G2 to G5D should have their length measured at least once a month, and children aged 1 to 3 years should have their linear growth evaluated at least every 3 months. Children older than 3 years should have their linear growth evaluated at least every 6 to 12 months (not graded).

Clinical question 3: Which biochemical indicators can be used to assess the nature and severity of bone abnormalities in children with CKD-MBD?

3.1. In children with CKD G3a to G5D, it is recommended to monitor iPTH and bone alkaline phosphatase (BAP) together to assess the nature and severity of bone abnormalities (1B).
3.2. Based on the characteristics of children’s growth and development and the disease features of pediatric CKD-MBD, measurement of the carboxy-terminal fibroblast growth factor23 (cFGF23) may be considered as an early indicator of mineral metabolism disturbances in settings where this assay is available. (2B).

Clinical question 4: What are the appropriate imaging techniques for detecting changes in bone mineral density (BMD) and bone mass in children with CKD-MBD?

4.1. It is reasonable to consider dual-energy x-ray absorptiometry (DXA) for BMD to assess bone quality and fracture risk for children with CKD G3a to G5D (2C).

Clinical question 5: Is bone histological examination necessary for children with CKD-MBD?

5.1. If it is necessary to determine or adjust treatment decisions by understanding the type of renal osteodystrophy (ROD), bone biopsy might be performed for children with CKD G3a to G5D (2C).

Clinical question 6: How to detect vascular calcification in children with CKD-MBD?

6.1. For selected children with CKD G3a to G5D at high risk for cardiovascular complications, computed tomography (CT) examination may be considered to assess coronary artery and abdominal aortic calcification when the results are expected to meaningfully influence clinical management. However, CT is not recommended for routine screening because of radiation exposure concerns (2D).
6.2. In children with CKD G3a to G5D, lateral abdominal radiograph and echocardiogram can be used to detect the presence or absence of valvular calcification. These are reasonable and preferred alternatives to CT-based imaging in most clinical situations (not graded).

### Section 2: Management of Pediatric CKD-MBD

Clinical question 7: What are the normal ranges of serum calcium and phosphorus in children with CKD G2 to G5D at different ages?

7.1. We suggest that for children in CKD G2 to G5D, to maintain serum calcium and phosphorus within the normal range of the corresponding age (not graded).

Clinical question 8: What is the recommended range of daily intake of dietary calcium and phosphorus for children with CKD G3a to G5D?

8.1. We suggest that the total daily intake of elemental calcium (including phosphate binders) in children with CKD G2 to G5D should not exceed twice the dietary reference intakes (DRI) (not graded).
8.2. We suggest that children with CKD G3a to G5D should not exceed their daily DRI intake of phosphorus while ensuring protein intake. For children with elevated levels of serum phosphorus and iPTH, it is recommended that the intake of dietary phosphorus should not exceed 80% of DRI at the same age (not graded).

Clinical question 9: What is the appropriate calcium supplementation strategy for hypocalcemia in children with CKD G3a to G5D?

9.1. For the children with CKD G3a to G5D, when dietary intake is insufficient to meet the DRI requirements and/or when hypocalcemia is concomitantly present, the administration of calcium supplementation is recommended (1C).

Clinical question 10: How to choose phosphate-lowering drugs for children with CKD G3a to G5D who develop hyperphosphatemia?

10.1. For the children with CKD G3a to G5D and serum calcium levels below the age-specific normal upper limit, it is recommended to use calcium-based phosphate binders (1B) and/or combined with noncalcium metal-based phosphate binders (1B), nonmetal-based phosphate binders (1A) for the treatment of hyperphosphatemia.
10.2. If the child has comorbidities such as persistent hypercalcemia or arterial calcification, low iPTH levels, or dystrophic bone disease, the use of calcium-based phosphate binders should be restricted (1B).

Clinical question 11: How to adjust the dialysis regimen for hypocalcemia or hyperphosphatemia in children with CKD G5D?

11.1. In the children with CKD G5D, when the ionized calcium concentration falls below 1.25 mmol/l, individualized adjustments to the calcium-containing dialysate prescription should be considered. Close monitoring of serum calcium levels during treatment is advisable (not graded).
11.2. For the children with persistent hyperphosphatemia in CKD G5D, implementing an intensified dialysis regimen may be considered to enhance the removal of phosphate from the blood (2D).

Clinical question 12: What is the recommended range of serum vitamin D concentration for the children with CKD G2 to G5D?

12.1. We recommend that the serum 25-hydroxy (OH)D concentration for the children with CKD G2 to G5D should be maintained above 30 ng/ml (> 75 nmol/l) (not graded).
12.2. We recommend supplementing natural vitamin D for children with CKD G2 to G5D who have serum 25(OH)D levels below 30 ng/ml, and selecting the appropriate regimen based on age and severity of deficiency (not graded).

Clinical question 13: What is the recommended range for iPTH levels in the children with CKD G3a-G5D?

13.1. We suggest for children with CKD G2 to G3, it is recommended to control iPTH levels within the normal range. For children with CKD G4, iPTH levels should be controlled below twice the upper limit of the normal range. For children with CKD G5 to G5D, it is recommended to maintain iPTH levels between 2 to 9 times the upper limit of the normal range (not graded).

Clinical question 14: How to supplement active vitamin D and vitamin D analogues in the children with CKD G3a to G5D when they have secondary hyperparathyroidism (SHPT)?

14.1. For children with CKD G3 to G5D who have severe and progressive SHPT, we recommend to start initial oral calcitriol therapy to reduce excessively high iPTH levels (1B).
14.2. When the total dose of weekly oral calcitriol is consistent, oral calcitriol can be administered daily or intermittently, with an initial dose of 10 to 20 ng/kg/d, to control iPTH levels within a reasonable range (1B).
14.3. For the children with mild SHPT or stable iPTH levels in CKD G3 to G5D, we suggest oral treatment with vitamin D analogues. For patients with CKD, we recommend oral administration of alfacalcidol (1C) and paricalcitol (1B) to control iPTH levels.

Clinical question 15: How to supplement calcimimetics in the children with CKD G3a to G5D when they have SHPT?

15.1. The combination of cinacalcet, calcitriol, or vitamin D analogues could be considered for children with CKD G4 to G5D who have persistent iPTH levels above 300 pg/ml in order to effectively control iPTH within the target range (2C).
15.2. The treatment for children with CKD G4 to G5D and persistently elevated iPTH levels (> 300 pg/ml) could initiate cinacalcet therapy at a low dose (≤ 0.20 mg/kg/d), with subsequent dosage adjustments based on treatment efficacy, up to a maximum daily dose of 60 mg (2C)

## Section 1: Diagnosis of Pediatric CKD-MBD

### Clinical Question 1: Which Serum Biochemical Indexes can be Used to Diagnose CKD-MBD in Children?

#### Key Takeaways

• Core diagnostic panel: Serum calcium, phosphorus, ALP, iPTH, and 25-hydroxyvitamin D_3_ are the essential biochemical indicators for diagnosing CKD-MBD.
• Early monitoring required: Monitoring of these indicators should begin at CKD stage 2.
• Adjustable testing frequency: The frequency of testing should be individualized based on CKD stage, rate of disease progression, and whether the child is receiving treatment.

1.1 We recommend serum calcium, phosphorus, ALP, iPTH, and 25-hydroxyvitamin D_3_ (25[OH]D_3_) as the biochemical indicators for diagnosing CKD-MBD in children with CKD G2 to G5D (1B; see Supplementary Tables S1–S5).
1.2 We recommend monitoring the above-mentioned biochemical indicators in children with CKD from stage 2, and adjusting the frequency of testing based on the stage of CKD, rate of progression, and medication treatment during the G2 to G5D period (1B; see Supplementary Tables S1–S5).

#### Rationale

Serum phosphorus concentration is influenced by renal excretion, and phosphorus retention caused by CKD leads to hyperphosphatemia. Phosphorus combines with calcium in the blood to form calcium phosphate deposits in soft tissues, resulting in decreased serum calcium levels and inhibiting the production of 1-hydroxylase in the proximal tubules, leading to a decrease in the level of 1,25-(OH)_2_D_3_ (calcitriol) and an increase in the iPTH by the parathyroid gland.^18^ Although children with early-stage CKD may not have clinical manifestations of bone and cardiovascular disease, their serum biochemical indicators may be abnormal.

A *post hoc* analysis study from Europe in 2018 included 2 clinical cohorts of 80 children with CKD and vitamin D deficiency from Germany and the UK. At the time of inclusion in the study, children with various stages of CKD had baseline abnormalities in calcium, phosphorus, and iPTH levels. 46% to 49% of patients had low serum calcium, 26% to 44% had hyperphosphatemia, and serum 25(OH) D was decreased (46.1–50.8 nmol/L). iPTH levels were normal or elevated, and the median BAP level was 101 to 103U/l.^19^ A global multicenter cohort study including children with CKD (*n* = 556) in 2015 showed that 12.2% to 14.6% of patients with CKD G3 to G5 had hypocalcemia, 18.4% to 65.9% had hyperphosphatemia, and mean serum iPTH was elevated (9.2–17.8pmol/ml), whereas mean serum 25(OH) D was decreased (9.2–12.4ug/l).^20^ The above study showed that children with CKD G3 already had significant abnormalities in biochemical markers such as serum calcium, phosphorus, ALP, iPTH, and 25(OH)D_3_. Therefore, we recommend using these markers to diagnose children with CKD-MBD (Supplementary Tables S1–S5).

The updated 2017 KDIGO guidelines recommend monitoring serum calcium, phosphorus, parathyroid hormone (PTH), and ALP levels in children with CKD G2.^17^ The European Pediatric Dialysis Working Group guidelines recommend monitoring the indicators from CKD G3.^21^ In addition, the evidence included in this guideline indicates that children with CKD G2 to G3 already experience significant manifestations of hypocalcemia, hyperphosphatemia, and elevated iPTH.^19^^,^^20^ Therefore, this guideline recommends continuous monitoring of serum calcium, phosphorus, iPTH, and ALP levels starting from CKD G2.

There is no data-based evidence retrieved in this guideline regarding the frequency of biochemical indicator monitoring in children with CKD. However, multiple guidelines have recommended the frequency of monitoring biochemical indicators in children with CKD. Taking into account factors such as the specific target group, feasibility of clinical practice, and level of regional economic development, it mainly referred to the KDIGO guidelines (2017),^17^ European Renal Association–European Dialysis and Transplant Association guidelines (2021),^22^ and European Pediatric Dialysis Working Group guidelines (2006)^21^ to make the following recommendations (Table 2). However, in clinical practice, the monitoring frequency still needs to be adjusted according to the baseline level of the child and the treatment plan.

**Table 2:** Recommended frequency of biochemical indicator testing for children with CKD G2-G5D

Parameter	CKD G2	CKD G3	CKD G4	CKD G5-G5D
Serum calcium	6 mos	6 mos	3 mos	1 mo
Serum phosphorus	6 mos	6 mos	3 mos	1 mo
ALP	12 mos	6 mos	3 mos	1–3 mos
iPTH	12 mos	6 mos	3 mos	1–3 mos
25(OH)D_3_	12 mos	6 mos	3 mos	1–3 mos

ALP, alkaline phosphatase; iPTH, intact parathyroid hormone; CKD, chronic kidney disease.

For patients receiving active vitamin D analogues or calcimimetics, more frequent monitoring of calcium and iPTH may be required (within 2–4 weeks of treatment initiation or dose change). The frequency should be individualized based on the child’s baseline values, rate of CKD progression, and specific treatment plan.

### Clinical Question 2: How to Evaluate Linear Growth Disorder as one of the Characteristic Manifestations of CKD-MBD in Children?

#### Key Takeaways

• Growth assessment is essential: Linear growth measurement is a critical tool for evaluating CKD-MBD in children, as growth retardation and skeletal deformities are unique clinical features of pediatric CKD.
• Early intervention depends on early detection: Regular growth monitoring enables timely intervention, which is crucial for improving final height in children with CKD and short stature.

2.1 We suggested that infants aged 0 to 1 years with CKD G2 to G5D should have their length measured at least once a month, and children aged 1 to 3 years should have their linear growth evaluated at least every 3 months. Children older than 3 years should have their linear growth evaluated at least every 6 to 12 months (not graded).

#### Rationale

The bone abnormalities in children with CKD-MBD can have various manifestations, including growth retardation, ostealgia, skeletal deformities, epiphyseal slipping, and fractures. Among them, growth retardation and skeletal deformities are specific clinical manifestations unique to children with CKD.^23^ The guidelines published by the European Society for Pediatric Nephrology CKD–MBD, dialysis and transplantation working groups in 2019 recommended that the children with CKD should be considered as short stature if their height is below the third percentile for the age and sex, or the height standard deviation score is below −1.88.^24^ Early intervention is crucial for improving the final height of children with CKD with short stature. Therefore, regular assessment of skeletal growth is necessary, and the simplest assessment method is measurement of length or height.

Several guidelines recommend the frequency of length or height measurement in children with CKD. This guideline mainly recommends growth monitoring frequency based on the 2019 European Society for Pediatric Nephrology guidelines^24^ and the 2009 Kidney Disease Outcomes Quality Initiative (KDOQI) guidelines.^25^ Because of the characteristic rapid growth during infancy and adolescence, monitoring frequency should be appropriately increased. Therefore, this guideline suggests that infants aged 0 to 1 years with CKD G2 to G5D should have their length measured at least once a month, children aged 1 to 3 years should have their linear growth evaluated at least every 3 months,and children older than 3 years should have their linear growth evaluated at least every 6 to 12 months.

### Clinical Question 3: Which Biochemical Indicators can be Used to Assess the Nature and Severity of Bone Abnormalities in Children With CKD-MBD?

#### Key Takeaways

• Combined biomarkers improve assessment: For children with CKD G3a to G5D, it is recommended to monitor both iPTH and BAP together to evaluate the nature and severity of bone abnormalities. Although iPTH reflects bone turnover, it is insufficient to distinguish low-turnover from high-turnover disease; BAP significantly improves diagnostic accuracy, particularly for identifying low bone turnover.
• Fibroblast growth factor 23 (FGF23) as an early indicator (when available): FGF23 plays a key role in phosphate regulation and CKD-MBD progression. Where feasible, early monitoring of FGF23 (specifically the cFGF23) may aid in detecting early mineral disturbances and tracking growth and development in children with CKD. However, its use is currently limited by cost and availability, and further research is needed.

3.1 In children with CKD G3a to G5D, it is recommended to monitor iPTH and BAP together to assess the nature and severity of bone abnormalities (1B; see Supplementary Tables S6–S10).
3.2 Based on the characteristics of children’s growth and development and the disease features of pediatric CKD-MBD, measurement of cFGF23 may be considered as an early indicator of mineral metabolism disturbances in settings where this assay is available (2B; see Supplementary Tables S6–S10).

#### Rationale

The bone metabolism process includes 2 circular processes— bone formation and bone resorption. Various biochemical indicators of bone metabolism can reflect the mineral and bone metabolic abnormalities caused by CKD, but most of these circulating biomarkers have short half-lives, and the bone remodeling process takes a long time (3–6 months). Therefore, there is currently no ideal biochemical indicator to assess bone abnormalities in CKD-MBD. Importantly, bone turnover rates vary significantly with age in children. During infancy and adolescence, physiological bone turnover is normally elevated to accommodate rapid skeletal growth and development.

Phosphate retention and/or decreased synthesis of 1,25(OH)_2_ vitamin D can cause an increase in PTH and further contribute to bone abnormalities in CKD-MBD. Therefore, iPTH is a common indicator used in clinical practice to reflect PTH levels, which is mainly used to assess bone turnover and is often used to diagnose high-turnover bone disease.^26^

A single-center retrospective study from Germany in 2018 included 105 patients who underwent bone biopsies. Among them, 18.1% of the patients had normal renal function, 31.4% were in CKD G3 to G5 not on dialysis (CKD NOD group), and 50.5% were in CKD G5 and required hemodialysis (HD) (CKD 5D group). The patients in the CKD NOD group and CKD 5D group were divided into high-turnover ROD and nonhigh-turnover ROD. The level of iPTH in high-turnover ROD was significantly higher than that in nonhigh-turnover ROD (26 ± 18 vs. 8 ± 9 pmol/l, *P* < 0.001), whereas BAP was not significantly different (19.4 ± 11.3 vs. 25.7 ± 17.5 pmol/l, *P* = 0.350). In the CKD NOD group, the level of iPTH in high-turnover ROD was significantly higher than that in nonhigh-turnover ROD (32 ± 44 vs. 8 ± 11 pmol/l, *P* = 0.001), and BAP was also significantly higher (38.5 ± 31.8 vs. 16.0 ± 6.9 pmol/l, *P* = 0.01).^27^ The above evidence indicates that high iPTH levels are associated with high bone turnover. However, some studies suggest that iPTH levels may not fully correspond to the patient’s bone transport status. An RCT conducted in Brazil in 2008 included 97 patients on HD, grouped according to their iPTH levels. The results showed that the proportion of patients with low-turnover bone disease was highest in the < 150 pg/ml and 150 to 300 pg/ml groups (29/35, 83%; 14/22, 64%), whereas in the > 300 pg/ml group, high-turnover bone disease was most common (25/40, 62.5%), followed by low-turnover bone disease (15/40, 37.5%). Statistical analysis found that the efficiency of predicting low-turnover bone disease was 83% when the iPTH level was < 150 pg/ml, but the efficiency of predicting high-turnover bone disease was only 62% when the iPTH level was > 300 pg/ml.^28^ This indicates that relying solely on iPTH levels to determine a patient's bone turnover status is not comprehensive enough.

BAP is an osteoblast enzyme secreted by osteoblasts, which is a sensitive and specific biomarker for bone formation and bone remodeling. It can accurately reflect the state of bone absorption. A cross-sectional study in 2018 in the UK enrolled 43 adult patients with CKD G4 to G5 to explore the accuracy of ROD bone turnover biomarkers. Bone biopsies revealed that 26% of patients had low bone turnover, 34% had normal bone turnover, and 40% had high bone turnover. Simultaneously, the bone biochemical markers had been detected in these patients, and the results showed that the positive predictive value of iPTH for high bone turnover was 90%, whereas the positive predictive value of BAP was 69%. However, in the receiver operating characteristic (ROC) analysis of distinguishing the low and nonlow bone turnover, the area under the curve (AUC) of BAP was 0.824 (positive predictive value: 53%), which was significantly better than that of iPTH (0.563, positive predictive value: 32%).^29^ Therefore, BAP is significantly superior to iPTH in distinguishing between low and nonlow bone turnover. Based on the available evidence and the recommendations from the 2017 KDIGO guidelines, we recommend combining iPTH and BAP to determine the type of bone turnover and assess the severity of bone disease.

FGF23 is mainly expressed by bone cells surrounding the osteocytes, and its main target organ is the kidney. It regulates phosphate reabsorption by binding to the Klotho-fibroblast growth factor receptor complex in the kidney and inhibits the production of 1,25(OH)_2_D_3_. FGF23 is closely related to the development and prognosis of CKD-MBD. A cohort study conducted in Europe in 2015 included 556 children with CKD G3 to G5, and the cFGF23 level was quantitatively determined by an enzyme-linked immunosorbent assay kit. The results showed that compared with the normal control group, the FGF23 level was significantly higher in children with CKD, and there was a positive correlation with the stage of CKD. The FGF23 levels were 128 kRU/l (89– 221) for CKD G3, 226 kRU/l (136– 355) for CKD G4, and 654 kRU/l (321–1224) for CKD G5 (*P* < 0.0001).^20^ The study shows that as children with CKD progress from G3 to G5, there is an increase in FGF23 levels, suggesting that FGF23 may serve as an early indicator of mineral metabolism disturbances in CKD-MBD. Current evidence in children primarily uses cFGF23 assays. Considering the cost, limited availability, lack of standardized pediatric reference ranges, and uncertainty regarding which form best reflects clinical outcomes, FGF23 measurement is not recommended for routine clinical monitoring at present. Its use should be limited to research settings or specialized clinical contexts where results may inform individualized management decisions. Further research is needed to establish pediatric reference ranges for both assay types, determine the clinical utility of FGF23 monitoring in guiding therapy, and explore the relationship between FGF23 levels and long-term outcomes in children with CKD-MBD (Supplementary Tables S6–S10).

Except for iPTH, BAP, and FGF23, a prospective, multicenter study in Europe found that calcium isotope ratios may provide a novel, sensitive, and noninvasive method of assessing bone calcium balance in CKD.^30^ In future, the predictive value of bone turnover markers coupled with calcium isotope ratios may be considered together for a comprehensive assessment of bone health in patients with CKD.

### Clinical Question 4: What are the Appropriate Imaging Techniques for Detecting Changes in BMD and Bone Mass in Children With CKD-MBD?

#### Key Takeaways

• DXA is the preferred imaging tool: DXA is recommended for assessing BMD in children with CKD G3a to G5D to evaluate bone strength and fracture risk. It is widely available, low-cost, and involves minimal radiation exposure.
• Height adjustment is essential: In children with growth retardation, DXA Z-scores should be corrected for height or apparent bone density to avoid misinterpreting low scores as bone defects rather than growth-related artifacts.
• Correlation with clinical markers: DXA-derived BMD Z-scores correlate negatively with serum iPTH, phosphorus, and BAP, and positively with serum calcium, supporting its utility in monitoring bone health.
• Limitations and future needs: DXA cannot distinguish between cortical and trabecular bone or assess microarchitecture. High-quality pediatric studies are needed to better establish the link between DXA findings and clinical outcomes, such as fracture risk.

4.1 It is reasonable to consider DXA for BMD to assess bone quality and fracture risk for children with CKD G3a to G5D (2C; see Supplementary Tables S11–S15).

#### Rationale

Changes in BMD and quality during the growth process of children with CKD can lead to fractures, skeletal deformities, and chronic pain. Studies have shown that children have a significantly higher risk of fractures compared with healthy children of the same gender.^31^ In addition, CKD-MBD occurring during childhood can result in severe sequelae in adulthood, such as severe short stature, severe bone disease (deformity, bone pain, aseptic necrosis, and nontraumatic fractures), and disability caused by bone abnormalities.^32^ Therefore, it is crucial to monitor bone abnormalities and treatment response in children with CKD.

At present, the main methods of detecting BMD include DXA, peripheral quantitative CT, quantitative ultrasound, X-ray, etc. Among them, there is little international research on peripheral quantitative CT, ultrasound, and X-ray in children. Because of its advantages, such as low cost, accessibility, low compatibility requirements, and low radiation,^33^ DXA is widely used for the evaluation of BMD, and is the only internationally recognized method for bone mineral content and BMD, as well as for diagnosing osteoporosis.

In 2020, a multicenter cross-sectional study in Europe and the United States used DXA to evaluate BMD in children with CKD, including 26 children with CKD G4 to G5 and 77 children on dialysis. The results showed that 58% of patients had severe bone pain that affected their daily lives, and 10% of patients had at least 1 low-trauma fracture. Compared with the CKD group, the DXA BMD-Z scores of the lumbar spine and hip in the dialysis group were significantly lower (*P* < 0.01).^34^ A cross-sectional study in Egypt in 2004 also used DXA to assess BMD in 21 predialysis children and 44 children on HD. The results showed that 61.9% of predialysis children (*n* = 13) and 59.1% of children on HD (*n* = 26) had bone mineral content and density reduction. The BMD-Z score of the lumbar spine in children is negatively correlated with their serum iPTH (*r* = −0.47, *P* = 0.03), serum phosphorus (*r* = −0.61, *P* = 0.004), and BAP (*r* = −0.52, *P* = 0.02) levels, and positively correlated with serum calcium (*r* = 0.41, *P* = 0.07).^35^ In 2023, a case-control observational study in Egypt evaluated the usefulness of trabecular bone score and found that it is significantly reduced in children on maintenance HD and is associated with increased fracture incidence.^36^ The above research results suggest that DXA can effectively evaluate the BMD of children with CKD. The International Society for Clinical Densitometry recommends performing lumbar spine and whole-body, less head, DXA scans for children with chronic diseases that may affect bone health (Supplementary Tables S11–S15).^37^

However, the specific growth disorder in children with CKD-MBD may affect the accuracy of DXA in assessing BMD. Because of the dependence of DXA on area density rather than volumetric density, it is unable to distinguish between trabecular and cortical bone that are independently damaged in CKD, and it cannot assess trabecular microstructure. Therefore, in children with low height, low DXA Z scores may be attributed to growth disorders rather than true bone defects.^38^ A study evaluated the lumbar BMD in children with CKD G4 to G5 and corrected the Z score based on height. After correction, the lumbar BMD-Z score of the children increased compared with the previous value (the average Z score increased from −0.03 to 0.49). Therefore, appropriate correction of the Z score of children with CKD accompanied by growth retardation based on height or apparent bone density can reduce the error in bone density assessment to a certain extent.^39^

In 2017, the KDIGO guidelines recommended that for CKD G3a to G5D patients with existing CKD-MBD and/or osteoporosis risk factors, if the test results affect treatment decisions, BMD testing is recommended to assess fracture risk. Currently, there is little evidence in children that low BMD detected by DXA can predict fracture risk, and for children with CKD who have growth retardation, the BMD-Z score should be corrected based on height or apparent BMD.^40^, ^41^, ^42^ In 2021, the European Society for Pediatric Nephrology considered that the use of radiological methods to assess bone disease in children with CKD-MBD still needs to be weighed against the risks and benefits, especially in terms of radiation exposure.^22^

In summary, for children with CKD G3a to G5D, DXA for BMD testing may be used to assess bone strength and predict fracture risk. DXA provides valuable information on bone mineral content and density, which can aid in clinical decision-making regarding the need for interventions to improve bone health. The results should be corrected based on height or apparent bone density. However, because of the lack of high-quality evidence on the correlation between DXA test results and clinical outcomes in children with CKD-MBD, further large-scale prospective studies are needed to further explore this issue.

### Clinical Question 5: Is Bone Histological Examination Necessary for Children With CKD-MBD?

#### Key Takeaways

• Bone biopsy remains the gold standard: Bone histomorphometry is the definitive method for assessing bone turnover, mineralization, and volume in pediatric CKD-MBD, and is essential for diagnosing ROD subtypes.
• Reserved for specific indications: Because of its invasive nature, cost, and need for specialized expertise, bone biopsy is not recommended for routine use. It should be considered only when understanding the ROD type is necessary to guide or adjust treatment decisions—particularly in cases of unexplained bone disease, refractory hypercalcemia, persistent hypophosphatemia, skeletal deformities, or fractures.
• Pediatric bone disease has unique features: Unlike in adults, mineralization defects are common in children with CKD and may increase with disease progression. Recent studies also show a rising proportion of low-turnover bone disease, likely related to increased use of vitamin D analogues.

5.1 If it is necessary to determine or adjust treatment decisions by understanding the type of ROD, bone biopsy might be performed for children with CKD G3a to G5D (2C; see Supplementary Table S16–S20).

#### Rationale

CKD-MBD bone disease can be evaluated by histomorphometric analysis.^43^ Bone histomorphometry can accurately determine bone turnover, mineralization, and bone volume status, and is the gold standard for the diagnosis of metabolic bone diseases. Multiple studies have shown that approximately half of children with CKD have high-turnover bone disease, whereas low-turnover bone disease only accounts for about 10% to 20%.^44^, ^45^, ^46^, ^47^, ^48^, ^49^, ^50^, ^51^ A cross-sectional study conducted in the United States in 2016 and another study by Carvalho *et al.*^52^ in the United States in 2015 analyzed bone biopsy results from 68 children with ESRD and 22 dialysis children, respectively. The results showed that 76% to 77% of the patients (*n* = 64) had normal or high bone turnover, whereas 23% to 24% of the patients (*n* = 26) had low bone turnover.^51^^,^^52^ However, with the widespread use of calcitriol analogues and vitamin D analogues, the proportion of low-turnover patients has been increasing over the past 2 decades. A retrospective study conducted in Brazil in 2020 (*n* = 42) showed that 59.5% of dialysis children (*n* = 25) had low bone turnover, 16.7% (*n* = 7) had high bone turnover, and 23.8% (*n* = 10) had normal bone turnover.^53^

Besides bone turnover abnormalities, most children with CKD also have skeletal mineralization abnormalities. A cross-sectional study conducted in the United States in 2012 analyzed bone biopsy results from 52 patients with CKD (aged between 2–21 years) and found that all patients with CKD G2 to G5 had varying degrees of mineralization defects, indicating a high incidence of bone mineralization defects in children with CKD and an increase in the prevalence of abnormal mineralization and bone turnover with the progression of CKD.^54^ A retrospective study conducted in the United States in 2010 analyzed bone biopsy results from 161 dialysis children and found that 48% of the children had mineralization abnormalities.^50^ Some studies have shown that mineralization defects are not common in adults receiving maintenance dialysis treatment,^55^^,^^56^ and mineralization defects may be a specific feature of CKD-MBD children (Supplementary Tables S16–S20).

Currently, the gold standard for measuring bone turnover and mineralization is still bone biopsy, which can help develop targeted treatment plans for refractory hypercalcemia, unexplained hypophosphatemia, skeletal deformities, and fractures. As a bone biopsy is an invasive examination with high costs and requires professional operators, it is not currently a routine test for CKD-MBD patients. Following the KDIGO 2017 CKD-MBD guidelines, this guideline recommends that if children with CKD G3a to G5D have clinical and biochemical manifestations of unexplained bone disease or ROD types that affect treatment decisions, consideration can be given to bone biopsy.^57^ Future pediatric studies need to be conducted to explore the correlation between the bone lesion biomarkers and histological examination results in children with CKD-MBD.

### Clinical Question 6: How to Detect Vascular Calcification in Children With CKD-MBD?

#### Key Takeaways

• CT is not for routine screening: CT offers higher sensitivity for detecting vascular calcification but involves significant radiation exposure. Its use should be reserved for select, high-risk cases where results are expected to directly influence clinical management.
• Risk-Benefit considerations in children: The decision to use CT must account for children's greater radiosensitivity, potential need for sedation, lower pretest probability of detectable calcification, and limited evidence that routine screening improves outcomes.
• Evidence base: Studies confirm that both X-ray and CT can detect vascular calcification, and echocardiography effectively identifies valvular calcification in patients with CKD. However, pediatric-specific evidence for lateral abdominal X-ray and echocardiography remains limited, warranting further research.

6.1 For selected children with CKD G3a to G5D at high risk for cardiovascular complications, CT examination may be considered to assess coronary artery and abdominal aortic calcification when the results are expected to meaningfully influence clinical management. However, CT is not recommended for routine screening because of radiation exposure concerns (2D).
6.2 In children with CKD G3a to G5D, lateral abdominal radiograph and echocardiogram can be used to detect the presence or absence of valvular calcification. These are reasonable and preferred alternatives to CT-based imaging in most clinical situations (not graded).

#### Rationale

Patients with CKD not only commonly experience abnormalities in mineral metabolism and bone structure, but also develop extrarenal calcifications because of excessive pathological deposition of calcium phosphate salts and decreased calcification inhibitors, involving vascular, pulmonary, renal, cardiac, and other soft tissue sites. The proportion of children with ESRD who develop extrarenal calcifications can be as high as 60%.^3^^,^^4^^,^^58^ With the progression of CKD, the risk of vascular calcification increases, which is closely related to the increased cardiovascular disease and all-cause mortality in patients with CKD.^59^, ^60^, ^61^, ^62^, ^63^

In 2021, a multicenter cross-sectional study in the UK included 100 children with ESRD (23 children with CKD G4 to G5 and 77 children on dialysis), and evaluated the coronary artery calcification by cardiac CT. The results showed that the median age of all patients was 13.82 years (inter quartile range [IQR]: 10.68–16.46 years), and 10 patients were diagnosed with coronary artery calcification (CAC) (9 patients in the dialysis group and 1 patient in the CKD group). No difference was found in age, dialysis time, or serum biomarkers between CAC patients and non-CAC patients.^64^ After a median follow-up of 1.45 years, the study followed up 57 patients who were still under observation, with a median age of 15.84 years (IQR: 12.56–21.69 years). Among them, 5 patients were found to have CAC in the first study, and among the 18 patients who had CT re-examination in the second study, a total of 10 patients were diagnosed with CAC. The average CAC score increased from 8.1 (range 0–412.6) at baseline to 42.61 (range 0–491.0).^65^ A systematic review of adult abdominal aortic calcification assessment in 2017 included 33 cross-sectional studies and 11 cohort studies with 9883 patients undergoing dialysis. CT, X-ray, or ultrasound was used to evaluate abdominal aortic calcification. The results showed that the prevalence of abdominal aortic calcification ranged from 19% to 95%, with a combined prevalence 68.5%. Because of the influence of different regions, testing equipment, patient age, and male proportion, there was significant heterogeneity among the studies (total variation [I^2^] = 97.5%, *P* < 0.001). For diagnostic instruments, the prevalence of abdominal aortic calcification reported in 6 CT studies was significantly higher than that reported in 37 plain X-ray studies (84.9% vs. 65.2%), and the heterogeneity of CT studies was lower than that of X-ray studies.^61^ These studies indicate that both X-ray and CT can detect and diagnose abdominal aortic calcification, while CT has higher sensitivity and stability in detecting vascular calcification. However, for routine clinical practice in children, the favorable safety profile and widespread availability of lateral abdominal X-ray and echocardiography make them the preferred initial imaging choices. In 2018, a multicenter prospective cohort study in China included 1497 patients with CKD who had received HD/peritoneal dialysis treatment for at least half a year and used echocardiography to evaluate valve calcification. The results showed that valve calcification occurred in 77.4% of patients and valvular heart disease was present in 29.0% of patients (Supplementary Tables S21–S25).^66^

In the updated KDIGO guidelines published in 2017, for the patients of stages G3a to G5D, it is suggested that a lateral abdominal radiograph can be used to detect the presence or absence of vascular calcification, and an echocardiogram can be used to detect the presence or absence of valvular calcification, as reasonable alternatives to CT-based imaging.

Heart ultrasound has no radiation, is cost-effective, widely acceptable, and has good sensitivity. It can be used as an alternative to CT for detecting valve calcification in patients with CKD. But there is currently no evidence for echocardiography in detecting valve calcification in children with CKD, which needs to be further explored. Overall, the lateral abdominal X-ray has good sensitivity in detecting vascular calcification, compared with CT, which is more expensive. However, the prevalence of vascular calcification in children is lower than that in adults, but there are no relevant reports on the application of lateral abdominal X-ray in children with CKD, and its advantages and disadvantages still need to be discussed.

## Section 2: Treatment of pediatric CKD-MBD

### Clinical Question 7: What are the Normal Ranges of Serum Calcium and Phosphorus in Children with CKD G2 to G5D at Different Ages?

#### Key Takeaways

• Calcium management: Maintain age-appropriate normal range. Both deficiency (impaired bone mineralization) and excess (ectopic calcification, cardiovascular risk) must be avoided.
• Phosphorus management: Maintain age-appropriate normal range. Hyperphosphatemia increases cardiovascular risk via oxidative stress, elevated iPTH/FGF23, and vascular calcification. Hypophosphatemia should also be avoided because of bone mineral needs.

7.1 We suggest that for children in CKD G2 to G5D to maintain serum calcium and phosphorus within the normal range of the corresponding age (not graded).

#### Rationale

Children with CKD are in a critical period of growth and development, requiring sufficient calcium to promote bone development. Children with CKD with insufficient calcium supply may experience poor bone mineralization, delayed growth and development, leading to short stature in adulthood.^67^, ^68^, ^69^, ^70^ However, excessive intake of calcium can increase the risk of ectopic calcification, which can easily lead to cardiovascular complications.^71^ Therefore, close attention should be paid to the serum calcium levels and vascular calcification in children with CKD, and calcium supplementation plans should be adjusted if necessary.

Hyperphosphatemia can increase the level of reactive oxygen species and free radicals in the body, causing oxidative damage and endothelial dysfunction, leading to abnormal vascular function. It can also indirectly cause an increase in the levels of iPTH and FGF23, and participate in the inhibition process of 1,25(OH)_2_ vitamin D synthesis, further leading to vascular calcification and the occurrence of cardiovascular adverse events.^72^^,^^73^ Therefore, controlling serum phosphorus levels is extremely important for the prevention and treatment of cardiovascular diseases. At the same time, it is important to consider the increasing demand for minerals in children’s rapidly growing bones. Therefore, it is necessary to avoid keeping serum phosphorus below normal levels.

The 2017 KDIGO guideline^17^ recommended that the serum calcium and phosphorus levels of children with CKD G2 to G5D should be maintained within the normal range for the corresponding age. However, because of the lack of standardized original research and systematic evaluation, evidence supporting a corresponding reference range was provided for children of different ages. The KDOQI guideline^25^ and the Japanese Society for Dialysis Therapy guideline^74^ have divided the recommended range of children's serum calcium and phosphorus according to different ages. However, the recommended range of the Japanese guideline for children of different age groups is complex and difficult to implement. Therefore, combined with clinical practice operability, fairness, guideline methodology, and report quality evaluation results, this guideline mainly refers to the 2009 KDOQI guideline. It is recommended to maintain serum calcium and phosphorus in children with CKD within the normal range for the corresponding age (Table 3).

**Table 3:** Reference range of serum calcium and phosphorus for children with CKD G2 to G5D at different ages

Parameter	0–5 mo	6–12 mo	1–5 yr	6–12 yr	13–20 yr
Total calcium (mg/dl)	8.7–11.3	8.7–11.0	9.4–10.8	9.4–10.3	8.8–10.2
Phosphorus (mg/dl)	5.2–8.4	5.0–7.8	4.5–6.5	3.6–5.8	2.3–4.5

CKD, chronic kidney disease.

Ca: 1 mg/dl = 0.25 mmol/l; P: 1 mg/dl = 0.3229 mmol/l.

### Clinical Question 8: What is the Recommended Range of Daily Intake of Dietary Calcium and Phosphorus for Children With CKD G3a to G5D?

#### Key Takeaways

• Calcium intake limit: Total daily intake (diet + phosphate binders) should not exceed twice the DRI for age, balancing bone needs with ectopic calcification risk.
• Phosphorus intake principles: Maintain intake within 100% of DRI while ensuring adequate protein. If serum phosphorus and iPTH are elevated, restrict intake to ≤ 80% of DRI.

8.1 We suggest that the total daily intake of elemental calcium (including phosphate binders) in children with CKD G2 to G5D should not exceed twice the DRI (not graded).
8.2 We suggest that children with CKD G3a to G5D should not exceed their daily DRI intake of phosphorus while ensuring protein intake. For children with elevated levels of serum phosphorus and iPTH, it is recommended that the intake of dietary phosphorus should not exceed 80% of DRI at the same age (not graded).

#### Rationale

Serum calcium levels in children with CKD may be affected by dietary intake, levels of activated vitamin D, and serum phosphorus. Children with advanced CKD may have inadequate calcium intake because of impaired absorption caused by vitamin D deficiency. It is recommended to encourage the consumption of foods with high endogenous calcium content and calcium-fortified foods, such as milk, yogurt, cheese, soy products, Chinese cabbage, broccoli, etc.^75^, ^76^, ^77^ When choosing foods with different calcium contents, it is necessary to pay attention to the calcium bioavailability of different foods. Compared with grains and most vegetables, calcium in dairy products has a higher bioavailability of up to 30%; foods with high phytate content, such as bran grains, may have lower bioavailability of calcium.^76^^,^^77^ In addition, it is also necessary to pay attention to other factors that affect calcium absorption. Lactose can improve the absorption rate of calcium in the body; plant components such as fiber, phytic acid, and oxalic acid may inhibit calcium absorption.^76^ When consuming various dairy products with high calcium bioavailability, such as milk, the phosphorus content in the food should also be taken into account, and serum phosphorus levels should be monitored at the same time.

The intake of phosphorus in the human body mainly comes from food, so it is generally believed that limiting dietary phosphorus intake can be the main treatment method for regulating serum phosphorus levels in children with CKD-MBD. Many foods not only have high levels of phosphorus (such as meat and fish, nuts, whole grains, beans, cheese, etc.), but also contain many other important nutrients. Therefore, strict control of these foods to limit phosphorus intake may lead to severe malnutrition. In addition, considering that the bioavailability of phosphorus in different types of food varies in the human body,^78^ there is currently no research-based medical evidence to support the goal of controlling serum phosphorus by controlling the type and proportion of food intake. Research has shown that higher dietary phosphorus intake in children with CKD G3 can cause an increase in iPTH levels and exacerbate hyperparathyroidism,^79^ and strict restrictions on dietary phosphorus intake may exacerbate osteomalacia in children with moderate to severe CKD.^80^ Therefore, it is necessary to fully consider the mutual influence of the 3 key indicators of serum phosphorus, calcium, and iPTH in the body of patients with CKD-MBD.^81^

There is currently a lack of relevant evidence on the upper limit of daily dietary calcium and phosphorus intake for children with CKD of different ages. Therefore, the final recommendations made in this guideline mainly refer to the 2009 KDOQI guideline^25^ and the 2019 Pediatric Renal Nutrition Taskforce guideline,^82^ and are based on expert opinions, combined with factors such as clinical feasibility and children's growth and development needs. This guideline recommends a safety limit of 200% for children with DRI, which means that the total oral and/or intestinal calcium intake from nutritional sources and phosphate binders should be within the range of 100% to 200% of the corresponding age-related calcium DRI. At the same time, this guideline recommends that the daily phosphorus intake of children with CKD G3a to G5D should not exceed the daily DRI of the same age group; children with increased levels of serum phosphorus and iPTH should control their dietary phosphorus intake to be lower than 80% of the same age DRI (Table 4). However, clinicians should be aware that serum calcium levels are tightly regulated and may not reflect true calcium balance. Future practice should move beyond relying solely on serum calcium, prioritizing the combination of dietary calcium estimates and urinary calcium monitoring instead to accurately assess net calcium balance. But in the future, more high-quality clinical trials are needed for further confirmation of these assessment strategies and intake thresholds.

**Table 4:** Phosphorus intake in children with CKD G2 to G5D at different ages (mg/d)

Serum PTH and phosphorus	0–6 mo	7–12 mo	1–3 yr	4–8 yr	9–18 yr
High PTH and Normal phosphorus	≤ 100	≤ 275	≤ 460	≤ 500	≤ 1250
High PTH and High phosphorus	≤ 80	≤ 220	≤ 370	≤ 400	≤ 1000

PTH, parathyroid hormone; CKD, chronic kidney disease.

### Clinical Question 9: What is the Appropriate Calcium Supplementation Strategy for Hypocalcemia in Children With CKD G3a to G5D?

#### Key Takeaways

• Indications for calcium supplementation: Dietary calcium intake below DRI requirements; presence of hypocalcemia
• Benefits: Essential for bone accrual and growth; hypocalcemia negatively impacts final adult height.
• Safety considerations: Monitor closely for hypercalcemia, especially when combined with active vitamin D

9.1 For the children with CKD G3a to G5D, when dietary intake is insufficient to meet the DRI requirements and/or when hypocalcemia is concomitantly present, the administration of calcium supplementation is recommended (1C; see Supplementary Tables S26–S30).

#### Rationale

Because of the necessity for growth and development, the entire pediatric period represents a critical phase of bone mass accrual. Calcium supplementation can increase bone cortical density, thereby reducing the risk of fractures in pediatric patients with CKD.

In 2005, a prospective clinical trial conducted in India included a total of 100 pediatric patients with idiopathic nephrotic syndrome who were being treated with corticosteroids and had normal kidney function (GFR > 90 ml/min per 1.73 m^2^). These patients were subjected to combined therapy with calcium (500 mg/day) and vitamin D3 (200 IU/day) for a follow-up period of 1.5 ± 0.07 years. The results revealed a significant increase in the serum calcium levels of the patients after treatment compared with pretherapy (8.7 ± 0.07 mEq/l vs. 8.5 ± 0.10 mEq/l, *P* = 0.007). The average lumbar spine BMD value significantly improved compared with the baseline (0.607 ± 0.013 g/cm^2^ vs. 0.561 ± 0.01 g/cm^2^, *P* < 0.0001). A comparison was made between 15 patients who discontinued treatment at the beginning (Group I) and 73 patients who continued treatment (Group II). The BMD ΔZ score in Group II patients showed significant improvement (−0.382 ± 0.116 vs. 0.298 ± 0.111, *P* = 0.008). These findings suggest that calcium supplementation can effectively improve pediatric hypocalcemia and increase BMD in children.^83^

In 2011, an RCT conducted in the United States included 110 nondialysis CKD G4 to G5 adult patients with concomitant hyperphosphatemia (serum phosphate > 4.5 mg/dl). These patients were randomly assigned to receive either calcium acetate (*n* = 46) or placebo (*n* = 64) and were followed for 12 weeks. The results demonstrated that the serum calcium concentration in the calcium acetate group was significantly higher than that in the placebo group (9.5 ± 0.8 vs. 8.8 ± 0.8 pg/ml, *P* < 0.001), whereas the serum phosphate concentration was significantly lower in the calcium acetate group compared with the placebo group (4.4 ± 1.2 vs. 5.1 ± 1.4 mg/dl, *P* = 0.04). The incidence of hypocalcemia in the calcium acetate and the placebo groups was 5.4% and 19.5%, respectively.^84^ In 2002, an RCT study in the United States included nondialysis male patients with a creatinine clearance (Ccr) ranging from 10 to 70 ml/min per 1.73 m^2^ and randomized them into a low-dose group (n = 8, oral elemental calcium 507 mg/day) and a high-dose group (*n* = 10, oral elemental calcium 1521 mg/day), with a 24-week follow-up. The results indicated that the high-dose group of patients had a baseline average serum calcium concentration of 9.2 ± 0.2 mg/dl, which peaked with a 7.2% increase in mean serum calcium concentration at week 16 (9.8 ± 0.3 mg/dl), whereas serum phosphate concentration remained unchanged. Following treatment, there was a significant increase in BMD in the first lumbar vertebra (*P* = 0.02), third lumbar vertebra (*P* = 0.05), and fourth lumbar vertebra (*P* = 0.03) for these patients. In contrast, the changes in serum calcium, serum phosphate levels, and BMD for patients in the low-dose group were not statistically significant (*P* > 0.05).^85^ The above-mentioned studies suggest that calcium supplementation can raise serum calcium levels, lower serum phosphate levels, and improve BMD in patients with CKD (Supplementary Tables S26–S30).

KDIGO guidelines recommend that for adult patients with CKD G3a to G5D, hypercalcemia should be avoided, and hypocalcemia in adults should be individually addressed rather than advocating for the correction of hypocalcemia in all patients. However, for pediatric patients with CKD, the guidelines suggest maintaining serum calcium levels within the age-appropriate normal range.^17^ It is very important to avoid hypocalcemia in children, as low serum calcium may result in poor bone mineral accrual, frequent fractures, and growth retardation, resulting in short stature in adulthood. The recommendation is primarily based on direct pediatric evidence and high-quality adult evidence. Pediatric evidence includes a prospective study in children with nephrotic syndrome, which showed that calcium supplementation combined with vitamin D significantly increased serum calcium levels and improved lumbar spine BMD.^83^ However, the study population had normal kidney function and was not specific to CKD-MBD, limiting the extrapolation of findings. Additionally, 2 adult RCTs demonstrated that calcium supplementation effectively corrects hypocalcemia, lowers serum phosphorus, and improves BMD, providing indirect support for pediatric recommendations.^84^^,^^85^ High-quality adult evidence has clearly established the efficacy and safety of calcium supplementation in patients with CKD. However, it is worth noting that the risk of hypercalcemia is greatly increased when calcium and active vitamin D are combined.^86^ Therefore, the serum calcium during treatment should be closely monitored to avoid the occurrence of hypercalcemia. At present, there are few studies on the correlation between CKD-MBD and calcium supplementation in children, and the conclusions based on the existing evidence may have certain risks in reliability. It is expected that more relevant studies in children will be conducted in the future.

### Clinical Question 10: How to Choose Phosphate-lowering Drugs for Children With CKD G3a to 5D Who Develop Hyperphosphatemia?

#### Key Takeaways

• First-line therapy (if serum calcium < upper limit of normal [ULN]): Use calcium-based phosphate binders, alone or combined with noncalcium metal-based binders; nonmetal-based binders
• Restrict calcium-based binders in children with persistent hypercalcemia, arterial calcification, low iPTH, or adynamic bone disease
• Efficacy: All binders effectively lower phosphorus, but with the following trade-offs:
(ⅰ) Calcium-based: Low cost, corrects hypocalcemia, but increase hypercalcemia risk
(ii) Noncalcium-based: Fewer cardiovascular events (sevelamer), but higher cost and gastrointestinal side effects

10.1 For the children with CKD G3a to G5D and serum calcium levels below the age-specific normal upper limit, it is recommended to use calcium-based phosphate binders (1B) and/or combined with noncalcium metal-based phosphate binders (1B), nonmetal-based phosphate binders (1A) for the treatment of hyperphosphatemia (see Supplementary Tables S31–S35).
10.2 If the child has comorbidities such as persistent hypercalcemia or arterial calcification, low iPTH levels, or dystrophic bone disease, the use of calcium-based phosphate binders should be restricted (1B; see Supplementary Tables S31–S35).

#### Rationale

When CKD progresses to the later stages, because of the calcium-phosphorus metabolism disorders, it often leads to persistent hyperphosphatemia that is difficult to alleviate through dietary control. When the serum phosphorus levels in children with CKD remain consistently higher than the upper limit of the reference range for their age, phosphate binders and dialysis can be used to lower the phosphorus levels. Commonly used phosphate binders in clinical practice include calcium-based phosphate binders and noncalcium-based phosphate binders, which can be further classified into metal-based and nonmetal-based phosphate binders.

A Cochrane systematic review conducted in 2011 included 60 studies (*n* = 7631) and compared the efficacy of different phosphate binders in adult patients with CKD G3 to G5D. The results showed that compared with sevelamer, calcium-based phosphate binders had significant advantages in lowering serum phosphorus levels (16 studies, *n* = 3126; mean deviation [MD]= 0.23 mg/dl, 95% CI: 0.04–0.42) and controlling iPTH levels (12 studies, *n* = 2551; [MD] = 56 pg/ml, 95% CI: 26–84). However, they were more likely to cause hypercalcemia (12 studies, *n* = 1144; risk ration = 0.45, 95% CI: 0.35–0.59).^87^ In a crossover RCT conducted in Japan in 2012, 50 patients with ESRD were included to compare the effects of calcium carbonate and lanthanum carbonate (LC) in lowering serum phosphorus levels. The results showed that compared with baseline, the serum phosphorus levels were significantly decreased in both the calcium carbonate group (7.30 ± 1.86 vs. 5.6 ± 1.2 mg/dl) and the LC group (7.76 ± 2.14 vs. 5.6 ± 1.3 mg/dl) after 3 months of treatment (both *P* < 0.05); during the treatment period, 2 cases of hypercalcemia were reported in the calcium carbonate group, whereas none occurred in the LC group.^88^ The above evidence suggests that calcium-based phosphate binders are effective in lowering phosphorus levels in patients with CKD, but the risk of hypercalcemia should be noted.

Metal-based phosphate binders mainly include LC, iron-based phosphate binders, and aluminum-based phosphate binders. In a phase II randomized, double-blind, placebo-controlled trial conducted in the United States in 2009, a total of 121 patients with CKD G3 to G4 were included (80 in the LC group and 41 in the placebo group). The results showed a significant reduction in serum phosphorus levels in the LC group compared with the placebo group (0.55 ± 0.10 vs. 0.18 ± 0.13 mg/dl, *P* = 0.02).^89^ In addition to LC, iron-based phosphate binders are also widely used in clinical practice. A meta-analysis conducted in 2015 (including 8 studies, *n* = 2018) investigated the efficacy and safety of iron-based phosphate binders in adult dialysis patients. The results showed a significant reduction in serum phosphorus levels with iron-based phosphate binders (MD = −2.43 mg/dl, 95% CI: −3.18 to −1.68, *P* < 0.01), comparable to sevelamer (MD = 0.04 mg/dl, 95% CI: −0.29 to 0.36, *P* = 0.83). Additionally, iron-based phosphate binders could also moderately increase serum iron levels (MD = 9.39 ng/ml, 95% CI: 1.48–17.30, *P* = 0.02), thereby improving renal anemia.^90^ The above research results indicate that both LC and iron-based phosphate binders can effectively lower serum phosphorus levels in patients with CKD. However, aluminum-based binders have largely been phased out of long-term clinical use because of their skeletal and central nervous system toxicity.

Nonmetal-based phosphate binders are mainly represented by sevelamer. In 2015, a Cochrane systematic review included 3 RCTs to compare the efficacy of sevelamer with calcium-based phosphate binders. The results showed no significant differences in the mean serum phosphorus levels (0.17 mg/dl, 95% CI: 0.37–0.71, I^2^ = 0%), mean calcium-phosphorus product (−1.12 mg/dl, 95% CI: −5.88 to 3.64, I^2^ = 0%), and mean serum calcium levels (−0.40 mg/dl, 95% CI: −1.16 to 0.36, I^2^=59%) between the 2 groups.^91^ In 2008, a prospective RCT in Turkey included 50 hyperphosphatemia patients with CKD stage 4. After an 8-week follow-up, it was found that the sevelamer group had a significant decrease in serum phosphorus levels (7.8 ± 0.6 vs. 5.9 ± 0.9, *P* < 0.01). The levels of fetuin-A and blood flow-mediated dilation index were significantly improved, which helped reduce cardiovascular events in patients.^92^ This evidence suggests that sevelamer can effectively lower serum phosphorus levels and may reduce the incidence of cardiovascular events. Except for sevelamer, nonmetal-based phosphate binders also include colestilan, tenapanor, and nicotinamide. Colestilan, a nonabsorbed anion exchange resin that promotes intestinal phosphorus excretion, is used as a phosphorus-reducing drug in CKD dialysis patients. It also binds bile acids and reduces serum levels of low-density lipoprotein cholesterol. In a multicenter RCT study, the efficacy of colestilan was investigated in 642 patients with CKD G5 dialysis with both hyperphosphatemia and dyslipidemia. The results showed that colestilan could significantly lower serum phosphorus levels and improve dyslipidemia, with good tolerance, confirming that colestilan is an effective drug for treating hyperphosphatemia in patients with CKD.^93^ Tenapanor, a local inhibitor of sodium/hydrogen exchanger isoform 3, acts as a phosphate absorption inhibitor by decreasing paracellular phosphate absorption. Whether used alone or in combination with other phosphate binders, tenapanor can significantly reduce serum phosphorus levels, especially in combination therapy, where tenapanor has better efficacy in reducing blood phosphorus.^94^, ^95^, ^96^ Nicotinamide has been reported as an adjunctive therapy for hyperphosphatasemia in patients with CKD. It can not only reduce the phosphorus and FGF23 levels but also slow down the descending rate of Klotho in chronic HD patients (Supplementary Tables S31–S35).^97^

The recommendations are derived primarily from high-quality adult evidence, with limited direct pediatric studies. The adult evidence includes multiple meta-analyses and RCTs demonstrating that both calcium-based phosphate binders and noncalcium-based phosphate binders can effectively lower serum phosphorus levels, but each type of phosphate-lowering agent has its advantages and disadvantages. Calcium-based phosphate binders can lower serum phosphate levels while also improving hypocalcemia, with lower cost and better tolerability, but they may increase the risk of hypercalcemia. Metal-based phosphate binders have good tolerability, minimal systemic absorption, and can help promote gastrointestinal function, but they are relatively expensive. Nonmetal-based phosphate binders have minimal systemic absorption and a low incidence of cardiovascular events, but they have high costs, poor tolerability, and significant gastrointestinal side effects. For children with CKD G3a to G5D who are growing and developing, calcium-based phosphate binders can lower serum phosphorus levels, correct calcium deficiency, and improve growth delay, making it the primary choice for managing hyperphosphatemia in CKD-MBD patients. Noncalcium-based phosphate binders need to be selected or adjusted based on individual patient conditions. If the patient has persistent hypercalcemia, arterial calcification, low iPTH levels, or dynamic bone disease, the use of calcium-based phosphate binders should be restricted.

### Clinical Question 11: How to Adjust the Dialysis Regimen for Hypocalcemia or Hyperphosphatemia in Children With CKD G5D?

#### Key Takeaways

• Hypocalcemia (ionized Ca < 1.25 mmol/l): Consider individualized adjustment of dialysate calcium. Monitor serum calcium closely.
• Persistent hyperphosphatemia: May use intensified dialysis to enhance phosphate removal (2D).
• Intensified dialysis benefits: Lowers serum phosphorus and PTH, reduces phosphate binder needs, and may improve growth.
• Challenges: Higher cost, longer treatment time, limited access, and need for more pediatric evidence.

11.1 In children with CKD G5D, when the ionized calcium concentration falls below 1.25 mmol/l, individualized adjustments to the calcium-containing dialysate prescription should be considered. Close monitoring of serum calcium levels during treatment is advisable (not graded).
11.2 For the children with persistent hyperphosphatemia in CKD G5D, implementing an intensified dialysis regimen may be considered to enhance the removal of phosphate from the blood (2D; see Supplementary Tables S36–S39).

#### Rationale

The calcium concentration in dialysate can influence both the serum calcium concentration in the body and calcium balance during the dialysis process.^98^^,^^99^ Multiple studies in adults have shown that patients receiving low-calcium dialysate (1.25 mmol/l) have lower serum calcium levels and experience a more rapid decline in bone density when compared with those in the high-calcium dialysate group (1.50–1.75 mmol/l).^100^^,^^101^ Therefore, when formulating dialysis regimens for pediatric patients with CKD, the impact of dialysate calcium concentration on both systemic serum calcium levels and bone density should be taken into consideration.

In 2003, the KDOQI guidelines recommended the use of dialysate containing 1.25mmol/l of calcium for pediatric CKD G5D patients. These guidelines suggested that with the use of calcium-based phosphate binders and active vitamin D analogs, the most appropriate dialysate calcium concentration is 1.25 mmol/l.^102^ However, in the 2017 updated KDIGO guidelines, it was advised that the range of dialysate calcium concentration should fall between 1.25 and 1.50 mmol/l (2.50–3.00 mEq/l), and the evidence grade was upgraded from 2D to 2C based on new adult studies.^17^ Actual calcium balance studies are challenging to conduct, and there is a lack of related literature. Before the introduction of calcium-based phosphate binders, adults typically used dialysate containing 1.5–-1.75 mmol/l of calcium during dialysis to prevent calcium loss. However, with the widespread use of calcium-based phosphate binders, there has been a gradual shift towards the use of dialysate containing 1.25 mmol/l of calcium.^103^ Calcium supplementation offers a broader scope for adjustment to meet the varying calcium needs of different individuals.

The mechanism of bone and mineral metabolism in pediatric patients with CKD is complex, and calcium requirements vary among different age groups. Currently, there is no standardized guideline for selecting dialysate calcium concentration in children, emphasizing the need for individualized adjustments. As children are in a period of rapid growth and development, they require a positive calcium balance.^104^ Therefore, the choice of dialysate calcium concentration for CKD pediatric patients should aim to meet the mineral needs of growing bones while considering various influencing factors, including individual requirements, dialysis modality, dietary intake, medications, and others. Currently, there is a lack of research evidence regarding the adjustment of dialysate calcium concentration for pediatric CKD G5D patients with hypocalcemia, and the selection of dialysate calcium concentration in children continues to rely on clinical experience and individualized adjustments.

When drug therapy or conventional HD fails to effectively lower elevated serum phosphorus levels in pediatric CKD G5 patients with hyperphosphatemia, an intensified dialysis regimen may be attempted to reduce phosphorus. Currently, there is no internationally recognized intensified dialysis regimen. The primary approaches to intensified dialysis include short daily dialysis (2–3 hours/day, 5–7 days/week),^105^^,^^106^ nocturnal intermittent dialysis (8 hours/day, 3 days/week),^107^^,^^108^ and daily nocturnal dialysis (6–8 hours/day, 5–7 days/week).^109^^,^^110^

In a prospective cohort study conducted in Germany in 2011, 16 pediatric dialysis patients were included. The results showed that compared with conventional HD, children who underwent intensified dialysis (nocturnal intermittent dialysis, 3 times per week, 8 hours per session) had a significant decrease in mean serum phosphorus levels (2.14 vs. 1.37 mmol/l) and PTH levels (445 vs. 184 ng/l). Additionally, the average Kt/V increased (1.74 vs. 2.15) after intensified dialysis. The dosage of phosphate binders was reduced, and in some cases, discontinued altogether.^111^ In a single-center prospective cohort study in France in 2004, a total of 5 CKD pediatric patients who had received standard HD for at least 6 months (3 times per week, 4 hours per session) were included. The results demonstrated that after transitioning to intensified dialysis (6 times per week, 3 hours per session), the serum phosphorus levels in the children showed a significant decrease at baseline, 6 months, and 12 months, measuring 1.87 ± 0.23 mmol/l, 1.43 ± 0.22 mmol/l, and 1.28 ± 0.29 mmol/l, respectively (*P* < 0.05). The calcium-phosphorus product levels (Ca × P) also significantly decreased from the baseline value of 4.3 ± 1.8mmol^2^/l^2^ to 3.7 ± 0.8mmol^2^/l^2^ at 6 months (*P* < 0.05) and 3.6 ± 0.4mmol^2^/l^2^ at 12 months (*P* < 0.05). One patient experienced catch-up growth 6 months after the change in dialysis regimen (Supplementary Tables S36–S39).^112^

For intensified dialysis, pediatric evidence includes 2 small cohort studies demonstrating that intensive regimens (nocturnal or daily dialysis) significantly lower serum phosphorus and PTH, reduce phosphate binder requirements, and may improve growth.^111^^,^^112^ However, these studies are limited by small sample sizes, observational design, and lack of control groups. Moreover, intensified dialysis is associated with higher costs, longer treatment times, challenges in the long-term maintenance of arteriovenous fistulas, and limited accessibility, which may hinder widespread adoption. Large-sample, long-term follow-up RCTs in the pediatric population are still lacking, and the reliability of existing conclusions requires further validation.

### Clinical Question 12: What is the Recommended Range of Serum Vitamin D Concentration for Children With CKD G2 to G5D?

#### Key Takeaways

• Target: Maintain serum 25(OH)D > 30 ng/ml (>75 nmol/l) in all children with CKD G2 to G5D.
• Supplementation: If below target, give natural vitamin D (D3 preferred), with regimen based on age and deficiency severity.
• Benefits: Prevents rickets, delays ROD, supports calcium-phosphorus balance.

12.1 We recommend that the serum 25(OH)D concentration for the children with CKD G2 to G5D should be maintained above 30 ng/ml (> 75 nmol/l) (not graded).
12.2 We recommend supplementing natural vitamin D for children with CKD G2 to G5D who have serum 25(OH)D levels below 30 ng/ml, and selecting the appropriate regimen based on age and severity of deficiency (not graded).

#### Rationale

Children with CKD are more prone to develop vitamin D deficiency because of calcium and phosphorus metabolism disorders, leading to an increase in the incidence of rickets compared with normal children. Therefore, it is crucial to maintain serum 25(OH)D concentration above normal levels for children with CKD G2 to G5D.

Guidelines published by the European Society for Pediatric Nephrology CKD-MBD Working Group in 2017 stated that when the serum 25(OH)D level in children with CKD is below 30 ng/ml, it indicates vitamin D insufficiency; when the level is between 5 to 20 ng/ml, it indicates vitamin D deficiency; and when it is below 5ng/ml, it indicates severe vitamin D deficiency.^113^ The 2011 guidelines from the American Society of Endocrinology also recommend maintaining serum 25(OH)D levels above 30 ng/ml in children to prevent nutritional rickets and maintain optimal intestinal calcium absorption status.^114^ The 2009 KDOQI Nutrition Guidelines^25^ suggest that when the serum 25(OH)D level in children with CKD is below 30 ng/ml, supplementation with natural vitamin D should be provided. Based on the recommendations of existing guidelines, this guideline recommends maintaining serum 25(OH)D concentration above 30 ng/ml (> 75 nmol/l) for children with CKD G2 to G5D to reduce the risk of nutritional rickets and delay the progression of ROD.

For patients with CKD with 25(OH)D deficiency, vitamin D supplementation can help maintain serum calcium and phosphorus levels within the target range, improve bone disease, and delay the progression to SHPT without significant side effects such as hypercalcemia.^115^ The overall benefit is greater than the risk, and the intervention cost is low; even in areas with poor medical conditions, the supplementation can also be implemented well and has high feasibility. Therefore, based on the quality evaluation results of the guidelines mentioned above and the feasibility of the recommended regimen, this guideline mainly refers to the European Society for Pediatric Nephrology guidelines from 2017 to make recommendations. For the children with CKD G2 to G5D with serum 25(OH)D levels below 30 ng/ml, individualized vitamin D supplementation (including loading and maintenance regimens) should be applied according to the different ages and severity of deficiency (Table 5). Vitamin D3 (cholecalciferol) supplementation is recommended as a priority because it is more effective in raising serum 25(OH)D levels, both in healthy people and in patients with CKD undergoing HD.^116^^,^^117^ After initiating vitamin D supplementation, regular blood biochemistry tests should be performed to maintain serum 25(OH)D levels within the target range and reduce the risk of complications.

**Table 5:** Supplemental doses of vitamin D for different age groups with vitamin D deficiency in CKD G2 to G5D

Age	Loading regimens			Maintenance regimens
	< 12 nmol/l	12–50 nmo/l	50–75 nmol/l	
< 1 yr	600 IU/d	600 IU/d	600 IU/d	400 IU/d
> 1 yr	8000 IU/d	4000 IU/d	2000 IU/d	1000–2000 IU/d based on CKD stage

CKD, chronic kidney disease.

### Clinical Question 13: What is the Recommended Range for iPTH Levels in Children With CKD G3a to G5D?

#### Key Takeaways

• CKD G2–G3: iPTH within normal range
• CKD G4: iPTH < 2 × ULN
• CKD G5–G5D: iPTH 2–9 × ULN
• Based on expert consensus; aims to balance bone health and avoid a dynamic bone disease

13.1 We suggest for children with CKD G2 to G3, it is recommended to control iPTH levels within the normal range. For children with CKD G4, iPTH levels should be controlled below twice the upper limit of the normal range. For children with CKD G5 to G5D, it is recommended to maintain iPTH levels between 2 to 9 times the upper limit of the normal range (not graded).

#### Rationale

There is no consensus on the recommended range of iPTH levels in children with CKD, but the existing guidelines agree that the recommended range in children with CKD G2 to G3 is to maintain them within the ULN range.^21^^,^^25^ However, as renal function impairment progresses, for children with CKD G4, the 2009 KDOQI guideline suggests that iPTH can be maintained between 70–110 pg/ml,^25^ whereas the 2013 Japanese Society for Dialysis Therapy guideline recommends controlling it below 1.5 times the ULN.^74^ For children with CKD G5, the European pediatric guideline of European Pediatric Dialysis Working Group suggests maintaining PTH levels between 2 and 3 times the ULN,^21^ whereas the 2009 KDOQI guideline recommends a target range of 200–300 pg/ml for iPTH in children with CKD G5.^25^ The 2017 KDIGO guideline recommends a target range of 2 to 9 times the ULN (120–540 pg/ml) for iPTH,^17^ whereas the Japanese Society for Dialysis Therapy guideline suggests controlling iPTH levels within a range of 1.5 to 4.5 times the ULN.^74^ It is worth mentioning that these recommendations are mainly based on expert consensus and are not supported by high-quality evidence.

Therefore, considering expert consensus, clinical practice experience, and feasibility, this guideline recommends maintaining iPTH levels within the normal range for children with CKD G2 to G3, controlling iPTH levels below twice the normal value for children with CKD G4, and referring to the 2017 KDIGO guideline, recommending that children with CKD G5 to G5D maintain iPTH levels between 2 and 9 times the ULN. This approach aims to achieve reasonable biological function, improve the quality of life for children, reduce the incidence of bone disease, and decrease mortality rates.

### Clinical Question 14: How to Supplement Active Vitamin D and Vitamin D Analogues in the Children With CKD G3a to G5D When They Have SHPT?

#### Key Takeaways

• Severe/progressive SHPT: Start oral calcitriol. Initial dose: 10–20 ng/kg/d, daily or intermittent.
• Mild SHPT or stable iPTH: Use vitamin D analogues—alfacalcidol or paricalcitol.
• Efficacy: All agents effectively lower iPTH; calcitriol and analogues improve bone metabolism.
• Safety: Monitor calcium, phosphorus, iPTH closely; paricalcitol may have lower hypercalcemia risk.
• Dosing principle: Start low, individualize based on response and SHPT severity.

14.1 For children with CKD G3 to G5D who have severe and progressive SHPT, we recommend starting initial oral calcitriol therapy to reduce excessively high iPTH levels (1B; see Supplementary Tables S40–S44).
14.2 When the total dose of weekly oral calcitriol is consistent, oral calcitriol can be administered daily or intermittently, with an initial dose of 10–20 ng/kg/d, to control iPTH levels within a reasonable range (1B; see Supplementary Tables S40–S44).
14.3 For the children with mild SHPT or stable iPTH levels in CKD G3 to G5D, we suggest oral treatment with vitamin D analogues. For patients with CKD, we recommend oral administration of alfacalcidol (1C) and paricalcitol (1B) to control iPTH levels (see Supplementary Tables S45–S49).

#### Rationale

Increased iPTH is common in children with CKD, and its secretion is influenced by multiple factors such as decreased calcitriol, decreased serum calcium, and increased phosphate levels. Research has found that more than 50% of children with CKD have elevated iPTH levels.^118^ As estimated glomerular filtration rate decreases, FGF23 level increases and inhibits the 1α-hydroxylase enzyme in the proximal tubular epithelium, resulting in decreased calcitriol production and ultimately leading to SHPT.^119^ In clinical practice, active vitamin D and vitamin D analogues are both used in the treatment of SHPT in pediatric patients with CKD. They could increase the plasma levels of active vitamin D and improve bone and mineral metabolism abnormalities in patients with CKD.

Currently, active vitamin D, mainly calcitriol, is used widely in the treatment of patients with CKD. In 2005, a multicenter RCT study included 47 pediatric ESRD patients from the United States and Poland, divided into the calcitriol group and placebo group. After 12 weeks of treatment, 52% of patients in the calcitriol group had a 30% decrease in PTH levels compared with baseline, whereas only 19% in the placebo group (*P* = 0.03).^120^ In a 2003 European RCT study, 24 children with CKD with Ccr < 40 ml/min per 1.73 m^2^ were randomly divided into the daily oral group (10 ng/kg/d) and the intermittent oral group (twice a week, 35 ng/kg/dose). After 1 year of treatment, iPTH levels decreased from 567 pg/ml (114–1209) to 255 pg/ml (85–710) in the daily oral group, whereas they decreased from 332 pg/ml (93–614) to 179 pg/ml (51–443) in the intermittent oral group. There was no statistically significant difference in iPTH levels and growth rate between the 2 groups after treatment.^121^ In a 2000 European RCT study, 59 children with CKD with Ccr < 75 ml/min per 1.73 m^2^ were treated with calcitriol, divided into a daily oral group (*n* = 29, 10 ng/kg/d) and intermittent oral group (n = 30, twice a week, 35 ng/kg/dose). After 8 weeks, the median iPTH in the daily oral group decreased from 485 pg/ml (83–2032) to 232 pg/ml (63–1614), and in the intermittent oral group, it decreased from 315 pg/ml (93–1638) to 218 pg/ml (2–1785). The average decrease in iPTH were 19.2 ± 57.8% versus 13.7 ± 46.7%, respectively, with no statistical difference between the groups (Supplementary Tables S40–S44).^122^

Except for active vitamin D, the vitamin D analogues mainly used in the clinical treatment of SHPT in children with CKD are alfacalcidol (the former being a nonselective vitamin D analogue) and paricalcitol (the latter being a selective vitamin D receptor agonist).^123^ Vitamin D analogues could provide cardiovascular protection for patients with concurrent SHPT and reduce mortality.^124^, ^125^, ^126^, ^127^

A prospective case series study in Finland in 1995 included 22 children with CKD with concurrent SHPT (mean Ccr: 20 ± 3 ml/min per 1.73 m^2^), who were treated with oral alfacalcidol (0.5–3.0 μg/dose, 3 times a week) and followed up for 12 months. The results showed that the children’s iPTH levels significantly decreased compared with baseline (398 ± 81 vs. 122 ± 34 ng/l).^128^ There was no significant change in Ccr after treatment, and no occurrence of hypercalcemia events. A prospective case series study in Japan in 1990 included 12 children with continuous ambulatory peritoneal dialysis aged between 2 and 16 years, who were treated with oral alfacalcidol (0.01–0.02 μg/kg) for 12 to 18 months. The results showed that the PTH levels of the children significantly decreased after treatment (3.3 ± 2.4 vs. 1.3 ± 1.4, *P* < 0.01), and the height standard deviation score of 10 children increased or remained stable during the study period.^129^ The above evidence suggests that the use of alfacalcidol can help reduce serum iPTH levels in children with CKD with concurrent SHPT.

A systematic review of the application of paricalcitol in children with CKD in 2020 included a total of 103 children with CKD G3 to G5 (intervention group *n* = 53, control group *n* = 50). The intervention group was treated with oral paricalcitol (0.04 μg/kg/d) or intravenous infusion (3 times a week, 0.04–0.1 μg/kg per time). The results showed that paricalcitol could effectively lower iPTH levels in children with CKD (odds ratio [OR] = 0.12; 95% CI: 0.05–0.29; *P* < 0.001), and had a small effect on changes in serum calcium (OR = 1.16; 95% CI: 0.48–2.80; *P* = 0.741) and phosphorus (OR = 0.87; 95% CI: 0.38–1.99; *P* = 0.735), suggesting that paricalcitol can significantly reduce iPTH levels in children with CKD with a small effect on serum calcium concentration (Supplementary Tables S45–S49).^130^

To reduce the risk of extra-osseous calcification and cardiovascular events, guidelines from KDIGO and Japanese Society for Dialysis Therapy both recommend using the active vitamin D or vitamin D analogues for treatment in children with CKD G3a to G5 with SHPT.^8^^,^^71^ The recommendations in this guideline are primarily derived from direct pediatric evidence, supplemented by adult data. Pediatric evidence includes 3 RCTs on calcitriol demonstrating that oral calcitriol effectively reduces iPTH in children with SHPT, with no significant difference in efficacy between daily and intermittent dosing when the weekly total dose is consistent.^120^, ^121^, ^122^ And intravenous and intraperitoneal administration of calcitriol have been reported to be similar to oral administration in terms of absorption and reduction in iPTH levels.^131^, ^132^ For vitamin D analogues, 2 prospective case series showed alfacalcidol significantly lowers iPTH,^128^^,^^129^ and a 2020 systematic review confirmed paricalcitol effectively reduces iPTH with minimal impact on serum calcium and phosphorus.^130^ However, the pediatric evidence has the following limitations: small sample sizes, heterogeneous populations, variable follow-up durations, and lack of long-term safety data, particularly for newer analogues like paricalcitol. High-quality adult evidence has established that active vitamin D and its analogues effectively suppress iPTH and improve bone metabolism in patients with CKD, with comparative efficacy and safety profiles.

In summary, the evidence suggests that active vitamin D and vitamin D analogues can effectively reduce iPTH levels in children with CKD and can also improve MBD. The initial dose of oral calcitriol is 10–20 ng/kg/d, aiming to control iPTH within a reasonable range. The recommended dose for alfacalcidol in clinical practice is 0.5 μg/day for children. Paricalcitol is safer because it has a lower risk of persistent hypercalcemia and Ca x P deposition.^133^ But the application experience of paricalcitol in pediatric kidney disease is still very limited. Oral administration is more acceptable to children than intravenous infusion and is more feasible. The recommended initial dosage of paricalcitol in children with CKD is 0.04 μg/kg/d.^130^ The dose of vitamin D and its analogues should follow the principle of starting from low to high and individualized adjustment. During treatment with vitamin D and its analogues, close monitoring of serum calcium, phosphorus, and iPTH is needed, followed by individualized adjustments based on the severity of SHPT and the level of iPTH reduction to achieve a suitable physiological balance.

### Clinical Question 15: How to Supplement Calcimimetics in the Children With CKD G3a to G5D When They Have SHPT?

#### Key Takeaways

• Indication: CKD G4 to G5D with persistent iPTH > 300 pg/ml.
• Regimen: Combine cinacalcet with calcitriol or vitamin D analogues.
• Dosing: Start low (≤ 0.20 mg/kg/d), titrate to response, max 60 mg/d.
• Efficacy: Cinacalcet effectively lowers iPTH in children, may delay parathyroidectomy.
• Safety: Monitor serum calcium closely (risk of hypocalcemia); discontinue if symptoms occur.
• Evidence gap: Limited pediatric data; more pediatric RCTs needed.

15.1 The combination of cinacalcet, calcitriol, or vitamin D analogues could be considered for children with CKD G4 to G5D who have persistent iPTH levels above 300pg/ml in order to effectively control iPTH within the target range (2C; see Supplementary Tables S45–S49).
15.2 The treatment for children with CKD G4 to G5D and persistently elevated iPTH levels (> 300 pg/ml) could initiate cinacalcet therapy at a low dose (≤ 0.20 mg/kg/d), with subsequent dosage adjustments based on treatment efficacy, up to a maximum daily dose of 60 mg (2C; see Supplementary Tables S45–S49).

#### Rationale

Patients with CKD complicated by SHPT often have persistently high levels of iPTH, which can have serious clinical consequences if not treated appropriately.^134^ Calcimimetic is an allosteric agonist that acts on calcium-sensing receptors and can inhibit the release of iPTH and improve metabolic dysfunction in patients with CKD.^135^ Currently, there are 3 calcimimetic agents approved in the world, namely cinacalcet, etecaltide, and evocalcet. Cinacalcet is the most widely used clinically in children with CKD.

A randomized, double-blind, placebo-controlled phase III study from the US and Europe in 2019 enrolled 43 children with HD/peritoneal dialysis with a mean age of 13.2 years, who were divided into the cinacalcet group (*n* = 22, mean starting dose 0.18 mg/kg/day, maximum dose 60 mg/day) and the placebo group (*n* = 21). After 14 months of treatment, mean iPTH decreased by ≥ 30% from baseline in 54.5% of cinacalcet (*n* = 12) and 19% of placebo (*n* = 4) patients (*P* = 0.017).^136^ In a prospective cohort study in Saudi Arabia in 2015, 28 children with CKD G4 to G5 complicated by SHPT were treated with cinacalcet (starting dose 0.5 mg/kg/day and maximum dose 1.5 mg/kg/day) and followed up for 3 to 24 months. The results showed that iPTH levels decreased by more than 60% from baseline in all children (60%–97%), and mean serum iPTH levels decreased significantly after treatment (1931.76 ± 794.62 pg/ml vs. 354.25 ± 274.15 pg/ml, *P* < 0.001). There was no significant difference in mean serum calcium levels before and after treatment (2.45 ± 0.07 vs. 2.41 ± 0.13 mmol/l, *P* = 0.157), and no symptomatic hypocalcemic events occurred during treatment.^137^ In a 2019 retrospective US case series of 18 children < 5 years of age on dialysis (iPTH > 300 pg/ml), all patients had a significant reduction in mean iPTH levels from baseline after 6 months of treatment with cinacalcet (929 pg/ml [IQR 572–1056] vs. 385 pg/ml [IQR 140–710], *P* < 0.01), with a reduction of 51% (IQR 19–89).^138^ The above evidence suggests that cinacalcet can effectively reduce iPTH levels in children with CKD and SHPT to achieve treatment goals, but the occurrence of hypocalcemia should be monitored (Supplementary Tables S50–S54).

There are a few clinical studies of etelcalcetide use in children. A phase 1 study in 2021 evaluated the safety, tolerability, pharmacokinetics, and pharmacodynamics of single-dose etelcalcetide in pediatric HD patients (cohort 1: *n* = 6, 12–18 years; cohort 2: n = 5, 2–12 years). The results showed that the median percent change in serum iPTH from baseline reached the nadir on D1 at 4 hours (cohort 1: −33.4%; cohort 2: −64.2%). Two patients (1 per cohort) reported treatment-related AEs (cohort 1: hypocalcemia; cohort 2: headache, paresthesia, vomiting), no serious adverse events or deaths were reported.^139^ The etecalcetide is more commonly used in adults. A 2020 Italian network meta-analysis included 24 clinical trials of calcimimetics (*n* = 6521). The goal of the study was to compare the benefits and disadvantages of different calcimimetics in the treatment of patients with CKD with SHPT. The results showed that in achieving target PTH levels, etelcalcetide was the most effective calcimimetic, 1.78 times more effective than cinacalcet (OR 2.78; 95% CI: 1.19–6.67), and 3.93 times more effective than evocalcet (OR 4.93; 95% CI: 1.33–18.2).^140^ The above studies showed that etelcalcetide can effectively reduce PTH levels in children and adult patients of CKD with SHPT, but its application in pediatric patients is still lacking.

The recommendations in this guideline are primarily derived from direct pediatric evidence, supported by adult data. Pediatric evidence includes 1 phase III RCT demonstrating that cinacalcet significantly increases the proportion of children achieving ≥ 30% iPTH reduction compared with placebo (54.5% vs. 19%, *P* = 0.017)^136^; a prospective cohort study showing > 60% iPTH reduction from baseline^137^; and a retrospective case series in children confirming significant iPTH lowering (51% reduction).^138^ A phase I study of etelcalcetide showed acute iPTH reduction but limited pediatric data.^139^ High-quality adult evidence, including a large network meta-analysis, has established that calcimimetics effectively lower iPTH in patients of CKD with SHPT, with etelcalcetide showing superior efficacy to cinacalcet.^140^

In summary, the evidence shows that the use of cinacalcet in children with CKD can effectively correct many children with refractory SHPT without serious side effects, and can help children avoid parathyroidectomy for a considerable period.^136^, ^137^, ^138^^,^^141^, ^142^, ^143^, ^144^ Therefore, for the children over 3 years of age with CKD G4 to G5D and iPTH levels persistently higher than 300 pg/ml, cinacalcet in combination with vitamin D analogues is recommended to control iPTH within the target range in this guideline. This guideline recommends that treatment with cinacalcet should be started at a low dose (≤ 0.20 mg/kg/day), and the dose should be adjusted according to the patient’s therapeutic response. The maximum dose may be up to 2.5 mg/kg (total < 180 mg).

As children are still growing and developing, their calcium requirements are different from those of adults, and hypocalcemia should be avoided as much as possible. Serum calcium levels were monitored within 1 week of initiation of medication, weekly during the titration phase, and at least monthly during the maintenance phase. Cinacalcet should be discontinued if symptomatic hypocalcemia occurs during the treatment, including paresthesia, myalgia, cramps, convulsions, seizures, prolonged QT interval, or other serious side effects. If the level of iPTH decreases significantly or remains below the lower limit of the target range, the therapeutic drugs that reduce iPTH should be reduced or even stopped to avoid or reduce the risk of low-transport bone disease and even nondynamic bone disease. In general, there is still a lack of large-sample and high-quality RCT reports on cinacalcet and etelcalcetide in children, and more clinical trials are needed in the future.

Currently, clinical management of SHPT in combination with CKD-MBD is usually pharmacological, using osteotriol or active vitamin D analogues or calcimimetics.^145^ However, not all children’s iPTH falls to the target range with medication, and surgical treatment may be considered for children with recalcitrant SHPT who have not responded to pharmacological treatment.

A single-center retrospective study in Spain in 2018 included 13 children with chronic renal failure who underwent parathyroidectomy. The results showed that the mean PTH levels were significantly decreased at 48 hours postoperatively, whereas the mean serum calcium did not change significantly. Mean serum calcium was 9 mg/dl (IQR: 7.6–9.3 mg/dl) and mean PTH was 50 pg/ml (IQR: 28.5–108 pg/ml) in children followed up at 1 year, with a statistically significant difference in PTH decrease before and after treatment (*P* < 0.05). Two children developed bone starvation syndrome, and 1 child had a pathological relapse.^146^ A retrospective study in Argentina in 2024 included 19 children with combined refractory hyperparathyroidism on dialysis, all of whom underwent subtotal parathyroidectomy, and showed that after 1 year of surgical treatment, the children had a mean PTH level and mean ALP that were significantly lower than before, and mean Ca × P did not change significantly before and after treatment. Imaging and clinical signs of bone disease improved in all children postoperatively. Postoperatively, all patients presented with severe hungry bone syndrome and required intravenous and oral calcium and calcitriol supplementation. None of them had other postoperative complication.^147^ All the above studies have shown a significant decrease in serum PTH in children with CKD-MBD at the time of surgery, but complications such as hungry bone syndrome and hypoparathyroidism may occur after surgery.^146^, ^147^, ^148^

The evidence included in this guideline shows that surgery is effective in children with SHPT who have failed pharmacological treatment. The literature indicates that the most common complications after surgery are bone starvation syndrome, laryngeal reentrant nerve injury, and wound infection, but all these problems can be avoided by delicate intraoperative manipulation and timely management through close postoperative observation.^149^ However, there is still a lack of sufficient evidence-based medical evidence, and future clinical trials could further explore the indications for surgery in pediatric patients and the selection of optimal surgical methods for different patients.

### Conclusion and Prospect

This guideline is the first evidence-based guideline for the management of CKD-MBD in children in China, which mainly focuses on children with CKD and abnormal mineral and bone metabolism caused by various congenital or secondary kidney diseases. Based on the Grading of Recommendations, Assessment, Development, and Evaluation method, various well-known literature databases and guideline databases at home and abroad are systematically searched, and different levels of clinical evidence are included to provide diagnosis and treatment guidelines for medical professionals at all levels and types of medical institutions or units in accordance with China's national conditions. Although a large amount of clinical evidence has been extensively included in the formulation process of this guideline, and the opinions of experts in clinically related fields have also been consulted, there are still some limitations in this guideline. For example, some clinical questions have not been retrieved from high-quality literature related to children; some recommended drugs are still not available, which limits the choice of drugs for children; some recommended monitoring indicators or examinations cannot be performed in primary hospitals.

Based on the current evidence, the Guideline Development Working Group suggests the following directions for further research in the diagnosis and management of children with CKD-MBD: (ⅰ) to study the accuracy of the combined diagnosis of iPTH and BAP in the diagnosis of bone turnover in children with CKD-MBD; (ⅱ) to continue to study the sensitivity and specificity of echocardiography and lateral abdominal x-ray in the diagnosis of vascular calcification in children with CKD-MBD; (ⅲ) to continue research to explore the therapeutic effect of intensive dialysis in children with CKD-MBD complicated by hyperphosphatemia; (ⅳ) to further analyze the application effect of sevelamer in children with CKD-MBD and compare it with calcium-phosphorus binders; (ⅴ) to further study the target range of iPTH in children with CKD G4 to G5; (ⅵ) to explore the surgical indications for thyroidectomy in children and the best surgical method for different patients; and (ⅶ) management of CKD-MBD in children with kidney transplants (CKD 5T). This guideline did not address the unique challenges of CKD-MBD in children with functioning kidney transplants, including the effects of immunosuppressive medications on bone metabolism, persistence or resolution of pretransplant bone disease, and post-transplant mineral metabolism abnormalities. Given the complexity and distinct pathophysiology of CKD-MBD in the transplant setting, this topic warrants a dedicated guideline in the future.

This guideline will promote the progress of diagnosis and treatment of children with CKD-MBD and help improve the level of children's health. Towards this, we will conduct more high-level research with colleagues at home and abroad, gradually explore and verify, to build and improve the standard diagnosis and treatment system of children with CKD-MBD, and better contribute to the development of children's health.

## Appendix

### List of Members Subspecialty Group of Nephrology, Society of Pediatrics, Chinese Medical Association

Ai-Hua Zhang, Qui Li, Mo Wang, Hong Xu, Hui Wang, Chun-lin Gao, Xiao-rong Liu, Yu-bin Wu, Shu-zhen Sun, Yu-hong Tao, Xiao-shan Shao, Fang Wang, Li-Jun Zhao, Xiao-yun Jiang, Ying-jie Li, Li Yu, Zheng-kun Xia, Xi-qiang Dang, Jian-hua Mao, Jian-hua Zhou, Qian Shen, Wen-yan Huang, Xiao-wen Wang, Ying Shen, Song-ming Huang, Hui-mei Huang, and Dong-feng Zhang.

## Disclosure

All the authors declared no competing interests.

## Supplementary Material

Supplementary File (PDF) **Supplementary Material A.** Search Strategy. **Supplementary Material B.** Evidence Summary Tables. **Table S1.** Summary table of studies evaluating the ability of serum biochemical indexes to diagnose CKD-MBD in children–study characteristics. **Table S2.** Summary table of studies evaluating the ability of serum biochemical indexes to diagnose CKD-MBD in children–study population characteristics. **Table S3.** Summary table of studies evaluating the ability of serum biochemical indexes to diagnose CKD-MBD in children–results. **Table S4.** Summary table of studies evaluating the ability of serum biochemical indexes to diagnose CKD-MBD in children–quality. **Table S5.** Evidence profile of studies evaluating the ability of serum biochemical indexes to diagnose CKD-MBD in children. **Table S6.** Summary table of studies evaluating the ability of biochemical indicators to assess the nature and severity of bone abnormalities in children with CKD-MBD–study characteristics. **Table S7.** Summary table of studies evaluating the ability of biochemical indicators to assess the nature and severity of bone abnormalities in children with CKD-MBD–study population characteristics. **Table S8.** Summary table of studies evaluating the ability of biochemical indicators to assess the nature and severity of bone abnormalities in children with CKD-MBD–results. **Table S9.** Summary table of studies evaluating the ability of biochemical indicators to assess the nature and severity of bone abnormalities in children with CKD-MBD–quality. **Table S10.** Evidence profile of studies evaluating the ability of biochemical indicators to assess the nature and severity of bone abnormalities in children with CKD-MBD. **Table S11.** Summary table of studies evaluating the ability of DXA to assess the BMD and bone mass in children with CKD-MBD–study characteristics. **Table S12.** Summary table of studies evaluating the ability of DXA to assess the BMD and bone mass in children with CKD-MBD–study population characteristics. **Table S13.** Summary table of studies evaluating the ability of DXA to assess the BMD and bone mass in children with CKD-MBD–results. **Table S14.** Summary table of studies evaluating the ability of DXA to assess the BMD and bone mass in children with CKD-MBD–quality. **Table S15.** Evidence profile of studies evaluating the ability of DXA to assess the BMD and bone mass in children with CKD-MBD. **Table S16.** Summary table of studies evaluating the occasion of histological examination to assess the bone abnormalities in children with CKD-MBD–study characteristics. **Table S17.** Summary table of studies evaluating the occasion of histological examination to assess the bone abnormalities in children with CKD-MBD–study population characteristics. **Table S18.** Summary table of studies evaluating the occasion of histological examination to assess the bone abnormalities in children with CKD-MBD–results. **Table S19.** Summary table of studies evaluating the occasion of histological examination to assess the bone abnormalities in children with CKD-MBD–quality. **Table S20.** Evidence profile of studies evaluating the occasion of histological examination to assess the bone abnormalities in children with CKD-MBD. **Table S21.** Summary table of studies evaluating the ability of different imaging to detect vascular calcification in children with CKD-MBD–study characteristics. **Table S22.** Summary table of studies evaluating the ability of different imaging to detect vascular calcification in children with CKD-MBD–study population characteristics. **Table S23.** Summary table of studies evaluating the ability of different imaging to detect vascular calcification in children with CKD-MBD–results. **Table S24.** Summary table of studies evaluating the ability of different imaging to detect vascular calcification in children with CKD-MBD–quality. **Table S25.** Evidence profile of studies evaluating the ability of different imaging to detect vascular calcification in children with CKD-MBD. **Table S26.** Summary table of clinical trials examining the treatment of calcium supplementation in CKD G3a-G5D presenting with hypocalcemia–study characteristics. **Table S27.** Summary table of clinical trials examining the treatment of calcium supplementation in CKD G3a-G5D presenting with hypocalcemia–study population characteristics. **Table S28.** Summary table of clinical trials examining the treatment of calcium supplementation in CKD G3a-G5D presenting with hypocalcemia–results. **Table S29.** Summary table of clinical trials examining the treatment of calcium supplementation in CKD G3a-G5D presenting with hypocalcemia–quality. **Table S30.** Evidence profile of RCT examining the treatment of calcium supplementation in CKD G3a-G5D presenting with hypocalcemia. **Table S31.** Summary table of clinical trials examining the treatment of hyperphosphatemia with phosphate lowering drugs in CKD G3a-G5 children –study characteristics. **Table S32.** Summary table of clinical trials examining the treatment of hyperphosphatemia with phosphate lowering drugs in CKD G3a-G5 children –study population characteristics. **Table S33.** Summary table of clinical trials examining the treatment of hyperphosphatemia with phosphate lowering drugs in CKD G3a-G5 children –results. **Table S34.** Summary table of clinical trials examining the treatment of hyperphosphatemia with phosphate lowering drugs in CKD G3a-G5 children –quality. **Table S35.** Evidence profile of RCT examining the treatment of hyperphosphatemia with phosphate lowering drugs in CKD G3a-G5. **Table S36.** Summary table of clinical trials examining the dialysis regimen of hypocalcemia or hyperphosphatemia in CKD G5D–study characteristics. **Table S37.** Summary table of clinical trials examining the dialysis regimen of hypocalcemia or hyperphosphatemia in CKD G5D–study population characteristics. **Table S38.** Summary table of clinical trials examining the dialysis regimen of hypocalcemia or hyperphosphatemia in CKD G5D–results. **Table S39.** Summary table of clinical trials examining the dialysis regimen of hypocalcemia or hyperphosphatemia in CKD G5D –quality. **Table S40.** Summary table of clinical trials examining the treatment of CKD-MBD with active vitamin D in CKD G3a-G5 –study characteristics. **Table S41.** Summary table of clinical trials examining the treatment of CKD-MBD with active vitamin D in CKD G3a-G5 –study population characteristics. **Table S42.** Summary table of clinical trials examining the treatment of CKD-MBD with active vitamin D in CKD G3a-G5 –results. **Table S43.** Summary table of clinical trials examining the treatment of CKD-MBD with active vitamin D in CKD G3a-G5 –quality. **Table S44.** Evidence profile of RCT examining the treatment of CKD-MBD with active vitamin D in CKD G3a-G5. **Table S45.** Summary table of clinical trials examining the treatment of CKD-MBD with vitamin D analogues in CKD G3a-G5D – study characteristics. **Table S46.** Summary table of clinical trials examining the treatment of CKD-MBD with vitamin D analogues in CKD G3a-G5D – study population characteristics. **Table S47.** Summary table of clinical trials examining the treatment of CKD-MBD with vitamin D analogues in CKD G3a-G5D – results. **Table S48.** Summary table of clinical trials examining the treatment of CKD-MBD with vitamin D analogues in CKD G3a-G5D – quality. **Table S49.** Evidence profile of RCT examining the treatment of CKD-MBD with vitamin D analogues in CKD G3a-G5D. **Table S50.** Summary table of clinical trials examining the treatment of CKD-MBD with cinacalcet in CKD G3a-G5 –study characteristics. **Table S51.** Summary table of clinical trials examining the treatment of CKD-MBD with cinacalcet in CKD G3a-G5 – study population characteristics. **Table S52.** Summary table of clinical trials examining the treatment of CKD-MBD with cinacalcet in CKD G3a-G5 –results. **Table S53.** Summary table of clinical trials examining the treatment of CKD-MBD with cinacalcet in CKD G3a-G5 –quality. **Table S54.** Evidence profile of RCT examining the treatment of CKD-MBD with cinacalcet in CKD G3a-G5. **Supplementary Material C.** Introduction of work group membership.

## Footnotes

Supplementary file (PDF)
**Supplementary Material A.** Search Strategy. **Supplementary Material B.** Evidence Summary Tables. **Table S1.** Summary table of studies evaluating the ability of serum biochemical indexes to diagnose CKD-MBD in children–study characteristics. **Table S2.** Summary table of studies evaluating the ability of serum biochemical indexes to diagnose CKD-MBD in children–study population characteristics. **Table S3.** Summary table of studies evaluating the ability of serum biochemical indexes to diagnose CKD-MBD in children–results. **Table S4.** Summary table of studies evaluating the ability of serum biochemical indexes to diagnose CKD-MBD in children–quality. **Table S5.** Evidence profile of studies evaluating the ability of serum biochemical indexes to diagnose CKD-MBD in children. **Table S6.** Summary table of studies evaluating the ability of biochemical indicators to assess the nature and severity of bone abnormalities in children with CKD-MBD–study characteristics. **Table S7.** Summary table of studies evaluating the ability of biochemical indicators to assess the nature and severity of bone abnormalities in children with CKD-MBD–study population characteristics. **Table S8.** Summary table of studies evaluating the ability of biochemical indicators to assess the nature and severity of bone abnormalities in children with CKD-MBD–results. **Table S9.** Summary table of studies evaluating the ability of biochemical indicators to assess the nature and severity of bone abnormalities in children with CKD-MBD–quality. **Table S10.** Evidence profile of studies evaluating the ability of biochemical indicators to assess the nature and severity of bone abnormalities in children with CKD-MBD. **Table S11.** Summary table of studies evaluating the ability of DXA to assess the BMD and bone mass in children with CKD-MBD–study characteristics. **Table S12.** Summary table of studies evaluating the ability of DXA to assess the BMD and bone mass in children with CKD-MBD–study population characteristics. **Table S13.** Summary table of studies evaluating the ability of DXA to assess the BMD and bone mass in children with CKD-MBD–results. **Table S14.** Summary table of studies evaluating the ability of DXA to assess the BMD and bone mass in children with CKD-MBD–quality. **Table S15.** Evidence profile of studies evaluating the ability of DXA to assess the BMD and bone mass in children with CKD-MBD. **Table S16.** Summary table of studies evaluating the occasion of histological examination to assess the bone abnormalities in children with CKD-MBD–study characteristics. **Table S17.** Summary table of studies evaluating the occasion of histological examination to assess the bone abnormalities in children with CKD-MBD–study population characteristics. **Table S18.** Summary table of studies evaluating the occasion of histological examination to assess the bone abnormalities in children with CKD-MBD–results. **Table S19.** Summary table of studies evaluating the occasion of histological examination to assess the bone abnormalities in children with CKD-MBD–quality. **Table S20.** Evidence profile of studies evaluating the occasion of histological examination to assess the bone abnormalities in children with CKD-MBD. **Table S21.** Summary table of studies evaluating the ability of different imaging to detect vascular calcification in children with CKD-MBD–study characteristics. **Table S22.** Summary table of studies evaluating the ability of different imaging to detect vascular calcification in children with CKD-MBD–study population characteristics. **Table S23.** Summary table of studies evaluating the ability of different imaging to detect vascular calcification in children with CKD-MBD–results. **Table S24.** Summary table of studies evaluating the ability of different imaging to detect vascular calcification in children with CKD-MBD–quality. **Table S25.** Evidence profile of studies evaluating the ability of different imaging to detect vascular calcification in children with CKD-MBD. **Table S26.** Summary table of clinical trials examining the treatment of calcium supplementation in CKD G3a-G5D presenting with hypocalcemia–study characteristics. **Table S27.** Summary table of clinical trials examining the treatment of calcium supplementation in CKD G3a-G5D presenting with hypocalcemia–study population characteristics. **Table S28.** Summary table of clinical trials examining the treatment of calcium supplementation in CKD G3a-G5D presenting with hypocalcemia–results. **Table S29.** Summary table of clinical trials examining the treatment of calcium supplementation in CKD G3a-G5D presenting with hypocalcemia–quality. **Table S30.** Evidence profile of RCT examining the treatment of calcium supplementation in CKD G3a-G5D presenting with hypocalcemia. **Table S31.** Summary table of clinical trials examining the treatment of hyperphosphatemia with phosphate lowering drugs in CKD G3a-G5 children –study characteristics. **Table S32.** Summary table of clinical trials examining the treatment of hyperphosphatemia with phosphate lowering drugs in CKD G3a-G5 children –study population characteristics. **Table S33.** Summary table of clinical trials examining the treatment of hyperphosphatemia with phosphate lowering drugs in CKD G3a-G5 children –results. **Table S34.** Summary table of clinical trials examining the treatment of hyperphosphatemia with phosphate lowering drugs in CKD G3a-G5 children –quality. **Table S35.** Evidence profile of RCT examining the treatment of hyperphosphatemia with phosphate lowering drugs in CKD G3a-G5. **Table S36.** Summary table of clinical trials examining the dialysis regimen of hypocalcemia or hyperphosphatemia in CKD G5D–study characteristics. **Table S37.** Summary table of clinical trials examining the dialysis regimen of hypocalcemia or hyperphosphatemia in CKD G5D–study population characteristics. **Table S38.** Summary table of clinical trials examining the dialysis regimen of hypocalcemia or hyperphosphatemia in CKD G5D–results. **Table S39.** Summary table of clinical trials examining the dialysis regimen of hypocalcemia or hyperphosphatemia in CKD G5D –quality. **Table S40.** Summary table of clinical trials examining the treatment of CKD-MBD with active vitamin D in CKD G3a-G5 –study characteristics. **Table S41.** Summary table of clinical trials examining the treatment of CKD-MBD with active vitamin D in CKD G3a-G5 –study population characteristics. **Table S42.** Summary table of clinical trials examining the treatment of CKD-MBD with active vitamin D in CKD G3a-G5 –results. **Table S43.** Summary table of clinical trials examining the treatment of CKD-MBD with active vitamin D in CKD G3a-G5 –quality. **Table S44.** Evidence profile of RCT examining the treatment of CKD-MBD with active vitamin D in CKD G3a-G5. **Table S45.** Summary table of clinical trials examining the treatment of CKD-MBD with vitamin D analogues in CKD G3a-G5D – study characteristics. **Table S46.** Summary table of clinical trials examining the treatment of CKD-MBD with vitamin D analogues in CKD G3a-G5D – study population characteristics. **Table S47.** Summary table of clinical trials examining the treatment of CKD-MBD with vitamin D analogues in CKD G3a-G5D – results. **Table S48.** Summary table of clinical trials examining the treatment of CKD-MBD with vitamin D analogues in CKD G3a-G5D – quality. **Table S49.** Evidence profile of RCT examining the treatment of CKD-MBD with vitamin D analogues in CKD G3a-G5D. **Table S50.** Summary table of clinical trials examining the treatment of CKD-MBD with cinacalcet in CKD G3a-G5 –study characteristics. **Table S51.** Summary table of clinical trials examining the treatment of CKD-MBD with cinacalcet in CKD G3a-G5 –study population characteristics. **Table S52.** Summary table of clinical trials examining the treatment of CKD-MBD with cinacalcet in CKD G3a-G5 –results. **Table S53.** Summary table of clinical trials examining the treatment of CKD-MBD with cinacalcet in CKD G3a-G5 –quality. **Table S54.** Evidence profile of RCT examining the treatment of CKD-MBD with cinacalcet in CKD G3a-G5. **Supplementary Material C.** Introduction of work group membership.

## References

[1] Evenepoel P., Stenvinkel P., Shanahan C., Pacifici R. (2023). Inflammation and gut dysbiosis as drivers of CKD-MBD. Nat Rev Nephrol.

[2] Shi X.M., Liu B.N., Zhong X.H., Wang F., Ding J. (2019). Epidemiology of chronic kidney disease in children. Zhong Hua Er Ke Za Zhi.

[3] Goodman W.G., Goldin J., Kuizon B.D. (2000). Coronary-artery calcification in young adults with end-stage renal disease who are undergoing dialysis. N Engl J Med.

[4] Milliner D.S., Zinsmeister A.R., Lieberman E., Landing B. (1990). Soft tissue calcification in pediatric patients with end-stage renal disease. Kidney Int.

[5] Lilien M.R., Groothoff J.W. (2009). Cardiovascular disease in children with CKD or ESRD. Nat Rev Nephrol.

[6] World Health Organization (WHO) (2014). WHO handbook for guideline development, 2nd ed. https://www.who.int/publications/i/item/9789241548960.

[7] Schünemann H.J., Wiercioch W., Etxeandia I. (2014). Guidelines 2.0: Systematic development of a comprehensive checklist for a successful guideline enterprise. CMAJ.

[8] Brouwers M.C., Kho M.E., Browman G.P. (2010). AGREE II: Advancing guideline development, reporting and evaluation in health care. J Clin Epidemiol.

[9] Chen Y., Yang K., Marušic A. (2017). A reporting tool for practice guidelines in health care: the RIGHT statement. Ann Intern Med.

[10] Jacoby J., Matell M. (1971). Three-point likert scales are good enough. J Mark Res.

[11] Shea B.J., Grimshaw J.M., Wells G.A. (2007). Development of AMSTAR: a measurement tool to assess the methodological quality of systematic reviews. BMC Med Res Methodol.

[12] Higgins J.P., Altman D.G., Gøtzsche P.C. (2011). The Cochrane Collaboration’s tool for assessing risk of bias in randomised trials. BMJ.

[13] Wells G.A., Shea B., O’Connell D. The Newcastle-Ottawa Scale (NOS) for assessing the quality of nonrandomised studies in meta-analyses. The Ottawa Hospital Research Institute. https://www.ohri.ca/programs/clinical_epidemiology/oxford.asp.

[14] Bylund S., Kobyletzki L.B., Svalstedt M., Svensson Å. (2020). Prevalence and incidence of atopic dermatitis: a systematic review. Acta Derm Venereol.

[15] Jaeschke R., Guyatt G.H., Dellinger P. (2008). Use of GRADE grid to reach decisions on clinical practice guidelines when consensus is elusive. BMJ.

[16] Expert Group of Evidence-Based Guidelines for the Diagnosis and Treatment of Mineral and Bone Abnormalities in Children With Chronic Kidney Disease in China (2021). Protocol of evidence-based guidelines for the diagnosis and treatment of mineral and bone abnormalities in Chinese children with chronic kidney disease. Chin J Evid Based Pediatr.

[17] Kidney Disease: Improving Global Outcomes (KDIGO) CKD-MBD Update Work Group (2017). KDIGO 2017 clinical practice guideline update for the diagnosis, evaluation, prevention, and treatment of chronic kidney disease-mineral and bone disorder (CKD-MBD). Kidney Int Suppl (2011).

[18] Drüeke T.B., Massy Z.A. (2016). Changing bone patterns with progression of chronic kidney disease. Kidney Int.

[19] Lerch C., Shroff R., Wan M. (2018). Effects of nutritional vitamin D supplementation on markers of bone and mineral metabolism in children with chronic kidney disease. Nephrol Dial Transplant.

[20] Doyon A., Fischer D.C., Bayazit A.K. (2015). Markers of bone metabolism are affected by renal function and growth hormone therapy in children with chronic kidney disease. PLoS One.

[21] Klaus G., Watson A., Edefonti A. (2006). Prevention and treatment of renal osteodystrophy in children on chronic renal failure: European guidelines. Pediatr Nephrol.

[22] Bakkaloglu S.A., Bacchetta J., Lalayiannis A.D. (2021). Bone evaluation in paediatric chronic kidney disease: clinical practice points from the European Society for Paediatric Nephrology CKD-MBD and Dialysis working groups and CKD-MBD working group of the ERA-EDTA. Nephrol Dial Transplant.

[23] Kaspar C.D., Bholah R., Bunchman T.E. (2016). A review of pediatric chronic kidney disease. Blood Purif.

[24] Drube J., Wan M., Bonthuis M. (2019). Clinical practice recommendations for growth hormone treatment in children with chronic kidney disease. Nat Rev Nephrol.

[25] Group K.W. (2009). KDOQI Clinical Practice Guideline for Nutrition in Children with CKD: 2008 update. Executive summary. Am J Kidney Dis.

[26] Sprague S.M., Bellorin-Font E., Jorgetti V. (2016). Diagnostic accuracy of bone turnover markers and bone histology in patients with CKD treated by dialysis. Am J Kidney Dis.

[27] Liangos O., Kirchhoff S., Buchholz J., Ketteler M. (2018). Bone biopsy results in chronic kidney disease: a single-center experience. Kidney Blood Press Res.

[28] Barreto F.C., Barreto D.V., Moysés R.M. (2008). K/DOQI-recommended intact PTH levels do not prevent low-turnover bone disease in hemodialysis patients. Kidney Int.

[29] Salam S., Gallagher O., Gossiel F., Paggiosi M., Khwaja A., Eastell R. (2018). Diagnostic accuracy of biomarkers and imaging for bone turnover in renal osteodystrophy. J Am Soc Nephrol.

[30] Shroff R., Lalayiannis A.D., Fewtrell M. (2022). Naturally occurring stable calcium isotope ratios are a novel biomarker of bone calcium balance in chronic kidney disease. Kidney Int.

[31] Denburg M.R., Kumar J., Jemielita T. (2016). Fracture burden and risk factors in childhood CKD: results from the CKiD cohort study. J Am Soc Nephrol.

[32] Groothoff J.W., Offringa M., Van Eck-Smit B.L. (2003). Severe bone disease and low bone mineral density after juvenile renal failure. Kidney Int.

[33] Lalayiannis A.D., Crabtree N.J., Fewtrell M. (2020). Assessing bone mineralisation in children with chronic kidney disease: what clinical and research tools are available?. Pediatr Nephrol.

[34] Lalayiannis A.D., Crabtree N.J., Ferro C.J. (2021). Routine serum biomarkers, but not dual-energy x-ray absorptiometry, correlate with cortical bone mineral density in children and young adults with chronic kidney disease. Nephrol Dial Transplant.

[35] Bakr A.M. (2004). Bone mineral density and bone turnover markers in children with chronic renal failure. Pediatr Nephrol.

[36] Salem N., Bakr A., Eid R. (2023). Trabecular bone score in assessing bone mineralization status in children with end- stage renal disease: a promising tool. Eur J Pediatr.

[37] Bianchi M.L., Leonard M.B., Bechtold S. (2014). Bone health in children and adolescents with chronic diseases that may affect the skeleton: the 2013 ISCD Pediatric Official Positions. J Clin Densitom.

[38] Fewtrell M.S., British Paediatric & Adolescent Bone Group (2003). Bone densitometry in children assessed by dual x ray absorptiometry: uses and pitfalls. Arch Dis Child.

[39] Griffin L.M., Kalkwarf H.J., Zemel B.S. (2012). Assessment of dual-energy x-ray absorptiometry measures of bone health in pediatric chronic kidney disease. Pediatr Nephrol.

[40] Iimori S., Mori Y., Akita W. (2012). Diagnostic usefulness of bone mineral density and biochemical markers of bone turnover in predicting fracture in CKD stage 5D patients--a single-center cohort study. Nephrol Dial Transplant.

[41] Naylor K.L., Garg A.X., Zou G. (2015). Comparison of fracture risk prediction among individuals with reduced and normal kidney function. Clin J Am Soc Nephrol.

[42] Yenchek R.H., Ix J.H., Shlipak M.G. (2012). Bone mineral density and fracture risk in older individuals with CKD. Clin J Am Soc Nephrol.

[43] Moe S., Drüeke T., Cunningham J. (2006). Definition, evaluation, and classification of renal osteodystrophy: a position statement from Kidney Disease: improving Global Outcomes (KDIGO). Kidney Int.

[44] Salusky I.B., Coburn J.W., Brill J. (1988). Bone disease in pediatric patients undergoing dialysis with CAPD or CCPD. Kidney Int.

[45] Mathias R., Salusky I., Harman W. (1993). Renal bone disease in pediatric and young adult patients on hemodialysis in a Children’s Hospital. J Am Soc Nephrol.

[46] Salusky I.B., Ramirez J.A., Oppenheim W., Gales B., Segre G.V., Goodman W.G. (1994). Biochemical markers of renal osteodystrophy in pediatric patients undergoing CAPD/CCPD. Kidney Int.

[47] Yalçinkaya F., Ince E., Tümer N., Ensari A., Ozkaya N. (2000). Spectrum of renal osteodystrophy in children on continuous ambulatory peritoneal dialysis. Pediatr Int.

[48] Ziólkowska H., Pańiczyk-Tomaszewska M., Debiński A., Polowiec Z., Sawicki A., Sieniawska M. (2000). Bone biopsy results and serum bone turnover parameters in uremic children. Acta Paediatr.

[49] Waller S., Shroff R., Freemont A.J., Rees L. (2008). Bone histomorphometry in children prior to commencing renal replacement therapy. Pediatr Nephrol.

[50] Bakkaloglu S.A., Wesseling-Perry K., Pereira R.C. (2010). Value of the new bone classification system in pediatric renal osteodystrophy. Clin J Am Soc Nephrol.

[51] Pereira R.C., Bischoff D.S., Yamaguchi D., Salusky I.B., Wesseling-Perry K. (2016). Micro-CT in the assessment of pediatric renal osteodystrophy by bone histomorphometry. Clin J Am Soc Nephrol.

[52] Carvalho C.G., Pereira R.C., Gales B., Salusky I.B., Wesseling-Perry K. (2015). Cortical and trabecular bone in pediatric end-stage kidney disease. Pediatr Nephrol.

[53] Soeiro E.M.D., Castro L., Menezes R. (2020). Association of parathormone and alkaline phosphatase with bone turnover and mineralization in children with CKD on dialysis: effect of age, gender, and race. Pediatr Nephrol.

[54] Wesseling-Perry K., Pereira R.C., Tseng C.H. (2012). Early skeletal and biochemical alterations in pediatric chronic kidney disease. Clin J Am Soc Nephrol.

[55] Bover J., Ureña P., Brandenburg V. (2014). Adynamic bone disease: from bone to vessels in chronic kidney disease. Semin Nephrol.

[56] Ferreira A., Frazão J.M., Monier-Faugere M.C. (2008). Effects of sevelamer hydrochloride and calcium carbonate on renal osteodystrophy in hemodialysis patients. J Am Soc Nephrol.

[57] Ketteler M., Block G.A., Evenepoel P. (2018). Diagnosis, evaluation, prevention, and treatment of chronic kidney disease-mineral and bone disorder: synopsis of the kidney disease: improving global outcomes 2017 clinical practice guideline update. Ann Intern Med.

[58] Srivaths P., Krishnamurthy R., Brunner L. (2014). Cardiac calcifications are more prevalent in children receiving hemodialysis than peritoneal dialysis. Clin Nephrol.

[59] Cozzolino M., Galassi A., Biondi M.L. (2006). Serum fetuin-A levels link inflammation and cardiovascular calcification in hemodialysis patients. Am J Nephrol.

[60] Toussaint N.D., Pedagogos E., Lau K.K. (2011). Lateral lumbar X-ray assessment of abdominal aortic calcification in Australian haemodialysis patients. Nephrology (Carlton).

[61] Yao Z., Wang C., Zhang Q., Ma S., Gui B., Duan C. (2017). Prevalence of abdominal artery calcification in dialysis patients with end-stage renal disease: a systematic review and meta-analysis. Int Urol Nephrol.

[62] Voelkl J., Cejka D., Alesutan I. (2019). An overview of the mechanisms in vascular calcification during chronic kidney disease. Curr Opin Nephrol Hypertens.

[63] Disthabanchong S., Srisuwarn P. (2019). Mechanisms of vascular calcification in kidney disease. Adv Chronic Kidney Dis.

[64] Lalayiannis A.D., Ferro C.J., Wheeler D.C. (2021). The burden of subclinical cardiovascular disease in children and young adults with chronic kidney disease and on dialysis. Clin Kidney J.

[65] Lalayiannis A.D., Crabtree N.J., Ferro C.J. (2022). Bone mineral density and vascular calcification in children and young adults with CKD 4 to 5 or on dialysis. Kidney Int Rep.

[66] Liu Z.H., Yu X.Q., Yang J.W. (2018). Prevalence and risk factors for vascular calcification in Chinese patients receiving dialysis: baseline results from a prospective cohort study. Curr Med Res Opin.

[67] Bacchetta J., Harambat J., Cochat P., Salusky I.B., Wesseling-Perry K. (2012). The consequences of chronic kidney disease on bone metabolism and growth in children. Nephrol Dial Transplant.

[68] Borzych D., Rees L., Ha I.S. (2010). The bone and mineral disorder of children undergoing chronic peritoneal dialysis. Kidney Int.

[69] Melo V.B., Silva D.B.D., Soeiro M.D. (2024). Growth in children with chronic kidney disease and associated risk factors for short stature. J Bras Nefrol.

[70] Wesseling-Perry K. (2015). Defective skeletal mineralization in pediatric CKD. Curr Osteoporos Rep.

[71] Young E.W., Albert J.M., Satayathum S. (2005). Predictors and consequences of altered mineral metabolism: the Dialysis Outcomes and Practice Patterns Study. Kidney Int.

[72] El-Gamasy M.A., El-Shehaby W.A., Mabrouk M.M. (2019). Early predictors of cardiac dysfunction in Egyptian children with chronic kidney disease. Ann Pediatr Cardiol.

[73] Shroff R.C., Donald A.E., Hiorns M.P. (2007). Mineral metabolism and vascular damage in children on dialysis. J Am Soc Nephrol.

[74] Fukagawa M., Yokoyama K., Koiwa F. (2013). Clinical practice guideline for the management of chronic kidney disease-mineral and bone disorder. Ther Apher Dial.

[75] Shkembi B., Huppertz T. (2021). Calcium absorption from Food Products: food matrix effects. Nutrients.

[76] Miller D.D. (1989). Calcium in the diet: food sources, recommended intakes, and nutritional bioavailability. Adv Food Nutr Res.

[77] Buzinaro E.F., Almeida R.N., Mazeto G.M. (2006). Biodisponibilidade do cálcio dietético Bioavailability of dietary calcium. Arq Bras Endocrinol Metabol.

[78] St-Jules D.E., Jagannathan R., Gutekunst L., Kalantar-Zadeh K., Sevick M.A. (2017). Examining the proportion of dietary phosphorus from plants, animals, and food additives excreted in urine. J Ren Nutr.

[79] Cunningham J., Locatelli F., Rodriguez M. (2011). Secondary hyperparathyroidism: pathogenesis, disease progression, and therapeutic options. Clin J Am Soc Nephrol.

[80] Hothi D.K., Harvey E., Piva E., Keating L., Secker D., Geary D.F. (2006). Calcium and phosphate balance in adolescents on home nocturnal haemodialysis. Pediatr Nephrol (Berlin, Germany).

[81] Rastogi A., Bhatt N., Rossetti S., Beto J. (2021). Management of hyperphosphatemia in end-stage renal disease: A new paradigm. J Ren Nutr.

[82] McAlister L., Pugh P., Greenbaum L. (2020). The dietary management of calcium and phosphate in children with CKD stages 2–5 and on dialysis-clinical practice recommendation from the Pediatric Renal Nutrition Taskforce. Pediatr Nephrol.

[83] Gulati S., Sharma R.K., Gulati K., Singh U., Srivastava A. (2005). Longitudinal follow-up of bone mineral density in children with nephrotic syndrome and the role of calcium and vitamin D supplements. Nephrol Dial Transplant.

[84] Qunibi W., Winkelmayer W.C., Solomon R. (2011). A randomized, double-blind, placebo-controlled trial of calcium acetate on serum phosphorus concentrations in patients with advanced non-dialysis-dependent chronic kidney disease. BMC Nephrol.

[85] Phelps K.R., Stern M., Slingerland A., Heravi M., Strogatz D.S., Haqqie S.S. (2002). Metabolic and skeletal effects of low and high doses of calcium acetate in patients with preterminal chronic renal failure. Am J Nephrol.

[86] Cozzolino M., Bernard L., Csomor P.A. (2021). Active vitamin D increases the risk of hypercalcaemia in non-dialysis chronic kidney disease patients with secondary hyperparathyroidism: a systematic review and meta-analysis. Clin Kidney J.

[87] Navaneethan S.D., Palmer S.C., Vecchio M., Craig J.C., Elder G.J., Strippoli G.F. (2011). Phosphate binders for preventing and treating bone disease in chronic kidney disease patients. Cochrane Database Syst Rev.

[88] Toida T., Fukudome K., Fujimoto S. (2012). Effect of lanthanum carbonate vs. calcium carbonate on serum calcium in hemodialysis patients: a crossover study. Clin Nephrol.

[89] Sprague S.M., Abboud H., Qiu P., Dauphin M., Zhang P., Finn W. (2009). Lanthanum carbonate reduces phosphorus burden in patients with CKD stages 3 and 4: a randomized trial. Clin J Am Soc Nephrol.

[90] Zhai C.J., Yu X.S., Yang X.W., Sun J., Wang R. (2015). Effects and safety of iron-based phosphate binders in dialysis patients: a systematic review and meta-analysis. Ren Fail.

[91] Hahn D., Hodson E.M., Craig J.C. (2015). Interventions for metabolic bone disease in children with chronic kidney disease. Cochrane Database Syst Rev.

[92] Caglar K., Yilmaz M.I., Saglam M. (2008). Short-term treatment with sevelamer increases serum fetuin-a concentration and improves endothelial dysfunction in chronic kidney disease stage 4 patients. Clin J Am Soc Nephrol.

[93] Locatelli F., Dimkovic N., Spasovski G. (2013). Evaluation of colestilan in chronic kidney disease dialysis patients with hyperphosphataemia and dyslipidaemia: a randomized, placebo-controlled, multiple fixed-dose trial. Nephrol Dial Transplant.

[94] Block G.A., Rosenbaum D.P., Yan A., Chertow G.M. (2019). Efficacy and safety of tenapanor in patients with hyperphosphatemia receiving maintenance hemodialysis: a randomized Phase 3 trial. J Am Soc Nephrol.

[95] Silva A.L., Chertow G.M., Hernandez G.T. (2023). Tenapanor improves long-term control of hyperphosphatemia in patients receiving maintenance dialysis: the NORMALIZE study. Kidney360.

[96] Sprague S.M., Weiner D.E., Tietjen D.P. (2024). Tenapanor as therapy for hyperphosphatemia in maintenance dialysis patients: results from the OPTIMIZE study. Kidney360.

[97] Liu X.Y., Yao J.R., Xu R. (2020). Investigation of nicotinamide as more than an anti-phosphorus drug in chronic hemodialysis patients: a single-center, double-blind, randomized, placebo-controlled trial. Ann Transl Med.

[98] Lesley R., Rukshana S. (2015). The demise of calcium-based phosphate binders-is this appropriate for children?. Pediatr Nephrol.

[99] Ercan O., Gulay A., Selen B. (2016). Reduction of dialysate calcium level reduces progression of coronary artery calcification and improves low bone turnover in patients on hemodialysis. J Am Soc Nephrol.

[100] Emilio G.P., Luisa G.C.M., Dolores A.M. (2014). Individualization of dialysate calcium concentration according to baseline pre-dialysis serum calcium. Blood Purif.

[101] Hui K.S., Hyang C.K., Won P.J., Yoon K.W., Do J.Y. (2012). Low-calcium dialysate as a risk factor for decline in bone mineral density in peritoneal dialysis patients. Scand J Urol Nephrol.

[102] National Kidney Foundation (2003). K/DOQI clinical practice guidelines for bone metabolism and disease in chronic kidney disease. Am J Kidney Dis.

[103] Peter S.C., Bakkaloglu Sevcan A., Günter K., Schröder C., Fischbach M., European Pediatric Dialysis Working Group (2011). Solutions for peritoneal dialysis in children: recommendations by the European Pediatric Dialysis Working Group. Pediatr Nephrol.

[104] Damien A., Natalie B., James B. (2019). Renal Association clinical practice guideline on haemodialysis. BMC Nephrol.

[105] Chertow Glenn M., Levin Nathan W., FHN Trial Group (2010). In-center hemodialysis six times per week versus three times per week. N Engl J Med.

[106] Zimmerman Deborah L., Marcel R., Paul H., Fergusson D., Touyz R.M., Burns K.D. (2014). Short daily versus conventional hemodialysis for hypertensive patients: a randomized cross-over study. PLoS One.

[107] Ercan O., Soner D., Gulay A. (2011). Comparison of 4- and 8-h dialysis sessions in thrice-weekly in-centre haemodialysis: a prospective, case-controlled study. Nephrol Dial Transplant.

[108] Eduardo L., Jianglin X., Suri Rita S. (2012). Survival with three-times weekly in-center nocturnal versus conventional hemodialysis. J Am Soc Nephrol.

[109] Rocco Michael V., Lockridge Robert S., Beck Gerald J. (2011). The effects of frequent nocturnal home hemodialysis: the Frequent Hemodialysis Network Nocturnal Trial. Kidney Int.

[110] Unruh Mark L., Brett L., Eggers Paul W. (2016). The effect of frequent hemodialysis on self-reported sleep quality: Frequent Hemodialysis Network Trials. Nephrol Dial Transplant.

[111] Anne H., von Puttkamer C., Ursula L. (2011). A hospital-based intermittent nocturnal hemodialysis program for children and adolescents. J Pediatr.

[112] Fischbach M., Joëlle T., Vincent L. (2004). Daily on-line haemodiafiltration: a pilot trial in children. Nephrol Dial Transplant.

[113] Shroff R., Wan M., Nagler E.V. (2017). Clinical practice recommendations for native vitamin D therapy in children with chronic kidney disease Stages 2–5 and on dialysis. Nephrol Dial Transplant.

[114] Holick M.F., Binkley N.C., Bischoff-Ferrari H.A. (2011). Evaluation, treatment, and prevention of vitamin D deficiency: an Endocrine Society clinical practice guideline. J Clin Endocrinol Metab.

[115] Zamoner S.M.S., Takase H.M., Riyuzo M.C., Caramori J.C.T., de Andrade L.G.M. (2024). Safety of Cinacalcet in children and adolescents with chronic kidney disease-mineral bone disorder: systematic review and proportional meta-analysis of case series. Int Urol Nephrol.

[116] Tripkovic L., Lambert H., Hart K. (2012). Comparison of vitamin D2 and vitamin D3 supplementation in raising serum 25-hydroxyvitamin D status: a systematic review and meta-analysis. Am J Clin Nutr.

[117] Graeff-Armas L.A., Kaufmann M., Lyden E., Jones G. (2018). Serum 24,25-dihydroxyvitamin D3 response to native vitamin D2 and D3 Supplementation in patients with chronic kidney disease on hemodialysis. Clin Nutr.

[118] Portale A.A., Wolf M., Jüppner H. (2014). Disordered FGF23 and mineral metabolism in children with CKD. Clin J Am Soc Nephrol.

[119] Soliman M., Hassan W., Yaseen M., Rao M., Sawaya B.P., El-Husseini A. (2019). PTH assays in dialysis patients: practical considerations. Semin Dial.

[120] Greenbaum L.A., Grenda R., Qiu P. (2005). Intravenous calcitriol for treatment of hyperparathyroidism in children on hemodialysis. Pediatr Nephrol.

[121] Schmitt C.P., Ardissino G., Testa S., Claris-Appiani A., Mehls O. (2003). Growth in children with chronic renal failure on intermittent versus daily calcitriol. Pediatr Nephrol.

[122] Ardissino G., Schmitt C.P., Testa S., Claris-Appiani A., Mehls O. (2000). Calcitriol pulse therapy is not more effective than daily calcitriol therapy in controlling secondary hyperparathyroidism in children with chronic renal failure. European Study Group on Vitamin D in children with renal failure. Pediatr Nephrol.

[123] Freundlich M., Abitbol C.L. (2017). Oral paricalcitol: expanding therapeutic options for pediatric chronic kidney disease patients. Pediatr Nephrol.

[124] Teng M., Wolf M., Lowrie E., Ofsthun N., Lazarus J.M., Thadhani R. (2003). Survival of patients undergoing hemodialysis with paricalcitol or calcitriol therapy. N Engl J Med.

[125] Tonbul H.Z., Solak Y., Atalay H., Turkmen K., Altintepe L. (2012). Efficacy and tolerability of intravenous paricalcitol in calcitriol-resistant hemodialysis patients with secondary hyperparathyroidism: 12-month prospective study. Ren Fail.

[126] Culebras C., Bosch E., Baamonde E. (2011). Paricalcitol therapy improves ventricular hypertrophy in hemodialysis patients not treated with RAS inhibitors. Eur Heart J.

[127] Sarić M., Milinković N., Pejanović S., Stošović M., Naumović R., Ignjatović S. (2017). The effectiveness of vitamin D therapy in maintenance hemodialysis patients: its impact on bone health. Clin Chem Lab Med.

[128] Ala-Houhala M., Holmberg C., Rönnholm K., Paganus A., Laine J., Koskimies O. (1995). Alphacalcidol oral pulses normalize uremic hyperparathyroidism prior to dialysis. Pediatr Nephrol.

[129] Hisano S., Yamane I., Ueda K., Kawagoe M. (1990). Renal osteodystrophy in patients on continuous ambulatory peritoneal dialysis. Acta Paediatr Jpn.

[130] Fazel M., Khari S., Gubari M.I.M., Ataei N., Yousefifard M., Hosseini M. (2020). The efficacy of paricalcitol administration for management of pediatric chronic kidney disease: a systematic review and meta-analysis. Int J Pediatr-Mashhad.

[131] Ardissino G., Schmitt C.P., Bianchi M.L., Daccò V., Claris-Appiani A., Mehls O. (2000). No difference in intestinal strontium absorption after oral or IV calcitriol in children with secondary hyperparathyroidism. The European Study Group on Vitamin D in children with renal failure. Kidney Int.

[132] Jones C.L., Vieth R., Spino M. (1994). Comparisons between oral and intraperitoneal 1,25-dihydroxyvitamin D3 therapy in children treated with peritoneal dialysis. Clin Nephrol.

[133] Wesseling-Perry K., Pereira R.C., Sahney S. (2011). Calcitriol and doxercalciferol are equivalent in controlling bone turnover, suppressing parathyroid hormone, and increasing fibroblast growth factor-23 in secondary hyperparathyroidism. Kidney Int.

[134] Haffner D., Fischer D.C. (2010). Bone cell biology and pediatric renal osteodystrophy. Minerva Pediatr.

[135] Cannata-Andía J.B., Rodriguez-García M., Román-García P., Tuñón-le Poultel D., López-Hernández F., Rodríguez-Puyol D. (2010). New therapies: calcimimetics, phosphate binders and vitamin D receptor activators. Pediatr Nephrol.

[136] Warady B.A., Iles J.N., Ariceta G. (2019). A randomized, double-blind, placebo-controlled study to assess the efficacy and safety of Cinacalcet in pediatric patients with chronic kidney disease and secondary hyperparathyroidism receiving dialysis. Pediatr Nephrol.

[137] Alharthi A.A., Kamal N.M., Abukhatwah M.W., Sherief L.M. (2015). Cinacalcet in pediatric and adolescent chronic kidney disease: a single-center experience. Medicine (Baltimore).

[138] Joseph C., Shah S., Geer J., Juarez-Calderon M., Srivaths P.R., Swartz S.J. (2019). Cinacalcet for secondary hyperparathyroidism in end-stage renal disease patients below age 5 years. Clin Nephrol.

[139] Sohn W., Salusky I.B., Schmitt C.P. (2021). Phase 1, single-dose study to assess the safety, tolerability, pharmacokinetics, and pharmacodynamics of etelcalcetide in pediatric patients with secondary hyperparathyroidism receiving hemodialysis. Pediatr Nephrol.

[140] Palmer S.C., Mavridis D., Johnson D.W., Tonelli M., Ruospo M., Strippoli G.F.M. (2020). Comparative effectiveness of calcimimetic agents for secondary hyperparathyroidism in adults: a systematic review and network meta-analysis. Am J Kidney Dis.

[141] Arenas Morales A.J., DeFreitas M.J., Katsoufis C.P. (2019). Cinacalcet as rescue therapy for refractory hyperparathyroidism in young children with advanced chronic kidney disease. Pediatr Nephrol.

[142] Platt C., Inward C., McGraw M. (2010). Middle-term use of Cinacalcet in paediatric dialysis patients. Pediatr Nephrol.

[143] Muscheites J., Wigger M., Drueckler E., Fischer D.C., Kundt G., Haffner D. (2008). Cinacalcet for secondary hyperparathyroidism in children with end-stage renal disease. Pediatr Nephrol.

[144] Warady B.A., Ng E., Bloss L., Mo M., Schaefer F., Bacchetta J. (2020). Cinacalcet studies in pediatric subjects with secondary hyperparathyroidism receiving dialysis. Pediatr Nephrol.

[145] Sanchez C.P. (2003). Secondary hyperparathyroidism in children with chronic renal failure: pathogenesis and treatment. Paediatr Drugs.

[146] Ferraris T., Toselli L., Udaquiola J. (2018). Total parathyroidectomy, autoimplant and cryopreservation for the treatment of hyperparathyroidism of renal origin in children and young adults. Cir Pediatr.

[147] Gil S.M., Aziz M., De Dona V. (2024). Surgical treatment of secondary hyperparathyroidism in children with chronic kidney disease. Experience in 19 patients. J Pediatr Endocrinol Metab.

[148] Adragna M. (2016). Subtotal parathyroidectomy: last recourse for treatment of secondary hyperparathyroidism in children with chronic renal disease. Pediatr Nephrol.

[149] Apetrii M., Goldsmith D., Nistor I. (2017). Impact of surgical parathyroidectomy on chronic kidney disease-mineral and bone disorder (CKD-MBD) - A systematic review and meta-analysis. PLoS One.

