# Supplementary material for: Evidence-Based Guidelines for the Diagnosis and Treatment of Pediatric CKD-Mineral and Bone Disorder (Version 2024)
Source: Kidney Int Rep. 2026 Jun 3;11(6 Suppl):106492. doi: 10.1016/j.ekir.2026.106492 (PMC13251216; doi:10.1016/j.ekir.2026.106492)
Supplement: Supplementary File (PDF) — Supplementary Material A. Search Strategy. Supplementary Material B. Evidence Summary Tables. Table S1. Summary table of studies evaluating the ability of serum biochemical indexes to diagnose CKD-MBD in children–study characteristics. Table S2. Summary table of studies evaluating the ability of serum biochemical indexes to diagnose CKD-MBD in children–study population characteristics. Table S3. Summary table of studies evaluating the ability of serum biochemical indexes to diagnose CKD-MBD in children–results. Table S4. Summary table of studies evaluating the ability of serum biochemical indexes to diagnose CKD-MBD in children–quality. Table S5. Evidence profile of studies evaluating the ability of serum biochemical indexes to diagnose CKD-MBD in children. Table S6. Summary table of studies evaluating the ability of biochemical indicators to assess the nature and severity of bone abnormalities in children with CKD-MBD–study characteristics. Table S7. Summary table of studies evaluating the ability of biochemical indicators to assess the nature and severity of bone abnormalities in children with CKD-MBD–study population characteristics. Table S8. Summary table of studies evaluating the ability of biochemical indicators to assess the nature and severity of bone abnormalities in children with CKD-MBD–results. Table S9. Summary table of studies evaluating the ability of biochemical indicators to assess the nature and severity of bone abnormalities in children with CKD-MBD–quality. Table S10. Evidence profile of studies evaluating the ability of biochemical indicators to assess the nature and severity of bone abnormalities in children with CKD-MBD. Table S11. Summary table of studies evaluating the ability of DXA to assess the BMD and bone mass in children with CKD-MBD–study characteristics. Table S12. Summary table of studies evaluating the ability of DXA to assess the BMD and bone mass in children with CKD-MBD–study population characteristics. Table S13. Summary table [file mmc1.pdf]

Evidence-based Guidelines for the Diagnosis and Treatment of Pediatric Chronic Kidney Disease-Mineral and Bone Disorder (Version 2024) - Supplementary Material

The supplementary material includes search strategy, 54 evidence summary tables, and the introduction of work group membership.

2 Appendix A. Search Strategy

5 Appendix B. Evidence Summary Tables

94 Appendix C. Introduction of work group membership

Appendix A. Search Strategy

Search #	String
1	((((((((((KIDNEY DISEASES[MeSH Terms]) OR (KIDNEY FAILURE CHRONIC[Title/Abstract])) OR (KIDNEY FAILURE[Title/Abstract])) OR (RENAL REPLACEMENT THERAPY[Title/Abstract])) OR (RENAL DIALYSIS[Title/Abstract])) OR (HEMOFILTRATION[Title/Abstract])) OR (chronic next kidney[Title/Abstract])) OR (chronic next renal[Title/Abstract])) OR (ckd[Title/Abstract] OR ckf[Title/Abstract] OR crd[Title/Abstract] OR crf[Title/Abstract] OR eskd[Title/Abstract] OR esrd[Title/Abstract] OR eskf[Title/Abstract] OR esrf[Title/Abstract])) OR (predialysis[Title/Abstract] OR dialysis[Title/Abstract])) OR (haemodialysis[Title/Abstract] OR haemodialysis[Title/Abstract])) OR (capd[Title/Abstract] OR ccpd[Title/Abstract] OR apd[Title/Abstract])
2	(((((BONE DISEASES[MeSH Terms]) OR (RENAL OSTEODYSTROPHY[Title/Abstract])) OR (bone next disease*[Title/Abstract])) OR bone*[Title/Abstract] AND (atroph*[Title/Abstract] OR formation[Title/Abstract] OR deform*[Title/Abstract] OR destruct*[Title/Abstract] OR necrosis[Title/Abstract] OR resorption[Title/Abstract] OR metabol*[Title/Abstract] OR turnover[Title/Abstract] OR demineral*[Title/Abstract] OR decalcif*[Title/Abstract] OR density[Title/Abstract])) OR (renal osteopathy[Title/Abstract])) OR (renal rickets[Title/Abstract])
3	((((((((((((((((((((((((((((Adolescent[MeSH Terms]) OR (Adolescen*[Title/Abstract])) OR (Teen*[Title/Abstract])) OR (Youth*[Title/Abstract])) OR (juvenile*[Title/Abstract])) OR (puberty[Title/Abstract])) OR (young*[Title/Abstract])) OR (Child[Title/Abstract])) OR (Child[MeSH Terms])) OR (Pediatrics[MeSH Terms])) OR (pediatric*[Title/Abstract])) OR (paediatric*[Title/Abstract])) OR (infant*[Title/Abstract])) OR (infant[MeSH Terms])) OR (neonat*[Title/Abstract])) OR (newborn*[Title/Abstract])) OR (Baby[Title/Abstract])) OR (Babies[Title/Abstract])) OR (trottie*[Title/Abstract])) OR (Kids[Title/Abstract])) OR (toddler*[Title/Abstract])) OR (pre-school*[Title/Abstract])) OR (preschool*[Title/Abstract])) OR (kindergarten*[Title/Abstract])) OR (kinder-garten*[Title/Abstract])) OR (girl*[Title/Abstract])) OR (boy*[Title/Abstract])) OR (student*[Title/Abstract])) OR (junior*[Title/Abstract])) OR (Pubescent[Title/Abstract])
4	((((((((((((((((((((((((((biochemical indicators[Title/Abstract]) OR (Biochemical indexes[Title/Abstract])) OR (biochemical indices[Title/Abstract])) OR (Ca[Title/Abstract])) OR (calcium[Title/Abstract])) OR (P[Title/Abstract])) OR (phosphorus[Title/Abstract])) OR (Alkaline Phosphatase[Title/Abstract])) OR (ALP[Title/Abstract])) OR (AKP[Title/Abstract])) OR (ALK-P[Title/Abstract])) OR (Parathyroid Hormone[Title/Abstract])) OR (PTH[Title/Abstract])) OR (iPTH[Title/Abstract])) OR (Vitamin D[Title/Abstract])) OR (vitD[Title/Abstract])) OR (VD[Title/Abstract]) OR (1-25(OH)2 vitamin D[Title/Abstract])) OR (1-25(OH)3 vitamin D[Title/Abstract])) OR (fibroblast growth factor 23[Title/Abstract])) OR

	(FGF23[Title/Abstract])) OR (klotho[Title/Abstract])) OR (activin A[Title/Abstract])) OR (PICP[Title/Abstract])) OR (PINP[Title/Abstract])) OR (fibroblast growth factor 21[Title/Abstract])) OR (FGF21[Title/Abstract])) OR (tumer necrosis factor-alpha[Title/Abstract])) OR (TNF-α[Title/Abstract]))
5	((((((((((((examination*[Title/Abstract]) OR (assistant examination*[Title/Abstract])) OR (auxiliary examination*[Title/Abstract])) OR (accessory examination*[Title/Abstract])) OR (imaging test*[Title/Abstract])) OR (Bone biopsy[Title/Abstract])) OR (Tomography Scanners, X-Ray Computed[Title/Abstract])) OR (DXA[Title/Abstract])) OR (Dual-energy X-ray[Title/Abstract])) OR (CT[Title/Abstract])) OR (qCT[Title/Abstract])) OR (MRI[Title/Abstract]))) OR (ultrasound[Title/Abstract])
6	((((Calcinosis[MeSH Terms]) OR (Calcinoses[Title/Abstract])) OR (Pathologic Calcification[Title/Abstract])) OR (Microcalcification*[Title/Abstract])) OR (Microcalcinos*[Title/Abstract])
7	((((((((((((Tomography, X-Ray Computed[MeSH Terms]) OR (X-Ray Computer Assisted Tomography[Title/Abstract])) OR (Computed X Ray Tomography[Title/Abstract])) OR (X-Ray Computerized Tomography[Title/Abstract])) OR (CT X Ray*[Title/Abstract])) OR (CAT Scan, X Ray[Title/Abstract])) OR (X-Ray CAT Scan*[Title/Abstract])) OR (Transmission Computed Tomography[Title/Abstract])) OR (Electron Beam Computed Tomography[Title/Abstract])) OR (Electron Beam Tomography[Title/Abstract])) OR (X-Ray Computerized Axial Tomography[Title/Abstract])) OR (CT[Title/Abstract])) OR ((((((((((Ultrasonography[MeSH Terms]) OR (Diagnostic Ultrasound*[Title/Abstract])) OR (Ultrasound Imaging*[Title/Abstract])) OR (Echotomography[Title/Abstract])) OR (Ultrasonic Imaging[Title/Abstract])) OR (Medical Sonography[Title/Abstract])) OR (Ultrasonographic Imaging*[Title/Abstract])) OR (Ultrasonic Diagnoses[Title/Abstract])) OR (Computer Echotomography[Title/Abstract])) OR (Ultrasonic Tomography[Title/Abstract]))) OR (Agatston score[Title/Abstract]) OR (examination[Title/Abstract]) OR (imag*[Title/Abstract])
8	((((((Calcium[MeSH Terms]) OR (Blood Coagulation Factor IV[Title/Abstract])) OR (Coagulation Factor IV[Title/Abstract])) OR (Factor IV, Coagulation[Title/Abstract])) OR (Calcium-40[Title/Abstract])) OR (Calcium 40[Title/Abstract])) OR (Factor IV[Title/Abstract])
9	(((Phosphorus[MeSH Terms]) OR (Phosphates[Title/Abstract])) OR (Phosphorous Acids[Title/Abstract])) OR (Organophosphates[Title/Abstract])
10	(((Calcium, Dietary[MeSH Terms]) OR (Calcium Compounds[MeSH Terms])) OR (Dietary Supplements*[MeSH Terms])) OR (Supplementation*[Title/Abstract]) OR (Calcium Acetate[Title/Abstract]) OR (Calcium Carbonate[Title/Abstract])OR (Calcium Lactate[Title/Abstract])
11	((((((((((((((((Dialysis[MeSH Terms])) OR (Dialyses, Renal[Title/Abstract])) OR (Renal Dialyses[Title/Abstract])) OR (Dialysis, Renal[Title/Abstract])) OR (Hemodialysis[Title/Abstract])) OR (Hemodialyses[Title/Abstract])) OR (Dialysis, Extracorporeal[Title/Abstract])) OR (Dialyses, Extracorporeal[Title/Abstract])) OR (Extracorporeal Dialyses[Title/Abstract])) OR (Extracorporeal Dialysis[Title/Abstract]))

	OR (haemodialysis[Title/Abstract])) OR (haemodialyses[Title/Abstract])) OR (peritoneal dialysis[Title/Abstract])) OR (apd[Title/Abstract])) OR (pd[Title/Abstract])) OR (capd[Title/Abstract])) OR (ccpd[Title/Abstract])
12	((((((((((phosphate binder*[Title/Abstract]) OR (sevelamer[Title/Abstract])) OR (calcium carbonate[Title/Abstract])) OR (lanthanum carbonate[Title/Abstract])) OR (Aluminum Accumulation[Title/Abstract])) OR (Colestilan[Title/Abstract])) OR (calcium-acetate[Title/Abstract])) OR (ferric citrate[Title/Abstract])) OR (sucroferric oxyhydroxide[Title/Abstract])) OR (non-calcium-based binder*[Title/Abstract])) OR (calcium-based binder*[Title/Abstract])) OR (iron-based binder*[Title/Abstract])
13	(Vitamin D[MeSH Terms]) OR (vitamin d[Title/Abstract])
14	(((Parathyroid Hormone[MeSH Terms]) OR (Parathyroid Hormone[Title/Abstract])) OR (iPTH[Title/Abstract])) OR (PTH[Title/Abstract])
15	((Hyperparathyroidism, Secondary[MeSH Terms]) OR (Secondary Hyperparathyroidism*[Title/Abstract])) OR (SHPT[Title/Abstract])
16	((((((((((((((((Vitamin D analogues[Title/Abstract]) OR (vitamin D analogs[Title/Abstract])) OR (paricalcitol[MeSH Terms])) OR (19-nor-1alpha,25-dihydroxyvitamin D2[Title/Abstract])) OR (19-nor-1,25-(OH)2D2[Title/Abstract])) OR (Zemplar[Title/Abstract])) OR (paricalcitol-d6[Title/Abstract])) OR (calcipotriene[MeSH Terms])) OR (1,24(OH)2-22-ene-24-cyclopropyl D3[Title/Abstract])) OR (calcipotriol[Title/Abstract])) OR (Psorcutan[Title/Abstract])) OR (Sorilux[Title/Abstract])) OR (Enstilar[Title/Abstract])) OR (heximar win care[Title/Abstract])) OR (Dovonex[Title/Abstract])) OR (Daivonex[Title/Abstract])) OR (PRI-2205[Title/Abstract])) OR (MC 903[Title/Abstract])) OR (MC-903[Title/Abstract])
17	(((((((((Calcimimetic Agents[MeSH Terms]) OR (calcimimetic[MeSH Terms])) OR (Cinacalcet[MeSH Terms])) OR (Cinacalcet Hydrochloride[Title/Abstract])) OR (Alpha-methyl-N-(3-(3-(trifluoromethyl)phenyl)propyl)-1-naphthalenemethanamine, (alpha)-hydrochloride[Title/Abstract])) OR (Sensipar[Title/Abstract])) OR (AMG 073[Title/Abstract])) OR (KRN 1493[Title/Abstract])) OR (AMG073[Title/Abstract])) OR (etelcalcetide[Title/Abstract])) OR (evocalcet[Title/Abstract])
18	#1 AND #2 AND #3 AND #4
19	#1 AND #2 AND #3 AND #5
20	#1 AND #2 AND #3 AND #6 AND #7
21	#1 AND #2 AND #3 AND #8
22	#1 AND #2 AND #3 AND #9
23	#1 AND #2 AND #3 AND #10
24	#1 AND #2 AND #3 AND #11
25	#1 AND #2 AND #3 AND #12
26	#1 AND #2 AND #3 AND #13
27	#1 AND #2 AND #3 AND #14
28	#1 AND #3 AND #15 AND #16
29	#1 AND #3 AND #15 AND #17

Appendix B. Evidence Summary Tables

**Table S1**	Summary table of studies evaluating the ability of serum biochemical indexes to diagnose CKD-MBD in children–study characteristics
**Table S2**	Summary table of studies evaluating the ability of serum biochemical indexes to diagnose CKD-MBD in children–study population characteristics
**Table S3**	Summary table of studies evaluating the ability of serum biochemical indexes to diagnose CKD-MBD in children–results
**Table S4**	Summary table of studies evaluating the ability of serum biochemical indexes to diagnose CKD-MBD in children–quality
**Table S5**	Evidence profile of studies evaluating the ability of serum biochemical indexes to diagnose CKD-MBD in children
**Table S6**	Summary table of studies evaluating the ability of biochemical indicators to assess the nature and severity of bone abnormalities in children with CKD-MBD–study characteristics
**Table S7**	Summary table of studies evaluating the ability of biochemical indicators to assess the nature and severity of bone abnormalities in children with CKD-MBD–study population characteristics
**Table S8**	Summary table of studies evaluating the ability of biochemical indicators to assess the nature and severity of bone abnormalities in children with CKD-MBD–results
**Table S9**	Summary table of studies evaluating the ability of biochemical indicators to assess the nature and severity of bone abnormalities in children with CKD-MBD–quality
**Table S10**	Evidence profile of studies evaluating the ability of biochemical indicators to assess the nature and severity of bone abnormalities in children with CKD-MBD
**Table S11**	Summary table of studies evaluating the ability of DXA to assess the bone mineral density and bone mass in children with CKD-MBD–study characteristics
**Table S12**	Summary table of studies evaluating the ability of DXA to assess the bone mineral density and bone mass in children with CKD-MBD–study population characteristics
**Table S13**	Summary table of studies evaluating the ability of DXA to assess the bone mineral density and bone mass in children with CKD-MBD–results
**Table S14**	Summary table of studies evaluating the ability of DXA to assess the bone mineral density and bone mass in children with CKD-MBD–quality
**Table S15**	Evidence profile of studies evaluating the ability of DXA to assess the bone mineral density and bone mass in children with CKD-MBD
**Table S16**	Summary table of studies evaluating the occasion of histological examination to assess the bone abnormalities in children with CKD-MBD–study characteristics
**Table S17**	Summary table of studies evaluating the occasion of histological examination to assess the bone abnormalities in children with CKD-MBD–study population characteristics
**Table S18**	Summary table of studies evaluating the occasion of histological examination to assess the bone abnormalities in children with CKD-MBD–results
**Table S19**	Summary table of studies evaluating the occasion of histological examination to assess the bone abnormalities in children with CKD-MBD–quality
**Table S20**	Evidence profile of studies evaluating the occasion of histological examination to

	assess the bone abnormalities in children with CKD-MBD
**Table S21**	Summary table of studies evaluating the ability of different imaging to detect vascular calcification in children with CKD-MBD–study characteristics
**Table S22**	Summary table of studies evaluating the ability of different imaging to detect vascular calcification in children with CKD-MBD–study population characteristics
**Table S23**	Summary table of studies evaluating the ability of different imaging to detect vascular calcification in children with CKD-MBD–results
**Table S24**	Summary table of studies evaluating the ability of different imaging to detect vascular calcification in children with CKD-MBD–quality
**Table S25**	Evidence profile of studies evaluating the ability of different imaging to detect vascular calcification in children with CKD-MBD
**Table S26**	Summary table of clinical trials examining the treatment of calcium supplementation in CKD G3a-G5D presenting with hypocalcemia–study characteristics
**Table S27**	Summary table of clinical trials examining the treatment of calcium supplementation in CKD G3a-G5D presenting with hypocalcemia–study population characteristics
**Table S28**	Summary table of clinical trials examining the treatment of calcium supplementation in CKD G3a-G5D presenting with hypocalcemia–results
**Table S29**	Summary table of clinical trials examining the treatment of calcium supplementation in CKD G3a-G5D presenting with hypocalcemia–quality
**Table S30**	Evidence profile of randomized controlled trial examining the treatment of calcium supplementation in CKD G3a-G5D presenting with hypocalcemia
**Table S31**	Summary table of clinical trials examining the treatment of hyperphosphatemia with phosphate lowering drugs in CKD G3a-G5 children –study characteristics
**Table S32**	Summary table of clinical trials examining the treatment of hyperphosphatemia with phosphate lowering drugs in CKD G3a-G5 children –study population characteristics
**Table S33**	Summary table of clinical trials examining the treatment of hyperphosphatemia with phosphate lowering drugs in CKD G3a-G5 children –results
**Table S34**	Summary table of clinical trials examining the treatment of hyperphosphatemia with phosphate lowering drugs in CKD G3a-G5 children –quality
**Table S35**	Evidence profile of randomized controlled trial examining the treatment of hyperphosphatemia with phosphate lowering drugs in CKD G3a-G5
**Table S36**	Summary table of clinical trials examining the dialysis regimen of hypocalcemia or hyperphosphatemia in CKD G5D–study characteristics
**Table S37**	Summary table of clinical trials examining the dialysis regimen of hypocalcemia or hyperphosphatemia in CKD G5D–study population characteristics
**Table S38**	Summary table of clinical trials examining the dialysis regimen of hypocalcemia or hyperphosphatemia in CKD G5D–results
**Table S39**	Summary table of clinical trials examining the dialysis regimen of hypocalcemia or hyperphosphatemia in CKD G5D –quality
**Table S40**	Summary table of clinical trials examining the treatment of CKD-MBD with active vitamin D in CKD G3a-G5 –study characteristics
**Table S41**	Summary table of clinical trials examining the treatment of CKD-MBD with active vitamin D in CKD G3a-G5 –study population characteristics

**Table S42**	Summary table of clinical trials examining the treatment of CKD-MBD with active vitamin D in CKD G3a-G5 –results
**Table S43**	Summary table of clinical trials examining the treatment of CKD-MBD with active vitamin D in CKD G3a-G5 –quality
**Table S44**	Evidence profile of randomized controlled trials examining the treatment of CKD-MBD with active vitamin D in CKD G3a-G5
**Table S45**	Summary table of clinical trials examining the treatment of CKD-MBD with vitamin D analogues in CKD G3a-G5D – study characteristics
**Table S46**	Summary table of clinical trials examining the treatment of CKD-MBD with vitamin D analogues in CKD G3a-G5D – study population characteristics
**Table S47**	Summary table of clinical trials examining the treatment of CKD-MBD with vitamin D analogues in CKD G3a-G5D – results
**Table S48**	Summary table of clinical trials examining the treatment of CKD-MBD with vitamin D analogues in CKD G3a-G5D – quality
**Table S49**	Evidence profile of randomized controlled trial examining the treatment of CKD-MBD with vitamin D analogues in CKD G3a-G5D
**Table S50**	Summary table of clinical trials examining the treatment of CKD-MBD with cinacalcet in CKD G3a-G5 –study characteristics
**Table S51**	Summary table of clinical trials examining the treatment of CKD-MBD with cinacalcet in CKD G3a-G5 –study population characteristics
**Table S52**	Summary table of clinical trials examining the treatment of CKD-MBD with cinacalcet in CKD G3a-G5 –results
**Table S53**	Summary table of clinical trials examining the treatment of CKD-MBD with cinacalcet in CKD G3a-G5 –quality
**Table S54**	Evidence profile of randomized controlled trial examining the treatment of CKD-MBD with cinacalcet in CKD G3a-G5

Clinical question 1:
Which serum biochemical indexes can be used to diagnose CKD-MBD in children?

Supplemental table 1. Summary table of studies evaluating the ability of serum biochemical indexes to diagnose CKD-MBD in children–study characteristics

Author, year	Region of study	N	CKD GFR category	Dialysis modality Dialysate calcium	Follow up duration	Funding source	Study type
Christian Lerch 2018 [18]	Europe	80	CKD 2-5	/	12M	European Society for Pediatric Nephrology (reference number ESPN 2014.3), KfH Foundation for Preventive Medicine and ERA-EDTA (to D.H.)	Cohort study
Anke Doyon 2015 [19]	Europe	556	CKD 3-5	/	6y	None	Cohort study

Note: The content of this table is summarized based on references [18, 19] in the main article.

Supplemental table 2. Summary table of studies evaluating the ability of serum biochemical indexes to diagnose CKD-MBD in children–study population characteristics

Author, year	Intervention Group	Age, mean years	Male, %	Race, %	Kidney function Duration on dialysis	Baseline MBD labs (Ca, P, PTH, VitD, ALP et al.)	Bone evaluation technique	DXA score/ Fractures/ calcification
Christian Lerch 2018 [18]	vitamin D	ERGO:<br>9.1(5.1)<br>4C<br>12.7(3.3)	63%	/	/	ERGO:<br>Ca:2.34 (0.14)mmol/l<br>P:1.47(0.22)mmol/l<br>iPTH:4.2 (3.0–5.7)pmol/l;<br>25(OH)D:50.8(18.4)noml/l<br>4C:<br>Ca:2.32 (0.22)mmol/l	/	/

						P:1.6(0.31)mmol/l<br>iPTH:13.2 (8.1–21.2) pmol/l;<br>25(OH)D:46.1(26.0)noml/l		
Anke Doyon 2015 [19]	rhGH	6-18Y	66%	/	/	Ca: 2.21±0.23 mmol/l;<br>P: 1.54±0.37 mmol/l;<br>iPTH: 12.3 （7.1, 20.2）pmol/l;<br>25OHD: 11.0 (6.6, 18.1) ug/l	/	/

Note: MBD = mineral bone disorder; DXA = dual-energy X-ray absorptiometry**.** The content of this table is summarized based on references [18, 19] in the main article.

Supplemental table 3. Summary table of studies evaluating the ability of serum biochemical indexes to diagnose CKD-MBD in children–results

Author, year	Arm 1	Arm 2	Cointerventions	Monitoring program	Outcomes	Results Arm 1 vs. Arm 2	Complications
Christian Lerch 2018 [18]	ERGO:<br>vitamin D (n =20)<br>4C:<br>vitamin D (n =20)	ERGO:<br>Placebo (n =20)<br>4C:<br>Placebo (n =20)	Vitamin D2 supplementation	serum and urinary concentrations of albumin, creatinine, cystatin C, bicarbonate, phosphate and calcium	children in the ERGO study had normal FGF23 (median 0.31 SDS) and BAP (0.10 SDS) but decreased Klotho and sclerostin (0.77 and 1.04 SDS, respectively), whereas 4C patients had increased FGF23 (3.87 SDS), BAP (0.78 SDS) and sclerostin (0.76 SDS) but normal Klotho (0.27 SDS) levels.	Vitamin D supplementation further increased FGF23 in 4C but not in ERGO patients. Serum Klotho and sclerostin normalized with vitamin Dsupplementation in ERGO but remained unchanged in 4C patients. BAP levels were unchanged in all patients.	/
Anke	1) Patients with	1) Matched control	rhGH	Bone alkaline	BAP SDS was positively	Standardized levels of	/

Doyon 2015 [19]	rhGH treatment(n=41)<br>2) rGH treated patients(n=42)<br>3)one follow-up visit available rGH treated (n=38)	group of patients without rGH treatment(n=41)<br>2) rGH untreated patients(n=510)<br>3) rGH untreated (n=474)	therapy	phosphatase (BAP), tartrate-resistant acid phosphatase 5b (TRAP5b), sclerostin and C-terminal FGF-23 (cFGF23)	correlated to TRAP5b SDS (r =0.58,p<0.0001) and inversely to cFGF-23 SDS (r= -0.2,p<0.0001).Sclerostin showed no correlation with TRAP5b, BAP or cFGF-23.	BAP, TRAP5b and cFGF-23 were increased whereas sclerostin was reduced. BAP was correlated positively and cFGF-23 inversely with eGFR. Intact serum parathormone was an independent positive predictor of BAP and TRAP5b and negatively associated with sclerostin. BAP and TRAP5B were negatively affected by increased C-reactive protein levels.	

Note: The content of this table is summarized based on references [18, 19] in the main article.

Supplemental table 4. Summary table of studies evaluating the ability of serum biochemical indexes to diagnose CKD-MBD in children–quality

Author, year	Selection				Comparability		Exposure		
Christian Lerch 2018 [18]	Yes	Yes	No	Yes	Yes	Yes	Yes	Yes	No
Anke Doyon 2015 [19]	Yes	Yes	No	No	Yes	No	Yes	Yes	No

Note: The content of this table is summarized based on references [18, 19] in the main article.

Supplemental table 5. Evidence profile of studies evaluating the ability of serum biochemical indexes to diagnose CKD-MBD in children

Preliminary recommendation	Included studies	Methodological limitations	Relevance	Result consistency	Data adequacy	CERQual general evaluation	Interpretation of evaluation results
1.1 We recommend serum calcium, phosphorus, alkaline phosphatase (ALP), intact parathyroid hormone (iPTH), and 25-hydroxyvitamin D3 (25(OH)D3) as the biochemical indicators for diagnosing CKD-MBD in children with CKD G2-G5D.<br>1.2 We recommend monitoring the above-mentioned biochemical indicators in children with CKD from stage 2, and adjusting the frequency of testing based on the stage of CKD, rate of progression, and medication treatment during the G2-G5D period.	Christian Lerch 2018；Anke Doyon 2015	There are no significant methodological limitations (including 2 studies with high quality).	The relevance is moderate.	The results are relatively consistent.	The data is relatively abundant, including 636 children with CKD.	Moderate	The evidence quality of the two multicenter studies (from multiple European countries) is high, with moderate relevance, and the data is relatively abundant.

Note：The evidence summarized in table S1-S5 directly supports Recommendation 1.1 and Recommendation 1.2 (see Page 22).

Clinical question 3:
Which biochemical indicators can be used to assess the nature and severity of bone abnormalities in children with CKD-MBD?

Supplemental table 6. Summary table of studies evaluating the ability of biochemical indicators to assess the nature and severity of bone abnormalities in children with CKD-MBD–study characteristics

Author, year	Region of study	N	CKD GFR category	Dialysis modality Dialysate calcium	Follow up duration	Funding source	Study type
Orfeas Liangos 2018 [27]	Europe	105	CKD 3-5	HD	6y	None	Retrospective study
Barreto FC 2008 [28]	Brazil	97	CKD 5	HD	1y	None	Cohort study
Syazrah Salam 2018 [29]	Europe	69	CKD 4-5	HD	6m	None	Cross-sectional study
Anke Doyon 2015 [19]	Europe	556	CKD 3-5	/	6y	None	Cohort study

Note: The content of this table is summarized based on references [19, 27-29] in the main article.

Supplemental table 7. Summary table of studies evaluating the ability of biochemical indicators to assess the nature and severity of bone abnormalities in children with CKD-MBD–study population characteristics

Author, year	Intervention Group	Age, mean years	Male, %	Race, %	Kidney function Duration on dialysis	Baseline MBD labs (Ca, P, PTH, VitD, ALP et al.) (the type of assays)	Bone evaluation technique	DXA score/ Fractures/ calcification
Orfeas Liangos 2018 [27]	Dialysis	Normal renal function：59 (11) CKD NOD：	Normal renal function：58%	/	/	Normal renal function：Ca: 2.42（0.13）mmol/l; iPTH: 8.1（8.05）pmol/l; ALP：81（24）U/l	/	/

		69 (14)<br>CKD 5D：<br>74 (10)	CKD NOD：<br>24%<br>CKD 5D：<br>53%			CKD NOD：<br>Ca: 2.46（0.35）mmol/l;<br>iPTH: 15.02 （ 26.31 ） pmol/l;<br>ALP：102（36）U/l<br>CKD 5D：<br>Ca: 2.39（0.49）mmol/l;<br>iPTH: 19.35 （ 16.68 ） pmol/l;<br>ALP：130（109）U/l<br>(Not available)		
Barreto FC 2008 [28]	Dialysis	49.5±13.1	65%	White 58%	36.4±24.8m	Ca: 1.28±0.1mmol/l<br>P: 5.4±1.5 mg/dl<br>iPTH：391.6±389.9 pg/ml (automated immunoassay)	Bone Biopsy	/
Syazrah Salam 2018 [29]	/	62 (12)	77.4%	/	/	CKD:<br>iPTH:188(121–280) pg/ml;<br>Ca: 2.28 (0.15)mmol/l;<br>P:1.53 (0.3)mmol/dl<br>25Hydroxyvitamin D:22.9 (9.4)ng/ml<br>Control:<br>iPTH: 32 (27–45)pg/ml;<br>Ca:2.28 (0.07)mmol/l;<br>P:1.06 (0.15)mmol/dl<br>25Hydroxyvitamin D:23.9	Transiliac Bone Biopsy and Histomorphometry	CKD：<br>Forearm -0.4 (1.5)<br>Total hip -0.2 (1.0)<br>Lumbar spine 0.4 (1.7)<br>Control：<br>Forearm 0.2 (0.4)<br>Total hip 0.6

						(7.0)ng/ml<br>(automated immunoassay)		(1.1)<br>Lumbar spine 0.5(1.6)
Anke Doyon 2015 [19]	rhGH	6-18Y	66%	/	/	Ca: 2.21±0.23 mmol/l;<br>P: 1.54±0.37 mmol/l;<br>iPTH: 12.3（7.1, 20.2）pmol/l;<br>25OHD: 11.0 (6.6, 18.1) ug/l<br>(Not available)	/	/

Note: MBD = mineral bone disorder; DXA = dual-energy X-ray absorptiometry. The content of this table is summarized based on references [19, 27-29] in the main article.

Supplemental table 8. Summary table of studies evaluating the ability of biochemical indicators to assess the nature and severity of bone abnormalities in children with CKD-MBD–results

Author, year	Arm 1	Arm 2	Cointerventions	Monitoring program	Outcomes	Results Arm 1 vs. Arm 2	Complications
Orfeas Liangos 2018 [27]	CKD NOD（n=33）	CKD 5D（n=53）	Dialysis	Demographics, comorbid conditions, laboratory values, medications, kidney function and dialysis data	iPTH was signiffcantly higher in CKD 5D with high-turnover ROD, 26 (18) versus 8 (9) pmol/l (p<0.001). BAP showed no association. In CKD NOD, high-turnover ROD was associated with elevated iPTH, 32 (44) versus 8 (11) pmol/l (p=0.001), and BAP, 39 (32) versus 16 (7) U/l (p=0.01). iPTH achieved receiver operator characteristic (ROC) areas under the	high-turnover ROD was associated with elevated iPTH at a low cutoff but not with BAP. The same diagnosis in CKD NOD was associated both with iPTH and BAP.	/

					curve (AUC) of 0.83 (P=0.003) and 0.91 (P=0.019) for high-turnover ROD among CKD 5D and CKD NOD patients, respectively. An iPTH cutoff of 12.8 (CKD 5D) and 13.5 pmol/l (CKD NOD) reached sensitivities and speciffcities of 0.83, 0.91 and 1.00, 0.91, respectively. In CKD NOD, BAP achieved an AUC of 0.93 (P=0.013) and with a cutoff at 19.8 U/l a sensitivity and speciffcity of 1.00, 0.91, respectively.		
Barreto FC 2008 [28]	high-turnover bone disease (HT) (n=36) low-turnover bone disease (LT) (n=58)	NL (normal histology) (n=3)	Dialysis	Serum ionized calcium，serum phosphorus，iPTH，Bone histomorphometric analysis，ROD	The study regarding age (51.6±13.5 vs 47.5±12.7 years), time on dialysis (34.4±24.6 vs 37.5±25.1 months), serum phosphorus (5.9±1.2 vs 5.8±1.0 mg per 100 ml), serum ionized calcium (1.28±0.09 vs 1.28±0.07 mmol/l), serum iPTH (361.6±337.9 vs 420.4±307.5 pg/ml), and the histological type of bone lesion.	In the group achieving the target levels of iPTH 88% had low turnover. Intact PTH levels less than 150 pg/ml for identifying low turnover and greater than 300 pg /ml for high turnover presented a positive predictive value of 83 and 62%, respectively.	/
Syazrah Salam	CKD(n=69)	Control(n=68)	/	Serum iPTH, bALP, intact	For discriminating low bone turnover, bALP, intact PINP, and TRAP5b had	The biomarkers bALP, intact PINP, and	

2018 [29]				PINP,CTX, TRAP5b, and 25-hydroxyvitamin D	an areas under the receiver operating characteristic curve (AUCs) of 0.82, 0.79, and 0.80, respectively, each significantly better than the iPTH AUC of 0.61. Furthermore, radius HR-pQCT total volumetric bone mineral density and cortical bone volume had AUCs of 0.81 and 0.80, respectively. For discriminating high bone turnover, iPTH had an AUC of 0.76, similar to that of all other biomarkers tested.	TRAP5b and radius HR-pQCT parameters can discriminate low from nonlow bone turnover. Despite poor diagnostic accuracy for low bone turnover, iPTH can discriminate high bone turnover with accuracy similar to that of the other biomarkers, including CTX.	
Anke Doyon 2015 [19]	3) Patients with rhGH treatment(n=41)<br>4) rGH treated patients(n=42)<br>3)one follow-up visit available rGH treated (n=38)	4) Matched control group of patients without rGH treatment(n=41)<br>5) rGH untreated patients(n=510)<br>6) rGH untreated (n=474)	rhGH	Bone alkaline phosphatase (BAP), tartrate-resistant acid phosphatase 5b (TRAP5b), sclerostin and C-terminal FGF-23 (cFGF23)	BAP SDS was positively correlated to TRAP5b SDS (r =0.58,p<0.0001) and inversely to cFGF-23 SDS (r= -0.2,p<0.0001).Sclerostin showed no correlation with TRAP5b, BAP or cFGF-23.	Standardized levels of BAP, TRAP5b and cFGF-23 were increased whereas sclerostin was reduced. BAP was correlated positively and cFGF-23 inversely with eGFR. Intact serum parathormone was an independent positive predictor of BAP and TRAP5b and	

						negatively associated with sclerostin. BAP and TRAP5B were negatively affected by increased C-reactive protein levels.	

Note: The content of this table is summarized based on references [19, 27-29] in the main article.

Supplemental table 9. Summary table of studies evaluating the ability of biochemical indicators to assess the nature and severity of bone abnormalities in children with CKD-MBD–quality

Author, year	Selection				Comparability		Exposure		
Orfeas Liangos 2018 [27]	Yes	Yes	Yes	No	No	No	Yes	No	No
Barreto FC 2008 [28]	Yes	Yes	Yes	No	Yes	No	Yes	Yes	Yes
Syazrah Salam 2018 [29]	No	Yes	Yes	Yes	Yes	Yes	Yes	No	No
Anke Doyon 2015 [19]	Yes	Yes	No	No	Yes	No	Yes	Yes	No

Note: The content of this table is summarized based on references [19, 27-29] in the main article.

Supplemental table 10. Evidence profile of studies evaluating the ability of biochemical indicators to assess the nature and severity of bone abnormalities in children with CKD-MBD

**Preliminary recommendation**	**Included studies**	**Methodological limitations**	**Relevance**	**Result consistency**	**Data adequacy**	**CERQual general evaluation**	**Interpretation of evaluation results**
In children with CKD G3a-G5D, it is recommended to monitor iPTH and BAP together to assess	Orfeas Liangos 2018; Barreto FC 2008;	There are no significant methodologic	All studies are conducted in adults,	The results are relatively consistent.	The data is relatively abundant.	Moderate	The evidence quality of the conclusions from three studies (from Germany,

the nature and severity of bone abnormalities.	Syazrah Salam 2018	al limitations.	reducing one level.				the UK, and Brazil) is high, with moderate relevance and sufficient data.

**Preliminary recommendation**	**Included studies**	**Methodological limitations**	**Relevance**	**Result consistency**	**Data adequacy**	**CERQual general evaluation**	**Interpretation of evaluation results**
Based on the characteristics of children's growth and development and the disease features of pediatric CKD-MBD, measurement of C-terminal FGF23 (cFGF23) may be considered as an early indicator of mineral metabolism disturbances in settings where this assay is available..	Anke Doyon 2015	There are no significant methodological limitations.	Good correlation.	There is only one study.	The data is relatively abundant.	Moderate	There is only one European study with high evidence quality, good relevance, and sufficient data for its conclusion.

Note：The evidence summarized in table S6-S10 directly supports Recommendation 3.1 and Recommendation 3.2 (see Page 26-27).

Clinical question 4:

What are the appropriate imaging techniques for detecting changes in BMD and bone mass in children with CKD-MBD?

Supplemental table 11. Summary table of studies evaluating the ability of DXA to assess the bone mineral density and bone mass in children with CKD-MBD–study characteristics

Author, year	Region of study	N	CKD GFR category	Dialysis modality<br>Dialysate calcium	Follow up duration	Funding source	Study type
Lalayiannis AD 2021 [34]	Europe and USA	103	CKD 4-5	HD(44)<br>HDF(12)<br>Home HD(4)<br>PD(17)	/	joint Kidney Research UK (TF_002_20161124)<br>National Institute for Health Research (NIHR) grant (CDF2016-09-038; Career Development Fellowship)	cross-sectional study
Bakr AM 2004 [35]	Egypt	65	CKD 2-5	/	12M	/	cross-sectional study
Salem N 2023 [36]	Egypt	535	ESRD	HD(93)	2 years	The Science, Technology & Innovation Funding Authority (STDF) in cooperation with The Egyptian Knowledge Bank (EKB)	Case-control observational study

Note: The content of this table is summarized based on references [34-36] in the main article.

Supplemental table 12. Summary table of studies evaluating the ability of DXA to assess the bone mineral density and bone mass in children with CKD-MBD–study population characteristics

Author, year	Interventio n Group	Age, mean years	Male, %	Race, %	Kidney function Duration on dialysis	Baseline MBD labs (Ca, P, PTH, VitD, ALP et al.)	Bone evaluati on techniqu	DXA score/ Fractures/ calcification

							e	
Lalayiannis AD 2021 [34]	Dialysis	11.9 (6.9 to 13.8)	30.8%	Caucasian/Asian/Black/other: 76.9%/7.7%/15.5%/0	3.8 (1.4 to 9.0)	Ca:2.47 (2.41 to 2.52)mmol/l;<br>P:1.46 (1.29 to 1.62)mmol/l;<br>25(OH)D: 94 (71 to141)nmol/l;<br>PTH: 6.90 (3.90 to 17.05)pmol/l	/	/
Bakr AM 2004 [35]	/	3-14.8	76.2%	/	/	Ca: 8.3(6.6-9.1)mg/dl；<br>P: 9.8(5.0-11.9)mg/dl;<br>iPTH:398.1(147-930.6)pg/ml	/	Scores: 2.12±1.4
Salem N 2023 [36]	ESRD on regular HD	Male: 12.89±2.40;Female:13.03±2.86	51.6%	/	on regular HD for at least 6 months	Male:<br>Ca: 7.88±1.12mg/dl；<br>P: 6.70(3.1-13.7)mg/dl;<br>iPTH:542.40(123-3079)ng/L<br>Female:<br>Ca: 8.16±1.09mg/dl；<br>P: 5.60(2.7-13.1)mg/dl;<br>iPTH:465.10(107-2924)ng/L	DXA	Male:<br>LS-aBMD: 0.659±0.14g/cm^2<br>LS-TBS:1.319(1.032-1.423)<br>Female:<br>LS-aBMD: 0.714±0.11g/cm^2<br>LS-TBS:1.309(0.863-1.468)

Note: MBD = mineral bone disorder; DXA = dual-energy X-ray absorptiometry. The content of this table is summarized based on references [34-36] in the main article.

Supplemental table 13. Summary table of studies evaluating the ability of DXA to assess the bone mineral density and bone mass in children with CKD-MBD–results

Author, year	Arm 1	Arm 2	Cointerventions	Monitoring program	Outcomes	Results Arm 1 vs. Arm 2	Complications

Lalayiannis AD 2021 [34]	CKD participants(n=26)	Dialysis participants(n=77)	Dialysis	Serum ionized Ca (iCa), total Ca, P, magnesium (Mg), bicarbonate, intact PTH (iPTH), 25-hydroxyvitamin D [25(OH)D] and ALP	CortBMD was negatively associated with parathyroid hormone (PTH; r=-0.44, P < 0.0001) and alkaline phosphatase (ALP; r=-0.22, P ¼ 0.03) and positively with calcium (Ca; r=-0.33, P ¼ 0.001).At PTH <3 times upper limit of normal, none of the patients had a CortBMD below 2 SD (odds ratio 95% confidence interval 7.331 to infinity).On multivariable linear regression PTH (β=- 0.43 , P < 0.0001), ALP (β=-0.36, P < 0.0001) and Ca (β=0.21, P=0.005) together predicted 57% of variability in CortBMD.	PTH, ALP and Ca, but not DXA, are moderate predictors of cortical BMD.	/
Bakr AM 2004 [35]	pre-dialysis (n=21)	Hemodialysis (n=44)	/	BMD at lumbar spinal region (L2-L4) was measured in all children usingDEXA (DEXA, Lunar DPX-IQ system, USA).	In osteopenic children there was a negative correlation between Z-scores and serum phosphorus (r=0.61, P=0.004), intact parathyroid hormone (iPTH) (r=0.47, P=0.03), and bone-specific alkaline phosphatase (r=0.52, P=0.02) and a positive correlation with total calcium (r=0.41, P=0.07) and 25-hydroxycholecalciferol (r=0.53, P=0.02). Osteopenic children who had iPTH values 200 pg/ml were more	Osteopenia, assessed by DEXA, is frequent in children with CRF. It occurs early irrespective of the duration or the severity of CRF. In children with ESRF the degree of	/

					osteopenic than those who had lower iPTH levels (P=0.006).	osteopenia is correlated with laboratory markers of renal osteodystrophy and patients with biochemical findings of secondary hyperparathyroidism are more osteopenic than the others.	
Salem N 2023 [36]	ESRD on regular HD (n=93)	Healthy controls (n=442)	/	Clinical evaluation; biochemical evaluation: before the dialysis session (mid-week): BMD and TBS measurement, and albumin, calcium (Ca), phosphate(P), PTH and ALP, corrected calcium and (Ca*P) product	aBMD and TBS were significantly lower in short-for-age and normal height-for-age patients compared to the corresponding values of controls (p<0.05 for all). TBSZ-score correlated significantly with aBMDZ-CA (r=0.234; p=0.024) but not with aBMDZ-HA (r=0.077; p=0.462). Patients with history of fractures (5 patients only) had significantly lower TBS scores compared to those without fracture history (p=0.016).	TBS is significantly reduced in children on maintenance HD and is associated with increased fracture incidence.	/

Note: The content of this table is summarized based on references [34-36] in the main article.

Supplemental table 14. Summary table of studies evaluating the ability of DXA to assess the bone mineral density and bone mass in children with CKD-MBD–quality

Author, year	Selection				Comparability		Exposure		
Lalayiannis AD 2021 [34]	Yes	Yes	No	No	Yes	No	Yes	Yes	No
Bakr AM 2004 [35]	Yes	Yes	No	No	No	No	Yes	No	No
Salem N 2023 [36]	Yes	Yes	Yes	Yes	Yes	No	Yes	Yes	No

Note: The content of this table is summarized based on references [34-36] in the main article.

Supplemental table 15. Evidence profile of studies evaluating the ability of DXA to assess the bone mineral density and bone mass in children with CKD-MBD

Preliminary recommendation	Included studies	Methodological limitations	Relevance	Result consistency	Data adequacy	CERQual general evaluation	Interpretation of evaluation results
It is reasonable to consider absorptiometry (DXA) for bone mineral density testing to assess bone quality and fracture risk for children with CKD G3a-G5D.	Lalayiannis AD 2021; Bakr AM 2004; Salem N 2023	There may be serious methodological limitations	The correlation is acceptable.	The results are relatively consistent.	The adequacy of the data is insufficient.	Low	The study primarily comes from Europe, America, and Egypt. The evidence quality of the conclusion's methodology is relatively poor, with acceptable correlation but insufficient data adequacy.

Note：The evidence summarized in table S11-S15 directly supports Recommendation 4.1 (see Page 31).

Clinical question 5:
Is bone histological examination necessary for children with CKD-MBD?

Supplemental table 16. Summary table of studies evaluating the occasion of histological examination to assess the bone abnormalities in children with CKD-MBD–study characteristics

Author, year	Region of study	N	CKD GFR category	Dialysis modality Dialysate calcium	Follow up duration	Funding source	Study type
Bakkaloglu SA 2010 [50]	USA	161	CKD 5	/	12M	None	Retrospective study
Pereira RC 2015 [51]	USA	68	CKD 5	/	/	None	Cross-sectional study
Carvalho CG 2015 [52]	USA	22	CKD 5	Continuous cycling peritoneal dialysis (CCPD)	12M	None	Retrospective study
Soeiro EMD 2020 [53]	Brazil	42	CKD 5	HD(23)<br>PD(19)	2Y	CNPq, Conselho Nacional de Desenvolvimento Científico e Tecnológico (grant numbers 305106/2018-0, 303684/2013-5, and 304249/2013-0, respectively).	Retrospective study
Wesseling-Perry K 2012 [54]	USA	52	CKD 2-5	/	6M	None	Retrospective study

Note: The content of this table is summarized based on references [50-54] in the main article.

Supplemental table 17. Summary table of studies evaluating the occasion of histological examination to assess the bone abnormalities in children with CKD-MBD–study population characteristics

Author, year	Intervention	Age, mean	Male, %	Race, %	Kidney function	Baseline MBD labs (Ca, P,	Bone	DXA score/

	Group	years			Duration on dialysis	PTH, VitD, ALP et al.)	evaluation technique	Fractures/ calcification
Bakkaloglu SA 2010 [50]	Dialysis	14.1±1.2	50.3%	Hispanic 63%<br>Caucasian 23%<br>Caucasian 8%<br>Asian 6%	13±3 m	Ca：9.1±0.6 mg/dl<br>P：8.2±0.6 mg/dl<br>ALP: 212±40 IU/l<br>PTH: 163±48 pg/ml	Bone biopsy	/
Pereira RC 2015 [51]	Dialysis	13.9±0.5	50%	White 48%<br>Hispanic 26%	/	Ca：9.4±0.1 mg/dl<br>P：6.0±0.1 mg/dl<br>ALP: 337±38 IU/l<br>PTH: 736(422, 1063) pg/ ml	Computed tomography (CT) measurements; Transiliac bone biopsy cores	/
Carvalho CG 2015 [52]	PD	10.4±0.7	45%	White 27%<br>Black 4%<br>Asian 4%<br>Hispanic 65%	/	Ca：10.0±0.2 mg/dl<br>P：5.4±0.6 mg/dl<br>ALP: 271±56 IU/l<br>PTH: 122(87, 158) pg/ ml	Bone biopsy	/
Soeiro EMD 2020 [53]	Dialysis	11.3±4.3	71%	/	21(12.0–31.7)m	Ca: 10.2 ± 1.3mg/dl<br>P: 5.3 ± 1.6mg/dl<br>PTH: 145 (46–356) pg/ml<br>ALP:302 (155–393)IU/l	Bone biopsy	Mean BMD Zscore was − 0.33 ± 1.37
Wesseling-Perry K 2012 [54]	/	13.3±4.4	58%	/	/	Ca: 9.5±0.4mg/dl<br>P: 4.7±0.8mg/dl<br>PTH: 52 (42-87) pg/ml<br>ALP:238±82IU/l<br>25(OH)vitamin D: 31.2±9.3	Bone biopsy	Average Z scores for height and weight were -1.33±1.4 and -

						ug/ml		0.61±1.53

Note: MBD = mineral bone disorder; DXA = dual-energy X-ray absorptiometry. The content of this table is summarized based on references [50-54] in the main article.

Supplemental table 18. Summary table of studies evaluating the occasion of histological examination to assess the bone abnormalities in children with CKD-MBD–results

Author, year	Arm 1	Arm 2	Cointerventions	Monitoring program	Outcomes	Results Arm 1 vs. Arm 2	Complications
Bakkaloglu SA 2010 [50]	Low(n=7)<br>High(n=92)	Normal(n=62)	Dialysis	Bone biopsy, serum biochemical variables including calcium, phosphorus, alkaline phosphatase, and PTH	Increased bone turnover and abnormal mineralization were prevalent (57% and 48%, respectively); bone volume was normal or increased in all subjects. Predictive algorithms for different skeletal diagnoses were established by Classification and regression tree analysis. Serum parathyroid hormone (PTH) less than 400 pg/ml in combination with alkaline phosphatase values less than 400 IU/L provided the highest correct prediction rate for patients with both normal bone turnover and normal mineralization	A combination of serum calcium, alkaline phosphatase, and PTH levels may lead to a more precise noninvasive assessment of turnover and mineralization abnormalities in this population	/
Pereira RC 2015 [51]	Normal To High Bone Turnover (n=52)<br>Pure Osteomalacia	Controls (n=14)	Dialysis	Bone biopsy, serum biochemical variables including calcium, phosphorus, alkaline	Bone volume measurements were highly correlated between bone histomorphometry and mCT (bone volume/tissue volume between the two techniques: r=0.70; P,0.001, trabecular	Measures of bone volume can be accurately assessed with mCT. Bone mineral density is lower in patients with excessive osteoid	/

	(n=7)<br>Adynamic Bone (n=9)			phosphatase, and PTH	thickness and trabecular separation: r=0.71; P,0.001, and r=0.56; P,0.001, respectively). Osteoid accumulation as determined by bone histomorphometry correlated inversely with bone mineral density as assessed by mCT (osteoid thickness: r=20.32; P=0.01 and osteoid volume: r=20.28; P=0.05).	accumulation and higherin patients with adynamic, well mineralized bone. Thus, bone mineralization may be accurately assessed by mCT of bone biopsy cores.	
Carvalho CG 2015 [52]	High trabecular bone turnover(n=12)	Normal to low trabecular bone turnover(n=10)	PD	Bone biopsy, calcium, phosphorus, alkaline phosphatase, and PTH	Trabecular bone turnover and osteoid volume correlated with PTH levels (r=0.86, p<0.01, respectively). Internal cortical osteonal bone formation rate was directly related to alkaline phosphatase (r=0.45, p<0.05) and inversely related to IGF-1 values (r= − 0.55, p<0.01) and internal cortical porosity was also related to serum alkaline phosphatase levels (r=0.57, p<0.01).	Secondary hyperparathyroidism was associated with increased external cortical, relative to internal cortical, osteonal activity in pediatric dialysis patients. The clinical consequences of these changes and their response to therapy for secondary hyperparathyroidism remain to be defined.	/
Soeiro EMD 2020 [53]	Low(n=25)<br>High(n=7)	Normal(n=10)	Dialysis	Bone biopsy, calcium, phosphorus, alkaline phosphatase, PTH, and DXA	Low bone turnover was present in 59% of patients, abnormal mineralization in 29%, and low bone volume in 7%. ROC curve analysis showed a fair performance of biomarkers to predict	While PTH and AP were associated with turnover and mineralization, we recognize the limitation of their performance to	/

					TMV status. PTH < 2 times ULN independently associated with low bone turnover (RR 5.62, 95% CI 1.01–31.24; p = 0.049), in a model adjusted for race, calcitriol dosage, and calcium. It was also associated with abnormal mineralization (RR 1.35, 95% CI 1.04–1.75; p = 0.025).	clearly distinguish high from low/normal bone turnover and normal from abnormal mineralization.	
Wesseling-Perry K 2012 [54]	Stage2 CKD(n=14) Stage3 CKD(n=24)	Stage 4/5 CKD (n=14)	/	Blood determinations of creatinine, bicarbonate, calcium, albumin, phosphorus, alkaline phosphatase, 25(OH)vitamin D, and 1,25(OH)2vitamin D, PTH, and FGF-23	Bone turnover was normal in all patients with stage 2, but was increased in 13% with stage 3 and 29% with stage 4/5 CKD. Defective mineralization was present in 29% of patients with stage 2, 42% with stage 3, and 79% with stage 4/5 CKD.	Elevated circulating FGF-23 levels and defects in skeletal mineralization early in the course of CKD suggest that factors other than the traditional markers of mineral deficiency play a crucial role in the development of renal bone disease.	/

Note: The content of this table is summarized based on references [50-54] in the main article.

Supplemental table 19. Summary table of studies evaluating the occasion of histological examination to assess the bone abnormalities in children with CKD-MBD–quality

Author, year	Selection				Comparability		Exposure		
Bakkaloglu SA 2010 [50]	Yes	Yes	No	No	Yes	No	Yes	Yes	No
Pereira RC 2015 [51]	Yes	Yes	Yes	Yes	Yes	No	Yes	Yes	No

Carvalho CG 2015 [52]	Yes	No	No	No	Yes	No	Yes	Yes	No
Soeiro EMD 2020 [53]	Yes	Yes	No	No	Yes	No	Yes	Yes	No
Wesseling-Perry K 2012 [54]	Yes	No	No	No	Yes	No	Yes	Yes	No

Note: The content of this table is summarized based on references [50-54] in the main article.

Supplemental table 20. Evidence profile of studies evaluating the occasion of histological examination to assess the bone abnormalities in children with CKD-MBD

Preliminary recommendation	Included studies	Methodological limitations	Relevance	Result consistency	Data adequacy	CERQual general evaluation	Interpretation of evaluation results
If it is necessary to determine or adjust treatment decisions by understanding the type of renal osteodystrophy, bone biopsy might be performed for children with CKD G3a-G5D.	Bakkaloglu SA 2010, Pereira RC 2015, Carvalho CG 2015, Soeiro EMD 2020, Wesseling-Perry K 2012	All of them may have serious methodological limitations.	The correlation is acceptable. Four articles are related to children, and one is related to adults.	The results are relatively consistent.	The data adequacy is insufficient.	Low	The study primarily comes from the United States and Brazil. The evidence quality of the conclusion's methodology is relatively poor, with acceptable correlation but insufficient data adequacy.

Note：The evidence summarized in table S16-S20 directly supports Recommendation 5.1 (see Page 35).

Clinical question 6:
How to detect vascular calcification in children with CKD-MBD？

Supplemental table 21. Summary table of studies evaluating the ability of different imaging to detect vascular calcification in children with CKD-MBD–study characteristics

Author, year	Region of study	N	CKD GFR category	Dialysis modality<br>Dialysate calcium	Follow up duration	Funding source	Study type
Lalayiannis AD 2021 [64]	UK	100	CKD stage 4 and 5	Dialysis (77)	/	No	longitudinal, multicentre study
Lalayiannis AD 2022 [65]	UK	57	CKD stage 4 and 5	CKD (12)<br>HD (18)<br>HDF (9)<br>Home HD (7)<br>PD (11)	/	ADL is funded by a Joint Kidney Research UK (TF_002_20161124) and Kids Kidney Research Training Fellowship grant (KKR/Paed2017/01). RS is funded by National Institute for Health Research (CDF-2016-09-038; Career Development Fellowship) for this research project.	longitudinal, multicentre study

Note: The content of this table is summarized based on references [64, 65] in the main article.

Supplemental table 22. Summary table of studies evaluating the ability of different imaging to detect vascular calcification in children with CKD-MBD–study population characteristics

Author, year	Intervention Group	Age, mean years	Male, %	Race, %	Kidney function Duration on dialysis	Baseline MBD labs (Ca, P, PTH, VitD, ALP et al.)	vascular calcification evaluation technique	DXA score/ Fractures

Lalayiannis AD 2021 [64]	cardiac computed tomography	13.82 (10.68-16.46)	56	Caucasian 52; Asian 27; Black 20; Other 1	eGFR <30 mL/min/1.73m^2 or on dialysis	Total Ca 2.47 (2.37, 2.56)mmol/l, P 1.53 (1.30, 1.87)mmol/l, PTH 3ULN (1ULN, 10ULN), VitD 78.00(37.55, 113.80)nmol/l,	cardiac CT, cIMT, cfPWV, echocardiography	/
Lalayiannis AD 2022 [65]	cardiac computed tomography	15.84(12.56-21.69)	59.65	Caucasian 47.37; Asian 29.82; Black 21.05; Other 1.75	Dialysis vintage: 3.64 (0.58-5.57)	/	cardiac CT, cIMT, cfPWV, echocardiography	LS BMAD -0.6 (-2.04, 0.28), Trabecular BMD z-score -0.38 (-1.47, 0.58), Cortical BMD z-score -1.13 (-2.76, -0.13)

Note: MBD = mineral bone disorder; DXA = dual-energy X-ray absorptiometry. The content of this table is summarized based on references [64, 65] in the main article.

Supplemental table 23. Summary table of studies evaluating the ability of different imaging to detect vascular calcification in children with CKD-MBD–results

Author, year	Arm 1	Arm 2	Cointerventions	Monitoring program	Outcomes	Results Arm 1 vs. Arm 2	Complications
Lalayiannis AD 2021 [64]	CKD (23)	Dialysis (77)	/	cardiac CT, cIMT, cfPWV, echocardiography	CAC (n=10): CKD (n=1), dialysis (n=9); Agatston score range: CKD 4.3(0, 6.4), dialysis 12(0, 412.6);	The cIMT z-score was elevated [median 2.17 (interquartile range 1.14–2.86)] and 10 (10%) had CAC. There	/

					cIMT z-score: CKD 2.46(1.04, 2.76), dialysis 2.01(1.14, 2.94);<br>cfPWV z-score: CKD 0.61(-0.78, 2.23), dialysis 1.52(0.8, 2.81)	was no difference in the prevalence of structural abnormalities in CKD or dialysis cohorts, but functional abnormalities were more prevalent in patients on dialysis (P < 0.05).	
Lalayiannis AD 2022 [65]	Baseline (57)	Follow-up (57)	/	cardiac CT, cIMT, cfPWV, echocardiography	At baseline:<br>Agatston score range: mean 8.10±SD 55.20;<br>cIMT z-score: 1.55 (0.93, 2.66);<br>PWV z-score: 1.08 (-0.42, 2.24);<br>At follow-up:<br>Agatston score range: mean 42.61±SD 123.50;<br>cIMT z-score: 2.03 (1.23, 2.97);<br>PWV z-score: 1.26 (0.25, 2.55);	Children and young adults with CKD or on dialysis may develop vascular calcification even as their BMD increases.	/

Note: The content of this table is summarized based on references [64, 65] in the main article.

Supplemental table 24. Summary table of studies evaluating the ability of different imaging to detect vascular calcification in children with CKD-MBD–quality

Author, year	Selection				Comparability		Exposure		

Lalayiannis AD 2021 [64]	Yes	Yes	No	No	Yes	No	Yes	Yes	No
Lalayiannis AD 2022 [65]	Yes	Yes	No	No	Yes	No	Yes	Yes	Yes

Note: The content of this table is summarized based on references [64, 65] in the main article.

Supplemental table 25. Evidence profile of studies evaluating the ability of different imaging to detect vascular calcification in children with CKD-MBD

Preliminary recommendation	Included studies	Methodological limitations	Relevance	Result consistency	Data adequacy	CERQual general evaluation	Interpretation of evaluation results
For selected children with CKD G3a-G5D at high risk for cardiovascular complications, CT examination may be considered to assess coronary artery and abdominal aortic calcification when the results are expected to meaningfully influence clinical management. However, CT is not recommended for routine screening due to radiation exposure concerns.	Lalayiannis AD 2021, Lalayiannis AD 2022	All of them may have serious methodological limitations.	The correlation is acceptable.	The results are relatively consistent.	The data adequacy is insufficient.	Low	The studies comes from the UK. The evidence quality of the conclusion's methodology is relatively poor, with acceptable correlation but insufficient data adequacy.

Note：The evidence summarized in table S21-S25 directly supports Recommendation 6.1 (see Page 37).

Clinical question 9:
What is the appropriate calcium supplementation strategy for hypocalcemia in children with CKD G3a-G5D?

Supplemental table 26. Summary table of clinical trials examining the treatment of calcium supplementation in CKD G3a-G5D presenting with hypocalcemia–study characteristics

Author, year	Region of study	N	CKD GFR category	Follow up duration	Funding source	Type of study
Qunibi W,2011 [84]	USA	110	CKD4-5(GFR<30mL/min/1.73m2)	12 weeks	Fresenius Medical Care North America, Waltham, MA	RCT
Phelps KR,2002 [85]	USA	18	CCr10-70ml/min	24 weeks(n=14); 16 weeks(n=4)	The National Kidney Foundation of Northeast New York	RCT
Gulati S,2005 [83]	India	100	/	1.5±0.07 years	The Intramural grant programme of the Sanjay Gandhi Post Graduate Institute of Medical Sciences, India	Prospective study

Note: The Gulati S,2005 study was done in idiopathic nephrotic syndrome (INS) and treated with prednisone. The content of this table is summarized based on references [83-85] in the main article.

Supplemental table 27. Summary table of clinical trials examining the treatment of calcium supplementation in CKD G3a-G5D presenting with hypocalcemia–study population characteristics

Author, year	Intervention Group	Age, mean years	Male, %	Race, %	Kidney function Duration on dialysis	Baseline MBD labs (Ca, P, PTH, VitD, ALP et al.)	Bone evaluation technique	DXA score/ Fractures/ calcification
Qunibi W,2011 [84]	Calcium acetate (N = 46); Placebo (N = 64)	Calcium acetate：63.2±11.7；Placebo：	Calcium acetate：50；Placebo：	Calcium acetate(white)：71.7；Placebo(white)：	/	Serum calcium, corrected (mg/dL): Calcium acetate: 9.1±0.7;	/	/

		62.2±14.2	45.3	84.4		Placebo: 9.1±0.6		
Phelps KR,2002 [85]	Group 1(The low-dose group) (N = 8);<br>Group 2(The high-dose group) (N = 10)	Group 1 : 71.3±8.6 (63–83);<br>Group 2 : 69.4±7.3 (55–75)	/	/	/	Serum Ca, mg/dl:<br>Group 1: 9.1±0.4 (8.6–9.6);<br>Group 2: 9.2±0.5(8.4–9.7)	Dual-energy X-ray absorptiometry(DEXA)	BMD
Gulati S,2005 [83]	calcium carbonate:73<br>control:15<br>excluded:12	9.03±0.45	75/88	/	/	Serum Ca (mEq/l):8.5±0.10	Dual energy X-linked absorptiometry (DEXA)	DXA score

Note: MBD = mineral bone disorder; DXA = dual-energy X-ray absorptiometry. The content of this table is summarized based on references [83-85] in the main article.

Supplemental table 28. Summary table of clinical trials examining the treatment of calcium supplementation in CKD G3a-G5D presenting with hypocalcemia–results

Author, year	Arm 1	Arm 2	Cointerventions	Outcomes	Results
Qunibi W,2011 [84]	1 gelcap per meal (P: 4.5-5.0 mg/dL)<br>2 gelcaps per meal (P: 5.1-6.0mg/dL)<br>3 gelcaps per meal (P: > 6.0 mg/dL)	Using placebo	/	After 12 weeks;<br>Arm1: hypocalcemia 5.4%, hypercalcemia 13.5%;<br>Arm2: hypocalcemia 19.5%, hypercalcemia 0%.	Calcium acetate is efficacious and safe in controlling serum phosphorus, calcium, iPTH and serum bicarbonate in non-dialyzed CKD patients.
Phelps KR,2002 [85]	calcium: 1 tablet containing 667 mg of calcium acetate (or 169	calcium: 2 of the tablets with breakfast, 3 with lunch,	/	After 24 weeks(n=14);<br>After 16 weeks(n=4);	Patients treated with high-dose calcium acetate can reduced

	mg of elemental calcium) with each meal.	and 4 with supper.		Arm1: no metabolic or skeletal effect.<br>Arm2: The mean serum calcium concentration rose by a maximum of 7.2%; the BMD increased at L1, L3, and L4.	parathyroid hormone and 1,25-dihy-droxyvitamin D concentrations and increased lumbar BMD in men with preterminal chronic renal failure.
Gulati S,2005 [83]	prescribed calcium (500mg/day) and vitamin D3(200IU/day) supplements	Without calcium and vitamin D3	Steroid	The majority (n=54) had improved BMD at the spine, and another 25 children had stable BMD.<br>Children who were on these supplements (n=73) had a significantly improved z score as compared with those who did not receive them (n=15) (P=0.008).<br>The mean spinal BMD values: 0.607±0.013g/cm2.	Calcium and vitamin D supplements may help in improving BMD in children with INS.

Note: The content of this table is summarized based on references [83-85] in the main article.

Supplemental table 29. Summary table of clinical trials examining the treatment of calcium supplementation in CKD G3a-G5D presenting with hypocal cemia–quality

Author, year	Random sequence generation	Allocation concealment	Blinding of participants and personnel	Blinding of outcome assessors	Incomplete outcome data	Selective reporting	Other bias
Qunibi 2011 [84]	High risk	Uncertain	Low risk	Low risk	High risk	Low risk	High risk
Phelps 2002 [85]	Uncertain	High risk	High risk	Low risk	High risk	Uncertain	Low risk

Author, year	Selection				Comparability		Exposure		
Gulati 2005 [83]	No	Yes	Yes	No	Yes	Yes	Yes	Yes	Yes

Note: The content of this table is summarized based on references [83-85] in the main article.

Supplemental table 30. Evidence profile of randomized controlled trial examining the treatment of calcium supplementation in CKD G3a-G5D presenting with hypocalcemia

1. Question: Calcium acetate compared to placebo for CKD-MBD

Certainty assessment							№ of patients		Effect		Certainty	Importance
№ of studies	Study design	Risk of bias	Inconsistency	Indirectness	Imprecision	Other considerations	calcium acetate	placebo	Relative (95% CI)	Absolute (95% CI)		
Effect on calcium												
1	randomised trials	serious[a]	not serious	serious[b]	not serious	none	46	64	-	MD **0.7 higher** (0.34 higher to 1.06 higher)	⊕⊕◯◯ Low	CRITICAL
Effect on phosphorus												

Certainty assessment							№ of patients		Effect		Certainty	Importance
№ of studies	Study design	Risk of bias	Inconsistency	Indirectness	Imprecision	Other considerations	calcium acetate	placebo	Relative (95% CI)	Absolute (95% CI)		
1	randomised trials	serious[a]	not serious	serious[b]	not serious	none	46	64	-	MD **0.7 lower** (1.28 lower to 0.12 lower)	⨁⨁◯◯ Low	CRITICAL
Effect on iPTH												
1	randomised trials	serious[a]	not serious	serious[b]	not serious	none	46	64	-	MD **201 lower** (303.7 lower to 98.3 lower)	⨁⨁◯◯ Low	CRITICAL
Hypercalcium												

Certainty assessment							№ of patients		Effect		Certainty	Importance
№ of studies	Study design	Risk of bias	Inconsistency	Indirectness	Imprecision	Other considerations	calcium acetate	placebo	Relative (95% CI)	Absolute (95% CI)		
1	randomised trials	serious[a]	not serious	serious[b]	not serious	very strong association	5/37 (13.5%)	0/41 (0.0%)	**OR 14.05** (0.75 to 263.38)	**0 fewer per 1,000** (from 0 fewer to 0 fewer)	⊕⊕⊕⊕ High	CRITICAL

CI: confidence interval; **MD:** mean difference; **OR:** odds ratio

Explanations

a. The study was high risk in terms of radomization process, missing outcome data and other bias.

b. The study was conducted on adults.

2. **Question:** Calcium acetate compared to diet for CKD-MBD

Certainty assessment							№ of patients		Effect		Certainty	Importance
№ of studies	Study design	Risk of bias	Inconsistency	Indirectness	Imprecision	Other considerations	calcium acetate	diet	Relative (95% CI)	Absolute (95% CI)		
Effect on calcium												

Certainty assessment							№ of patients		Effect		Certainty	Importance
№ of studies	Study design	Risk of bias	Inconsistency	Indirectness	Imprecision	Other considerations	calcium acetate	diet	Relative (95% CI)	Absolute (95% CI)		
1	randomised trials	serious[a]	not serious	serious[b]	not serious	none	35	35	-	MD **0.1 higher** (0.18 lower to 0.38 higher)	⊕⊕◯◯ Low	CRITICAL

Effect on phosphorus

1	randomised trials	serious[a]	not serious	serious[b]	not serious	none	35	35	-	MD **0.4 lower** (0.77 lower to 0.03 lower)	⊕⊕◯◯ Low	CRITICAL

Effect on PTH

Certainty assessment							№ of patients		Effect		Certainty	Importance
№ of studies	Study design	Risk of bias	Inconsistency	Indirectness	Imprecision	Other considerations	calcium acetate	diet	Relative (95% CI)	Absolute (95% CI)		
1	randomised trials	serious[a]	not serious	serious[b]	not serious	none	35	35	-	MD **49.65 lower** (93.62 lower to 5.68 lower)	⊕⊕◯◯ Low	CRITICAL

CI: confidence interval; **MD:** mean difference

Explanations

a. The study was high risk in terms of performance bias and other bias.
b. The study was conducted on adults.

Note：The evidence summarized in table S26-S30 directly supports Recommendation 9.1 (see Page 46).

Clinical question 10:

How to choose phosphate-lowering drugs for children with CKD G3a-5D who develop hyperphosphatemia?

Supplemental table 31. Summary table of clinical trials examining the treatment of hyperphosphatemia with phosphate lowering drugs in CKD G3a-G5 children – study characteristics

Author, year	Region of study	N	CKD GFR category	Dialysis modality<br>Dialysate calcium	Follow up duration	Funding source	Type of study
Toida 2012 [88]	Japan	50	ESRD	HD (50)	7 months	/	RCT
Sprague 2009 [89]	USA	121	CKD G3-4	/	8 weeks	Shire Pharmaceuticals	RCT
Caglar 2008 [92]	Turkey	50	CKD G4	/	8 weeks	/	RCT

Note: The content of this table is summarized based on references [88, 89, 92] in the main article.

Supplemental table 32. Summary table of clinical trials examining the treatment of hyperphosphatemia with phosphate lowering drugs in CKD G3a-G5 children – study population characteristics

Author, year	Intervention Group	Age, mean years	Male, %	Race, %	Kidney function<br>Duration on dialysis	Baseline MBD labs (Ca, P, PTH, VitD)	Bone evaluation technique
Toida 2012 [88]	Lanthanum carbonate (LC)	LC: 65.2 ± 13.8<br>CaC:65.9 ± 8.9	LC: 60%<br>CaC: 60%	/	HD<br>LC: 7.1 ± 6.8 (Y)<br>Control: 6.5 ± 5.2 (Y)	①Serum phosphate (mg/dl)<br>LC: 7.76 ± 2.14<br>CaC:7.30 ± 1.86<br>②Serum corrected calcium (mg/dl)<br>LC:8.62 ± 0.71<br>CaC:8.67 ± 0.82<br>③Serum intact PTH (pg/ml)	Serum FGF23, BAP, TRAP5b

						LC:328.5 ± 254.1<br>CaC:234.9 ± 184.7	
Sprague 2009 [89]	Lanthanum carbonate (LC)	LC: 61.8 (12.9)<br>Placebo: 63.0 (12.7)	LC: 51.3%<br>Placebo: 51.2%	LC:<br>White (75.6%),black or African American (19.2%), other (5.1%);<br>Placebo:<br>White (80.5%), black or African American (17.1%), other (2.4%);	/	①Serum phosphate (mg/dl):<br>LC 5.28 ± 0.09,<br>Placebo 5.38 ± 0.12;<br>②serum iPTH(pg/ml): LC 183.5 ± 19.5.<br>Placebo 179.3 ± 24.4;<br>③serum calcium(mg/dl):<br>LC 8.86 ± 0.07,<br>Placebo 8.97 ± 0.09.	/
Caglar 2008 [92]	Sevelamer	Sevelamer :43.1 ± 12.6<br>CaC: 43.6 ± 13.6	/	/	/	①iPTH (pg/ml)<br>Sevelamer: 155 (120 to 183),<br>CaC: 148 (148 to 186);<br>②Serum Ca (mg/dl)<br>Sevelamer: 8.1 ± 0.4<br>CaC: 8.0 ± 0.4<br>③Serum PO4 (mg/dl)<br>Sevelamer:7.8±0.6<br>CaC: 7.8±0.7<br>④Ca×PO4 product<br>Sevelamer: 62 (54 to 73)<br>CaC: 63 (46 to 71)	/

Note: MBD = mineral bone disorder; DXA = dual-energy X-ray absorptiometry. The content of this table is summarized based on references [88, 89, 92] in the main article.

Supplemental table 33. Summary table of clinical trials examining the treatment of hyperphosphatemia with phosphate lowering drugs in CKD G3a-G5 children – results

Author, year	Arm1	Arm2	Cointerventions	Monitoring program	Outcomes	Results Arm 1 vs. Arm 2	complications
Toida 2012 [88]	receiving LC 750 mg/d for 3 months, then were switched to the alternative binder for the subsequent 3 months	Receiving CAC 1500 mg/d for 3 months, then were switched to the alternative binder for the subsequent 3 months	vitamin D analogues	Serum levels of P and Ca were measured every 2 weeks.Serum levels of iPTH, BAP, and TRAP5b were measured after each washout period and after treatment. Serum FGF23 levels were determined with an enzyme-linked immunosorbent assay (ELISA) kit.	①the serum P level in both groups was significantly decreased: LC: 5.6 ± 1.3 mg/dl, CaC: 5.6 ± 1.2 mg/dl; ②cCa was significantly increased in both groups. ③the Ca×P product showed a significant decrease: LC: 49.6±11.8 mg/ dl^2, CaC: 51.1±11.2 mg / dl^2; ④the mean change of iPTH from baseline to after treatment was significantly larger in the CaC group compared with the LC group (–73.9 ± 78.7 vs. –12.9 ± 80.2 pg/ml, p < 0.001).	LC effectively reduced the serum P level (like CaC) and allowed an increase in the dosage of vitamin D analogue therapy without hypercalcemia in HD patients.	Hypercalcemia over10.5 mg/ dl was observed only in patients taking CaC (LC vs. CaC groups, n = 0 vs. 2).
Sprague 2009 [89]	LC	Placebo	Calcium, vitamin D compounds	Biochemical and hematologic parameters were measured. Serum Urine specimens were collected at baseline, week 4, and week 8. Adverse events (AEs) and	①Serum phosphate (mg/dl) had decreased from baseline by 0.55 ± 0.10 and 0.18 ± 0.13 in LC and placebo groups. ②serum iPTH(pg/ml): decreased 23.8 ± 8.6 and 8.8 ± 11.0 in LC and placebo groups.	LC is an effective phosphate binder in patients with CKD G3-4, with a safety	AEs were experienced by 47.4% of patients in the lanthanum carbonate

				serious adverse events (SAEs) were recorded.	③serum calcium(mg/dl): increased 0.12 ± 0.05 and -0.09 ± 0.07 in LC and placebo group.	profile and tolerability similar to that of placebo.	group compared with 61.0% in the placebo group.
Caglar 2008 [92]	Average dose of sevelamer: 4800mg/d	Starting dose of CaC: 300mg/d	/	During the study period, serum calcium and phosphorus concentration were measured every 2 wk.	After treatment<br>①iPTH (pg/ml)<br>Sevelamer: 164 (117 to 232)<br>CaC: 176 (123 to 253)<br>②Serum Ca (mg/dl)<br>Sevelamer:7.9 ± 0.5<br>CaC: 8.2 ± 0.3<br>③Serum PO4 (mg/dl)<br>Sevelamer: 5.9 ± 0.9<br>CaC: 6.0 ± 0.7	Short-term sevelamer treatment significantly increases fetuin-A levels and improves FMD in nondiabetic stage 4 CKD patients.	Sevelamer:abdominal pain, nausea, muscle cramps.<br>CaC: hypercalcemic

Note: The content of this table is summarized based on references [88, 89, 92] in the main article.

Supplemental table 34. Summary table of clinical trials examining the treatment of hyperphosphatemia with phosphate lowering drugs in CKD G3a-G5 children – quality

Author, year	Random sequence generation	Allocation concealment	Blinding of participants and personnel	Blinding of outcome assessors	Incomplete outcome data	Selective reporting	Other bias

Toida 2012 [88]	Low risk	Uncertain	High risk	Low risk	High risk	Uncertain	Uncertain
Sprague 2009 [89]	Uncertain	Uncertain	Low risk	Uncertain	High risk	High risk	High risk
Caglar 2008 [92]	Uncertain	Uncertain	High risk	High risk	High risk	High risk	Low risk

Note: The content of this table is summarized based on references [88, 89, 92] in the main article.

AMSTAR Checklist:

	Question 1	Question 2	Question 3	Question 4	Question 5	Question 6	Question 7	Question 8	Question 9	Question 10	Question 11
Navaneethan2011 [87]	Yes	Yes	Yes	Yes	Yes	Yes	Yes	Yes	Yes	Yes	Yes
Zhai 2014 [90]	No	Yes	Yes	Yes	No	Yes	Yes	Yes	Yes	Yes	Yes
Hahn D2015 [91]	Yes	Yes	Yes	Yes	Yes	Yes	Yes	Yes	Yes	Yes	Yes

Note: The content of this table is summarized based on references [87, 90, 91] in the main article.

Supplemental table 35. Evidence profile of randomized controlled trial examining the treatment of hyperphosphatemia with phosphate lowering drugs in CKD G3a-G5

1. Question: Lanthanum carbonate compared to Calcium carbonate for hyperphosphatemia in CKD-MBD

Certainty assessment							№ of patients		Effect		Certainty	Importance
№ of studies	Study design	Risk of bias	Inconsistency	Indirectness	Imprecision	Other considerations	Lanthanum carbonate	Calcium carbonate	Relative (95% CI)	Absolute (95% CI)		
**Effect on phosphorus**												

Certainty assessment							№ of patients		Effect		Certainty	Importance
№ of studies	Study design	Risk of bias	Inconsistency	Indirectness	Imprecision	Other considerations	Lanthanum carbonate	Calcium carbonate	Relative (95% CI)	Absolute (95% CI)		
1	randomised trials	not serious	not serious	serious[a]	not serious	none	25	25	-	MD **0** (0.69 lower to 0.69 higher)	⊕⊕⊕◯ Moderate	CRITICAL
**Effect on PTH**												
1	randomised trials	not serious	not serious	serious[a]	not serious	none	25	25	-	MD **38.2 higher** (68.82 lower to 145.22 higher)	⊕⊕⊕◯ Moderate	CRITICAL
**Hypercalcemia**												

Certainty assessment							№ of patients		Effect		Certainty	Importance
**№ of studies**	**Study design**	**Risk of bias**	**Inconsistency**	**Indirectness**	**Imprecision**	**Other considerations**	**Lanthanum carbonate**	**Calcium carbonate**	**Relative (95% CI)**	**Absolute (95% CI)**		
1	randomised trials	not serious	not serious	serious[a]	not serious	none	0/25 (0.0%)	2/25 (8.0%)	**OR 0.18** (0.01 to 4.04)	**65 fewer per 1,000** (from 79 fewer to 180 more)	⨁⨁⨁◯ Moderate	CRITICAL

CI: confidence interval; **MD:** mean difference; **OR:** odds ratio

Explanations

a. The participants of study were adults.

2. Question: Sevelamer compared to Calcium salts for hyperphosphatemia in CKD-MBD

Certainty assessment							№ of patients		Effect		Certainty	Importance
**№ of studies**	**Study design**	**Risk of bias**	**Inconsistency**	**Indirectness**	**Imprecision**	**Other considerations**	**Sevelamer**	**Calcium salts**	**Relative (95% CI)**	**Absolute (95% CI)**		
**Effect on serum phosphorus**												
16	randomised trials	not serious	serious[a]	not serious	not serious	none	1593	1533	-	MD **0.23 higher** (0.04 higher to 0.42 higher)	⊕⊕⊕◯ Moderate	CRITICAL
**Effect on iPTH**												
12	randomised trials	not serious	not serious	not serious	not serious	none	546	534	-	MD **59.74 higher** (27.47 higher to 92.02 higher)	⊕⊕⊕⊕ High	CRITICAL
**Hypercalcemia**												

Certainty assessment							№ of patients		Effect		Certainty	Importance
№ of studies	Study design	Risk of bias	Inconsistency	Indirectness	Imprecision	Other considerations	Sevelamer	Calcium salts	Relative (95% CI)	Absolute (95% CI)		
12	randomised trials	not serious	not serious	not serious	not serious	none	61/597 (10.2%)	139/547 (25.4%)	**RR 0.45** (0.35 to 0.59)	**140 fewer per 1,000** (from 165 fewer to 104 fewer)	⨁⨁⨁⨁ High	CRITICAL

CI: confidence interval; **MD:** mean difference; **RR:** risk ratio

Explanations

a. I2=57%.

3. Question: Lanthanum carbonate compared to Placebo for hyperphosphatemia in CKD-MBD

Certainty assessment							№ of patients		Effect		Certainty	Importance
№ of studies	Study design	Risk of bias	Inconsistency	Indirectness	Imprecision	Other considerations	Lanthanum carbonate	Placebo	Relative (95% CI)	Absolute (95% CI)		
**Effect on phosphrus**												
1	randomised trials	serious[a]	not serious	serious[b]	not serious	publication bias strongly suspected[c]	78	41	-	MD **0.37 higher** (0.32 higher to 0.42 higher)	⊕◯◯◯ Very low	CRITICAL
**Effect on PTH**												
1	randomised trials	serious[a]	not serious	serious[b]	not serious	publication bias strongly suspected[c]	78	41	-	MD **15 higher** (11.13 higher to 18.87 higher)	⊕◯◯◯ Very low	CRITICAL
**Effect on calcium**												

Certainty assessment							№ of patients		Effect		Certainty	Importance
№ of studies	Study design	Risk of bias	Inconsistency	Indirectness	Imprecision	Other considerations	Lanthanum carbonate	Placebo	Relative (95% CI)	Absolute (95% CI)		
1	randomised trials	serious[a]	not serious	serious[b]	not serious	publication bias strongly suspected[c]	78	41	-	MD **0.21 higher** (0.19 higher to 0.23 higher)	⊕◯◯◯ Very low	CRITICAL

CI: confidence interval; **MD:** mean difference

Explanations

a. According to the result of Rob, most information of the study was high-risk or uncertain.
b. The participants of study were adults.
c. The study was funded by Shire Pharmaceuticals.

4. Question: Iron-based phosphate binders compared to Placebo for hyperphosphatemia in CKD-MBD

Certainty assessment							№ of patients		Effect		Certainty	Importance
№ of studies	Study design	Risk of bias	Inconsistency	Indirectness	Imprecision	Other considerations	Iron-based phosphate binders	Placebo	Relative (95% CI)	Absolute (95% CI)		
**Effect on serum phosphorus**												
3	randomised trials	not serious	serious[a]	not serious	not serious	none	201	72	-	MD **2.43 lower** (3.18 lower to 1.68 lower)	⊕⊕⊕◯ Moderate	CRITICAL
**Serum iron**												
2	randomised trials	not serious	not serious	not serious	not serious	none	159	52	-	MD **9.39 higher** (1.48 higher to 17.3 higher)	⊕⊕⊕⊕ High	CRITICAL

CI: confidence interval; **MD:** mean difference

Explanations

a. I2=67%。

5. Question: Iron-based phosphate binders compared to Sevelamer for hyperphosphatemia in CKD-MBD

Certainty assessment							№ of patients		Effect		Certainty	Importance
№ of studies	Study design	Risk of bias	Inconsistency	Indirectness	Imprecision	Other considerations	Iron-based phosphate binders	Sevelamer	Relative (95% CI)	Absolute (95% CI)		
**Effect on serum phosphorus**												
3	randomised trials	not serious	serious[a]	not serious	not serious	none	935	481	-	MD **0.04 higher** (0.29 lower to 0.36 higher)	⨁⨁⨁◯ Moderate	CRITICAL

CI: confidence interval; **MD:** mean difference

Explanations

a. I2=74%.

6. Question: Sevelamer compared to Calcium-containing phosphate binders for hyperphosphatemia in CKD-MBD of children

Certainty assessment							№ of patients		Effect		Certainty	Importance
№ of studies	Study design	Risk of bias	Inconsistency	Indirectness	Imprecision	Other considerations	Sevelamer	Calcium-containing phosphate binders	Relative (95% CI)	Absolute (95% CI)		
**Effect on phosphorus**												
2	randomised trials	not serious	not serious	not serious	not serious	none	25	23	-	MD **0.17 higher** (0.37 lower to 0.71 higher)	⊕⊕⊕⊕ High	CRITICAL
**Effect on PTH**												
2	randomised trials	not serious	not serious	not serious	not serious	none	25	23	-	MD **51.92 higher** (77.53 lower to 181.36 higher)	⊕⊕⊕⊕ High	CRITICAL
**Effect on calcium**												

Certainty assessment							№ of patients		Effect		Certainty	Importance
**№ of studies**	**Study design**	**Risk of bias**	**Inconsistency**	**Indirectness**	**Imprecision**	**Other considerations**	**Sevelamer**	**Calcium-containing phosphate binders**	**Relative (95% CI)**	**Absolute (95% CI)**		
2	randomised trials	not serious	not serious	not serious	not serious	none	25	23	-	MD **0.4 lower** (1.16 lower to 0.36 higher)	⊕⊕⊕⊕ High	CRITICAL

CI: confidence interval; **MD:** mean difference

7. Question: Sevelamer compared to Calcium acetate for hyperphosphatemia in CKD-MBD

Certainty assessment							№ of patients		Effect		Certainty	Importance
**№ of studies**	**Study design**	**Risk of bias**	**Inconsistency**	**Indirectness**	**Imprecision**	**Other considerations**	**Sevelamer**	**Calcium acetate**	**Relative (95% CI)**	**Absolute (95% CI)**		
**Effect on phosphorus**												

Certainty assessment							№ of patients		Effect		Certainty	Importance
№ of studies	Study design	Risk of bias	Inconsistency	Indirectness	Imprecision	Other considerations	Sevelamer	Calcium acetate	Relative (95% CI)	Absolute (95% CI)		
1	observational studies	serious[a]	not serious	serious[b]	not serious	none	25	25	-	MD **0.1 lower** (0.55 lower to 0.35 higher)	⊕◯◯◯ Very low	CRITICAL
**Effect on PTH**												
1	observational studies	serious[a]	not serious	serious[b]	not serious	none	25	25	-	MD **13.08 lower** (66.57 lower to 40.41 higher)	⊕◯◯◯ Very low	CRITICAL
**Hypercalcemia**												

Certainty assessment							№ of patients		Effect		Certainty	Importance
**№ of studies**	**Study design**	**Risk of bias**	**Inconsistency**	**Indirectness**	**Imprecision**	**Other considerations**	**Sevelamer**	**Calcium acetate**	**Relative (95% CI)**	**Absolute (95% CI)**		
1	observational studies	serious[a]	not serious	serious[b]	not serious	none	0/25 (0.0%)	3/25 (12.0%)	**OR 0.13** (0.01 to 2.58)	**103 fewer per 1,000** (from 119 fewer to 140 more)	⨁◯◯◯ Very low	CRITICAL

CI: confidence interval; **MD:** mean difference; **OR:** odds ratio

Explanations

a. The participants of study was not typical.
b. The participants of study was adults.

8. Question: Sevelamer compared to Calcium-containing phosphate binders for hyperphosphatemia in CKD-MBD

Certainty assessment							№ of patients		Effect		Certainty	Importance
№ of studies	Study design	Risk of bias	Inconsistency	Indirectness	Imprecision	Other considerations	Sevelamer	Calcium-containing phosphate binders	Relative (95% CI)	Absolute (95% CI)		
**Hypercalcemia**												
19	randomised trials	not serious	serious[a]	not serious	not serious	none	73/1578 (4.6%)	282/1509 (18.7%)	**RR 0.27** (0.17 to 0.42)	**136 fewer per 1,000** (from 155 fewer to 108 fewer)	⨁⨁⨁◯ Moderate	CRITICAL
**Hospitalization**												

Certainty assessment							№ of patients		Effect		Certainty	Importance
№ of studies	Study design	Risk of bias	Inconsistency	Indirectness	Imprecision	Other considerations	Sevelamer	Calcium-containing phosphate binders	Relative (95% CI)	Absolute (95% CI)		
5	randomised trials	not serious	serious[b]	not serious	not serious	none	113/493 (22.9%)	245/499 (49.1%)	**RR 0.50** (0.31 to 0.81)	**245 fewer per 1,000** (from 339 fewer to 93 fewer)	⊕⊕⊕◯ Moderate	CRITICAL

CI: confidence interval; **RR:** risk ratio

Explanations

a. I2=63%.
b. I2=85%.

Note：The evidence summarized in table S31-S35 directly supports Recommendation 10.1 and Recommendation 10.2 (see Page 50).

Clinical question 11:
How to adjust the dialysis regimen for hypocalcemia or hyperphosphatemia in children with CKD G5D?

Supplemental table 36. Summary table of clinical trials examining the dialysis regimen of hypocalcemia or hyperphosphatemia in CKD G5D–study characteristics

Author, year	Region of study	N	CKD GFR category	Dialysis modality<br>Dialysate calcium	Follow up duration	Funding source	Type of study
Hoppe 2011 [111]	Germany	16	ESRD	Nocturnal HD (Three dialysis sessions per week, each lasting 8 hours)<br>AND Conventional HD<br><br>calcium concentration: 1.75 mmol/L	0.5-42months	/	open prospective study
Fischbach 2004 [112]	France	5	All had oligoanuric renal residual functions.	from standard on-line haemodiafifiltration<br>(4h, three times/week)<br>to daily on-line haemodiafifiltration<br>(3h, six times/week).	12months	/	single-centre, observational, prospective, non-randomized

Note: The content of this table is summarized based on references [111, 112] in the main article.

Supplemental table 37. Summary table of clinical trials examining the dialysis regimen of hypocalcemia or hyperphosphatemia in CKD G5D–study population characteristics

Author, year	Intervention Group	Age, mean years	Male, %	Race, %	Kidney function Duration on	Baseline MBD labs (P, Ca, PTH, VitD, ALP et al.)	Bone evaluation	DXA score/ Fractures/

					dialysis		technique	calcification
Hoppe 2011 [111]	Nocturnal HD AND Conventional HD	15.1<br>0.5-17	11/16	/	/	Baseline of NHD (patients before entering the NHD<br>Program were undergoing CHD)<br>P(mmol/L): Median 2.14 （Range1.00-3.62）<br>Calcium (mmol/L) : Median2.41 （Range1.24-2.77）<br>PTH (ng/L):445 (Range117-1771）<br>Kt/V: 1.74 （Range 1.6-1.8）<br>Baseline of CHD: NO	/	/
Fischbach 2004 [112]	Before-After Study in the Same Patient.	13.8±3.2	2/5	/	/	P1.87± 0.23mmol/L<br>IPTH 87± 29 pg/ml<br>Kt/V 1.4±0.3	/	/

Note: The content of this table is summarized based on references [111, 112] in the main article.

Supplemental table 38. Summary table of clinical trials examining the dialysis regimen of hypocalcemia or hyperphosphatemia in CKD G5D–results

Author, year	Arm 1	Arm 2	Cointerventions	Outcomes (P, Ca, PTH, VitD, ALP et al.)	Results Arm 1 vs. Arm 2
Hoppe 2011 [111]	Nocturnal HD	Conventional HD<br><br>(Data from patients on conventional HD matched for age, sex, and weight were obtained.)	/	P(mmol/L)<br>（NHD）1.37 （Range 0.46-3.69）<br>（CHD）1.81 （Range 0.9-3.69)<br>Ca(mmol/L)<br>（NHD）2.34 （Range 1.48-3.35）<br>（CHD）2.50 （Range 1.2-3.91)<br>PTH (ng/L)<br>（NHD）184（Range15-1084）	a strong decrease of serum phosphate in group NHD

				（CHD）217（Range 14-1072)<br>Kt/V<br>（NHD）2.15 （Range 1.21-3.16）<br>（CHD）1.44 （Range 1.00-1.91）	
Fischbach 2004 [112]	daily on-line haemodiafifiltration	/	/	（daily on-line haemodiafifiltration）<br>P(mmol/l)6months:1.43±0.22<br>12months:1.28±0.29<br>IPTH (pg/ml)6months:135±47<br>12months:141±32<br>Kt/V 6months: 1.2 ± 0.8 12months:1.3± 0.4;	plasma phosphorus decreased markedly (from 1.87 ± 0.23 to 1.43 ± 0.22 and 1.28 ± 0.29 mmol/l at 6 and 12 months)

Note: The content of this table is summarized based on references [111, 112] in the main article.

Supplemental table 39. Summary table of clinical trials examining the dialysis regimen of hypocalcemia or hyperphosphatemia in CKD G5D –quality

Author, year	Selection				Comparability		Exposure		
Hoppe 2011 [111]	No	No	Yes	No	No	No	Yes	Yes	Yes
Fischbach 2004 [112]	Yes	No	No	No	No	No	Yes	No	No

Note: The content of this table is summarized based on references [111, 112] in the main article.

Note：The evidence summarized in table S36-S39 directly supports Recommendation 11.2 (see Page 54-55).

Clinical question 14:

How to supplement active vitamin D and vitamin D analogues in the children with CKD G3a-G5D when they have SHPT?

Supplemental table 40. Summary table of clinical trials examining the treatment of CKD-MBD with active vitamin D in CKD G3a-G5 –study characteristics

Author, year	Region of study	N	CKD GFR category	Dialysis modality Dialysate calcium	Follow up duration	Funding source	Type of study
Greenbaum 2005 [120]	USA Poland	47	ESRD	/	12 weeks	/	RCT
Ardissino 2000 [122]	Europe	59	Ccr <75 ml/min per 1.73 m2	/	8 weeks	Associazione per il Bambino Nefropatico	RCT
Schmitt 2003 [121]	Europe	24	Ccr <40 ml/min per 1.73 m2	/	12 months	Associazione per il BambinoNefropatico	RCT

Note: The content of this table is summarized based on references [120-122] in the main article.

Supplemental table 41. Summary table of clinical trials examining the treatment of CKD-MBD with active vitamin D in CKD G3a-G5 –study population characteristics

Author, year	Intervention Group	Age, mean years	Male, %	Race, %	Kidney function Duration on dialysis	Baseline MBD labs (Ca, P, PTH, VitD)	Bone evaluation technique
Greenbaum 2005 [120]	Calcitriol N=21 Placebo N=26	15.3±2.8（9.1-12.8） 14.0±3.8（3.4-18.9）	67% 65%	Black 57%,50% White10%，35% Asian/ Pacific islander 5%,0% Other 29%,15%	/	PTH 821±94 pg/mL 952±102 pg/mL	/
Ardissino 2000 [122]	the daily group (n=29) the intermittent group	8.4±4.7 years	76.3%	/	/	①Ca(mg/dl): Daily 9.6±0.5, Intermittent 9.8±0.6; ②P(mg/dl): Daily 5.0±1.3, Intermittent:5.3±0.8; ③ALP(UI/l):	/

	(n=30)					Daily 317(138–1350), Intermittent 351 (127–1361); ④PTH(pg/ml): Daily 485 (range 83–2032), Intermittent 315 (range 93–1638); ⑤Ccr: Daily 22.0+10.1, Intermittent 22.8+13.0	
Schmitt 2003 [121]	the daily group (n=12)<br>the intermittent group (n=12)	Daily 5.5 (2.4–8.4)<br>intermittent 5.1 (1.4–9.1) years	87.5%	/	/	①Ccr: 20±9 ml/min per 1.73 m^2, ②Ca(mg/dl): Daily 9.68±0.33, Intermittent 9.88±0.82; ③P(mg/dl): Daily 5.14±1.21, Intermittent 5.15±0.70; ④ALP(UI/l): Daily 317 (138–771), Intermittent 502 (182–1361) ; ⑤PTH(pg/ml): Daily 567 (114–1209) , Intermittent 332(93–614); ⑥Height −1.72 SDS(−3.83 -0.27)	/

Note: The content of this table is summarized based on references [120-122] in the main article.

Supplemental table 42. Summary table of clinical trials examining the treatment of CKD-MBD with active vitamin D in CKD G3a-G5 –results

Author, year	Arm 1	Arm 2	Cointerventions	Monitoring program	Outcomes	Side effect	Results Arm 1 vs. Arm 2
Greenbaum 2005 [120]	IV calcitriol:<br>3 times/week<br><500 pg/mL<br>0.50 mg<br>500 to 1000 pg/mL<br>1.00 mg<br>>1000 pg/mL	IV placebo:<br>3 times/week	HD and calcium-containing phosphate binders (calcium carbonate or	1 week	Two consecutive 30% decreases in mean PTH levels: Calcitriol group:52%, placebo group: 19%	①elevated Ca×P (>75 mg^2/dL^2): Calcitriol group: 38%, placebo group: 4%; ②hyperphosphatemia (P>6.5): Calcitriol group:71%, placebo group: 46%;	IV calcitriol, at initial doses of 0.5–1.5 mg, effectively reduces PTH levels in pediatric HD patients and that patients should be closely monitored for

	1.50 mg		calcium acetate)			③Hypercalcemia (>10.5 mg/dL): Calcitriol group: 23.8%, placebo group: 0%<br>④overall incidence of treatment-emergent adverse events: Calcitriol group: 85%,<br>placebo group: 62%	hyperphosphatemia and elevated Ca×P product.
Ardissino 2000 [122]	oral calcitriol (10 ng/kg per day)	intermittent oral calcitriol (35 ng/kg given twice a week)	/	4 weeks	①PTH: the daily group 232pg/ml (range 63–1614), the intermittent group<br>218pg/ml (range 2–1785)<br>②Ccr: intermittent group 22.1±13.3ml/min per 1.73 m^2, daily group 21.6±11.0ml/min per 1.73 m^2	One episode of hypercalcemia (>11.5 mg/dl) was observed in both groups and a<br>single episode of hyperphosphatemia (>7.5 mg/dl) was<br>observed in the daily group.	It is concluded that oral calcitriol pulse therapy does not control secondary hyperparathyroidism more effectively than the daily administration of calcitriol in children with chronic renal failure prior to dialysis.
Schmitt 2003 [121]	oral calcitriol (10 ng/kg per day)	intermittent oral calcitriol (35 ng/kg given twice a week)	/	2 months	①Ca(mg/dl): Daily 10.0±0.38, Intermittent 9.9±0.57;<br>②P(mg/dl): Daily 5.5±1.07, Intermittent 5.6±0.94;<br>③ALP(UI/l): Daily 261 (171–562), Intermittent	Five episodes of calcium phosphate product≥70 occurred<br>in the daily group and four in the intermittent group.	Daily and intermittent C do not differentially affect growth rate and are equally effective in controlling secondary hyperparathyroidism in children with chronic renal failure.

					483 (163–1,299);<br>④PTH (ng/ml): the daily group 255 (85–710), the intermittent 179 (51–443);<br>⑤ΔSDS: Daily –0.18±0.34, intermittent –0.05±0.52		

Note: The content of this table is summarized based on references [120-122] in the main article.

Supplemental table 43. Summary table of clinical trials examining the treatment of CKD-MBD with active vitamin D in CKD G3a-G5 –quality

Author, year	Random sequence generation	Allocation concealment	Blinding of participants and personnel	Blinding of outcome assessment	Incomplete outcome data	Selective reporting	Other bias
Greenbaum 2005 [120]	Uncertain	Uncertain	High risk	High risk	Low risk	Low risk	Low risk
Ardissino 2000 [122]	Uncertain	High risk	High risk	High risk	Low risk	Uncertain	Uncertain
Schmitt 2003 [121]	High risk	High risk	High risk	High risk	Low risk	Uncertain	Low risk

Note: The content of this table is summarized based on references [120-122] in the main article.

Supplemental table 44. Evidence profile of randomized controlled trials examining the treatment of CKD-MBD with active vitamin D in CKD G3a-G5

1. Question: Calcitriol compared to Placebo for CKD-MBD with SHPT in children

Certainty assessment							№ of patients		Effect		Certainty	Importance
№ of studies	Study design	Risk of bias	Inconsistency	Indirectness	Imprecision	Other considerations	Calcitriol	Placebo	Relative (95% CI)	Absolute (95% CI)		
**Effect on PTH**												
1	randomised trials	not serious	not serious	not serious	serious[a]	none	11/21 (52.4%)	5/26 (19.2%)	**RR 2.72** (1.12 to 6.61)	**331 more per 1,000** (from 23 more to 1,000 more)	⊕⊕⊕◯ Moderate	CRITICAL
**Hypercalcemia**												
1	randomised trials	not serious	serious[b]	not serious	serious[a]	none	5/21 (23.8%)	0/26 (0.0%)	**RR 13.50** (0.79 to 231.02)	**0 fewer per 1,000** (from 0 fewer to 0 fewer)	⊕⊕◯◯ Low	CRITICAL
**Hyperphosphatemia**												

Certainty assessment							№ of patients		Effect		Certainty	Importance
№ of studies	Study design	Risk of bias	Inconsistency	Indirectness	Imprecision	Other considerations	Calcitriol	Placebo	Relative (95% CI)	Absolute (95% CI)		
1	randomised trials	not serious	not serious	not serious	serious[a]	none	15/21 (71.4%)	12/26 (46.2%)	**RR 1.55** (0.94 to 2.54)	**254 more per 1,000** (from 28 fewer to 711 more)	⊕⊕⊕◯ Moderate	CRITICAL

CI: confidence interval; **RR:** risk ratio

Explanations

a. The sample size was small.
b. The 95% confidence interval is wide (0.79-231.02).

2. **Question:** Daily oral calcitriol compared to intermittent oral calcitrol for CKD-MBD with SHPT in children

Certainty assessment							№ of patients		Effect		Certainty	Importance
№ of studies	Study design	Risk of bias	Inconsistency	Indirectness	Imprecision	Other considerations	Daily oral calcitriol	intermittent oral calcitrol	Relative (95% CI)	Absolute (95% CI)		
**Effect on PTH**												
2	randomised trials	not serious	not serious	not serious	serious[a]	none	41	42	-	MD **192.15 pg/ml higher** (44.4 lower to 398.73 higher)	⊕⊕⊕◯ Moderate	
**Effect on Calcium**												
2	randomised trials	not serious	not serious	not serious	serious[a]	none	41	42	-	MD **0.2 mg/dl lower** (0.45 lower to 0.05 higher)	⊕⊕⊕◯ Moderate	
**Effect on phosphorus**												

Certainty assessment							№ of patients		Effect		Certainty	Importance
№ of studies	Study design	Risk of bias	Inconsistency	Indirectness	Imprecision	Other considerations	Daily oral calcitriol	intermittent oral calcitrol	Relative (95% CI)	Absolute (95% CI)		
2	randomised trials	not serious	not serious	not serious	serious[a]	none	41	42	-	MD **2 mg/dl lower** (0.66 lower to 0.25 higher)	⊕⊕⊕◯ Moderate	

CI: confidence interval; **MD:** mean difference

Explanations

a. The sample size was small.

Note：The evidence summarized in table S40-S44 directly supports Recommendation 14.1 and Recommendation 14.2 (see Page 62).

Supplemental table 45. Summary table of clinical trials examining the treatment of CKD-MBD with vitamin D analogues in CKD G3a-G5D – study characteristics

Author, year	Region of study	N	CKD GFR category	Dialysis modality<br>Dialysate calcium	Follow up duration	Funding source	Type of study
Ala-Houhala 1995 [128]	Finland	22	20±3ml/min/1.73m^2	/	12M	/	prospective study
Hisano 1990 [129]	Japan	12	CAPD	CAPD<br>1.5% or 2.5% dextrose Dianeal solution containing calcium (3.5 meq/L)	12-18M	/	prospective study

Note: The content of this table is summarized based on references [128, 129] in the main article.

Supplemental table 46. Summary table of clinical trials examining the treatment of CKD-MBD with vitamin D analogues in CKD G3a-G5D – study population characteristics

Author, year	Intervention Group	Age, mean years	Male, %	Race, %	Kidney function Duration on dialysis	Baseline MBD labs (Ca, P, PTH, VitD, ALP et al.)	Bone evaluation technique	DXA score/ Fractures/ calcification
Ala-Houhala 1995 [128]	Alphacalcidol	5.6Y (1M-14Y)	16/22	/	/	PTH 398±81ng/l; Ca 2.47±0.16; P 1.69±0.08; ALP 667±52U/L;	/	/
Hisano 1990 [129]	1 alpha vitamin D3 (1a - D3)	2-16Y (mean 9.75Y)	3/12	/	/	n-PTH 3.3±2.4ng/ml; Ca 8.7±0.9 mg/dl; P 6.7±1.8mg/dl; ALP 402±319U/L;	Radiographs of hands, wrists, and knees	Ca precipitated on the conjunctiva bulbi: 1 patient

Note: MBD = mineral bone disorder; DXA = dual-energy X-ray absorptiometry. The content of this table is summarized based on references [128, 129] in the main article.

Supplemental table 47. Summary table of clinical trials examining the treatment of CKD-MBD with vitamin D analogues in CKD G3a-G5D – results

Author, year	Arm 1	Arm 2	Cointerventions	Monitoring program	Outcomes	Results Arm 1 vs. Arm 2	Complications
Ala-Houhala 1995 [128]	Alphacalcidol(0.5-3.0ug) oral thrice weekly	/	Calcium carbonate (0.6-9.0 g/day) for hyperphosphatemia, hypocalcemia and acidosis	At the beginning-2 weeks; and later every 4 weeks for 6-30M	GFR: 21±6 ml/min/1.73m^2<br>6M: PTH 188±69; ALP 664±137;<br>12M: PTH 122±34; ALP 591±38;	We concluded that feedback regulation of PTH with oral alphacalcidol pulse therapy is effective in the treatment of hyperparathyroidism in children with renal failure prior to dialysis.	/
Hisano 1990 [129]	1 alpha vitamin D3 (1a - D3): 0.01 - 0.02 μg/ kg	/	Prednisolone: 10 patients<br>calcium carbonate (0.1 - 0.15 g/kg): 12 patients	Blood chemistries: monthly intervals;<br>n-PTH: at the beginning and end of the study;<br>Radiographs and Heights: at the beginning and end of the study;	n-PTH 1.3±1.4ng/ml; Ca 10.5±0.9 mg/dl; P 4.8±1.4mg/dl; ALP 226±117U/L;	Our results indicate that adequate doses of la - D3 and calcium carbonate are effective in the prevention of ROD and rickets in patients on CAPD.	/

Note: The content of this table is summarized based on references [128, 129] in the main article.

Supplemental table 48. Summary table of clinical trials examining the treatment of CKD-MBD with vitamin D analogues in CKD G3a-G5D – quality

AMSTAR Checklist:

	Question 1	Question 2	Question 3	Question 4	Question 5	Question 6	Question 7	Question 8	Question 9	Question 10	Question 11
Fazel 2020 [130]	Yes	Yes	Yes	Yes	No	Yes	Yes	Yes	Yes	Yes	Yes

Author, year	Selection				Comparability		Exposure		
Ala-Houhala 1995 [128]	Yes	No	No	No	No	No	Yes	No	No
Hisano 1990 [129]	Yes	No	No	No	No	No	Yes	No	No

Note: The content of this table is summarized based on references [128-130] in the main article.

Supplemental table 49. Evidence profile of randomized controlled trial examining the treatment of CKD-MBD with vitamin D analogues in CKD G3a-G5D

Question: Paricacitol compared to Control group or standard treatment for CKD-MBD with SHPT

Certainty assessment							№ of patients		Effect		Certainty	Importance
№ of studies	Study design	Risk of bias	Inconsistency	Indirectness	Imprecision	Other considerations	Paricacitol	Control group or standard treatment	Relative (95% CI)	Absolute (95% CI)		
**Effect on PTH**												
3	randomised trials	not serious	not serious	not serious	serious[a]	none	33/53 (62.3%)	16/50 (32.0%)	**OR 0.12** (0.05 to 0.29)	**267 fewer per 1,000** (from 297 fewer to 200 fewer)	⊕⊕⊕◯ Moderate	CRITICAL

Certainty assessment							№ of patients		Effect		Certainty	Importance
**№ of studies**	**Study design**	**Risk of bias**	**Inconsistency**	**Indirectness**	**Imprecision**	**Other considerations**	**Paricacitol**	**Control group or standard treatment**	**Relative (95% CI)**	**Absolute (95% CI)**		
**Effect on Calcium**												
3	randomised trials	not serious	not serious	not serious	serious[a]	none	-/53	-/50	**RR 1.16** (0.48 to 2.80)	**0 fewer per 1,000** (from 0 fewer to 0 fewer)	⊕⊕⊕◯ Moderate	CRITICAL
**Effect on Phosphorus**												
3	randomised trials	not serious	not serious	not serious	serious[a]	none	-/53	-/50	**RR 0.87** (0.38 to 1.99)	**0 fewer per 1,000** (from 0 fewer to 0 fewer)	⊕⊕⊕◯ Moderate	CRITICAL
**Effect on CaXP**												

Certainty assessment							№ of patients		Effect		Certainty	Importance
**№ of studies**	**Study design**	**Risk of bias**	**Inconsistency**	**Indirectness**	**Imprecision**	**Other considerations**	**Paricacitol**	**Control group or standard treatment**	**Relative (95% CI)**	**Absolute (95% CI)**		
3	randomised trials	not serious	not serious	not serious	serious[a]	none	-/53	-/50	**RR 0.48** (0.15 to 1.50)	**0 fewer per 1,000** (from 0 fewer to 0 fewer)	⨁⨁⨁◯ Moderate	CRITICAL

CI: confidence interval; **OR:** odds ratio; **RR:** risk ratio

Explanations

a. The total sample size was small.

Note：The evidence summarized in table S45-S49 directly supports Recommendation 14.3 (see Page 62).

Clinical question 15:
How to supplement calcimimetics in the children with CKD G3a-G5D when they have SHPT?

Supplemental table 50. Summary table of clinical trials examining the treatment of CKD-MBD with cinacalcet in CKD G3a-G5 –study characteristics

Author, year	Region of study	N	CKD GFR category	Dialysis modality<br>Dialysate calcium	Follow up duration	Funding source	Study type
Warady 2019 [136]	USA and Europe	43	ESRD	HD（27）；PD（16）<br>Mean(SD): 2.66(0.43)	14M	Amgen Inc.	RCT
Alharthi 2015 [137]	Kingdom of Saudi Arabia	28	CKD 4-5	CKD 4：6<br>CKD 5 on HD: 6<br>CKD 5 on APD: 16	24M	None	Prospective cohort study
Joseph 2019 [138]	USA	18	ESRD	HD（5）；PD（13）	6M	None	Retrospective study

Note: The content of this table is summarized based on references [136-138] in the main article.

Supplemental table 51. Summary table of clinical trials examining the treatment of CKD-MBD with cinacalcet in CKD G3a-G5 –study population characteristics

Author, year	Intervention Group	Age, mean years	Male, %	Race, %	Kidney function Duration on dialysis	Baseline MBD labs (Ca, P, PTH, VitD, ALP et al.)	Bone evaluation technique	DXA score/ Fractures/ calcification
Warady 2019 [136]	cinacalcet	13.2Y	49%	72% white	HD: 19.3(22.3)M<br>PD: 26.6(20.3)M	PTH: 776.0(484.8)pg/ml;<br>Ca: 9.90(0.58)mg/dl；<br>P：6.53（1.63）mg/dl；<br>CaxP: 64.50(17.12)	/	/
Alharthi 2015 [137]	cinacalcet	9M-14Y	10/28	/	>6M	iPTH: 1931.76±794.62 pg/ml;<br>Ca: 2.45±0.07 mmol/l;<br>P: 1.97±0.16 mmol/l;<br>ALP: 1173.2±514.1U/L	/	/

Joseph 2019 [138]	cinacalcet	2.3Y(8M-4.5Y)	9/9	Hispanic (7), Caucasian (6), AfricanAmrican (1), Asian (1), mixed (3)	14.4M （2.76-55.2M）	iPTH: 871(344-1870) pg/ml; Ca: 10.6(9.3-12.6) mg/dl; P: 6(4-9.8) mg/dl;	/	/

Note: MBD = mineral bone disorder; DXA = dual-energy X-ray absorptiometry. The content of this table is summarized based on references [136-138] in the main article.

Supplemental table 52. Summary table of clinical trials examining the treatment of CKD-MBD with cinacalcet in CKD G3a-G5 –results

Author, year	Arm 1	Arm 2	Cointerventions	Monitoring program	Outcomes	Results Arm 1 vs. Arm 2	Complications
Warady 2019 [136]	Cinacalcet (n =22) double-blind phase：mean 1.54mg/kg/d, =50.4mg/d; open-label phase: mean 0.77mg/kg/d, =34.6mg/d	Placebo (n =21)	vitamin D sterols (calcitriol and its analogs), calcium supplements, and phosphate binders	Blood samples were collected every 2 weeks and after dose adjustments to measure iPTH. Total serum calcium, phosphorus, and albumin concentrations were measured 1 week before each visit	1.Primary endpoint(mean iPTH decrease ≥ 30%): (P=0.017, 95%CI: 9-62%) Cinacalcet: 12(54.5%) Placebo: 4(19%) 2.Secondary endpoints: ①mean iPTH value ≤ 300 pg/Ml: (95%CI: -23-30%) Cinacalcet: 27%; Placebo: 24%; ②percentage change in corrected total Ca: (95%CI: -8.6-1.3) Cinacalcet: -4.6; Placebo: -1.0; P: (95%CI: -21.0-8.2); Cinacalcet: 2.9; Placebo: 9.3; Ca × P: (95%CI: -22.5-2.6)	Efficacy and safety outcomes observed with cinacalcet in children with SHPT on dialysis were consistent with adult observations, suggesting cinacalcet may meet an unmet medical need for this population.	Hypocalcemia: Cinacalcet 5(22.7%), Placebo 5(19.0%)

					Cinacalcet: -2.0; Placebo: 8.0; ionized Ca: (95%CI: -9.4-7.9) Cinacalcet: -2.3; Placebo:-1.5; ③growth velocity(0-30W): (95%CI: -3.1-3.6) Cinacalcet: 3.3cm/Y(0.8-5.8) Placebo: 3.1cm/Y(0.7-5.6)		
Alharthi 2015 [137]	Cinacalcet: 0.5mg/kg/d 6 patients (CKD 4); 0.5-1 mg/kg/d 16 patients (CKD 5 on APD); 1-1.5mg/kg/d 6 patients (CKD 5 on HD)	/	alfacalcidol, P binders, and Ca supplements	s.BUN, s.Cr, s.Ca, s.P, s.iPTH, CaxP product, Vit D, s.Alk.P, GFR, 24h urinary proteins, u.albumin, u.Ca, u.P, u.Cr: monthly; renal ultrasound and echocardiography : every 3 months	iPTH: 354.25±274.15 pg/ml (P <0.001); Ca: 2.41±0.13 mmol/l (P =0.157); P: 1.39±0.29 mmol/l (P =0.207); ALP: 395.6±123.0 U/L(P <0.001)	All patients showed at least a 60% reduction in iPTH (60%–97%). Cinacalcet use was proven safe for all pediatric and adolescent patients with CKD-4/5 during the study period, and at the same time most of the patients reached the suggested iPTH target values.	no symptomatic hypocalcemia, hypophosphatemia, or other adverse side effects
Joseph 2019 [138]	Cinacalcet: mean dosage (initiation): 6.2±2.8mg/d	/	phosphate binders	Monthly: Serum Ca, serum P, growth parameters,	iPTH: 386(140-710) pg/ml; Ca: 10.1(9.9-12.5) mg/dl; P: 4.6(4-5.6) mg/dl;	Median iPTH declined by 37% (IQR 11 – 72) at 1 month,	Hypocalcemia

	mean dosage (1 month): 7.5±3.2mg/d<br>mean dosage (6 month): 8.6±5.3mg/d			Every 3 months: 25-OH VD;<br>Quarterly: PTH		42% (IQR –27 – 76) at<br>3 months, and 51% (IQR 19 – 89) at 6 months.	

Note: The content of this table is summarized based on references [136-138] in the main article.

Supplemental table 53. Summary table of clinical trials examining the treatment of CKD-MBD with cinacalcet in CKD G3a-G5 –quality

Author, year	Random sequence generation	Allocation concealment	Blinding of participants and personnel	Blinding of outcome assessors	Incomplete outcome data	Selective reporting	Other bias
Warady 2019 [136]	Uncertain	Low risk	Low risk	Low risk	High risk	Low risk	High risk

Author, year	Selection				Comparability		Exposure		
Alharthi2015 [137]	Yes	Yes	No	No	No	No	Yes	Yes	Yes
Joseph2019 [138]	Yes	Yes	No	No	No	No	Yes	No	No

Note: The content of this table is summarized based on references [136-138] in the main article.

Supplemental table 54. Evidence profile of randomized controlled trial examining the treatment of CKD-MBD with cinacalcet in CKD G3a-G5

Question: Cinacalcet compared to Placebo for CKD-MBD with SHPT

Certainty assessment							№ of patients		Effect		Certainty	Importance
№ of studies	Study design	Risk of bias	Inconsistency	Indirectness	Imprecision	Other considerations	Cinacalcet	Placebo	Relative (95% CI)	Absolute (95% CI)		
**Effect on PTH**												
1	randomised trials	serious[a]	not serious	not serious	serious[b]	none	12/22 (54.5%)	4/21 (19.0%)	**OR 4.26** (0.99 to 18.30)	**310 more per 1,000** (from 2 fewer to 621 more)	⨁⨁◯◯ Low	CRITICAL
**Percentage reduction in Ca**												
1	randomised trials	serious[a]	not serious	not serious	serious[b]	none	4.6	1	-	MD **3.7 % lower** (8.6 lower to 1.3 higher)	⨁⨁◯◯ Low	CRITICAL
**Elevated percentage of P**												

Certainty assessment							№ of patients		Effect		Certainty	Importance
№ of studies	Study design	Risk of bias	Inconsistency	Indirectness	Imprecision	Other considerations	Cinacalcet	Placebo	Relative (95% CI)	Absolute (95% CI)		
1	randomised trials	serious[a]	not serious	not serious	serious[b]	none	2.9	9.3	-	MD **6.4 % lower** (21 lower to 8.2 higher)	⊕⊕◯◯ Low	CRITICAL
**Growth in length**												
1	randomised trials	serious[a]	not serious	not serious	serious[b]	none	3.3	3.1	-	**0** (3.1 lower to 3.6 higher)	⊕⊕◯◯ Low	CRITICAL

CI: confidence interval; **MD:** mean difference; **OR:** odds ratio

Explanations

a. This study was funded by Amgen Inc, which is the manufacturer of cinacalcet.
b. The sample size was small.

Note：The evidence summarized in table S45-S49 directly supports Recommendation 15.1 and Recommendation 15.2 (see Page 67).

Appendix C. Work Group membership

WORK GROUP CO-CHAIRS

Aihua Zhang, MD
Children's Hospital of Nanjing Medical University
Nanjing, China

Qiu Li, MD
Children's Hospital of Chongqing Medical University
Chongqing, China

Work group and disclosure information

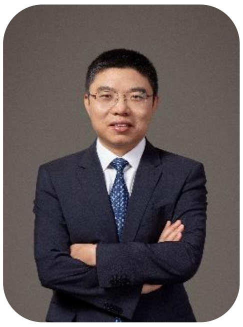

Ai-Hua Zhang, M.D., Ph.D., is a professor and the dean of the School of Pediatrics, Nanjing Medical University; the president of the Chinese Society of Pediatric Nephrology. He is also the President of Nanjing Children's Hospital. Prof. Zhang's research interests are the pathogenic mechanisms of acute or chronic kidney diseases and congenital rare diseases with renal phenotypes. His research works have been published on over 180 articles in prestigious journals such as *Sci Transl Med*, *Adv Sci*, *Nat Comm*, *PNAS*, *Kidney Int*, *JASN*, *JBC*, etc.

Dr. Ai-hua Zhang declared no competing interests.

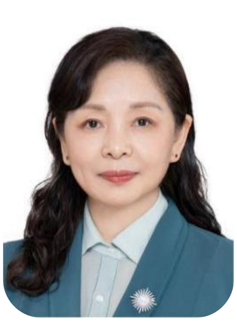

Qiu Li, M.D., Ph.D., is a professor, doctoral supervisor, director of the National Clinical Research Center for Children and Adolescents' Health and Diseases, an expert with outstanding contributions from young and middle-aged people of the National Health Commission, Standing Committee Member and Secretary-General of the Pediatric Branch of the Chinese Medical Association, deputy director of Nephrology Group of Pediatric Branch of Chinese Medical Association, deputy director of Children's Hospital Branch of Chinese Hospital Association, deputy director of Pediatrics Professional Steering Committee of University Education Guidance Committee of Ministry of Education, standing member of Pediatric Branch of Chinese Medical Doctor Association, deputy director of Adolescent Health Branch of Chinese Medical Doctor Association. She has won the Second Prize of Science and Technology Progress of the Ministry of Education, the First Prize of Chongqing Science and Technology Progress, and the First Prize of Chongqing Teaching Achievement. She has presided over and guided more than 10 national and provincial projects, more than 20 provincial and ministerial projects, and published more than 160 papers.

Dr. Qiu Li declared no competing interests.

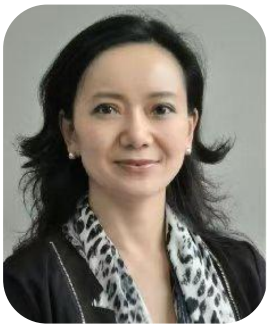

Mo Wang, M.D., Ph.D., is a professor and the executive director of Yibin Hospital Affiliated to Children's Hospital of Chongqing Medical University. She also serves as the Director of the Renal Special Disease Diagnosis and Treatment Center in Chongqing and the Deputy Director of the key Laboratory of Children's Vital Organ Development and Diseases of the Chongqing Health Commission. She has held prominent leadership roles, including Deputy Leader of the Rare Diseases Group of the Pediatric Branch of the Chinese Medical Association, Member and Secretary-General of the Nephrology Group of the Pediatric Branch of the Chinese Medical Doctors Association, Member of the Nephrology Committee of the Chinese Physiology Association, and the Chairperson of the ANCA-related Vasculitis Special Committee of the China Rare Disease Alliance. Her research primarily focuses on the genetics and immunopathogenesis of pediatric kidney diseases, as well as integrated management of uremia. She has led key research project funded by the Ministry of Science and Technology, National Natural Science Foundation of China, and over 10 provincial and ministerial projects. Her honors include the First Prize of Chongqing Science and Technology Progress, the Second Prize of Science and Technology Progress form the Ministry of Education, and the Song Qingling Pediatric Medicine Award. She has authored over 80 academic papers and serves as a Guest Editor of Frontiers journals, the Executive Editor of *Pediatric Pharmacy*, and a member of the editorial board of *Chinese Journal of General Practice*, and the *Journal of Clinical Nephrology*.

Dr. Mo Wang declared no competing interests.

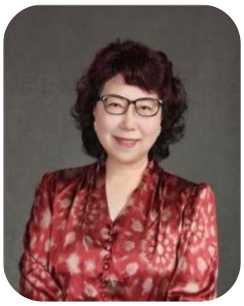

Hong Xu, M.D., Ph.D., is the chief physician. She graduated from Shanghai Medical College of Fudan University and her research field is pediatrics. Articles have been published in Pediatric Nephrology and other journals. She also served on the editorial board of the *Chinese Journal of Pediatrics*.

Dr.Hong Xu declared no competing interests.

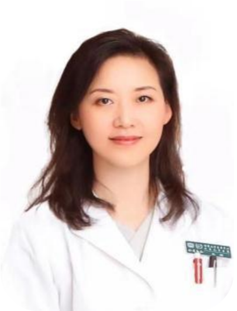

Hui Wang, MD, is the member of Chinese Medicine and Integrated Chinese and Western Medicine Branch of China Maternal and Child Health Association, member of Nephrology Branch of Beijing Medical Association, member of Nephrology Group, Rare Diseases Branch, Beijing Medical Association, member of Department of Nephrology, Capital Medical University, Xinjiang Tianchi talents special experts. Her main research area is pediatric kidney diseases.

Dr. Hui Wang declared no competing interests.

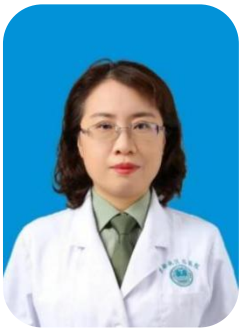

Chun-lin Gao, M.D., Ph.D., is the member of the Nephrology Group of the Pediatrics Branch of the Chinese Medical Association, deputy Chairman of the Pediatrics Branch of the Jiangsu Medical Association, deputy leader of the Pediatric nephrology Group of the Pediatrics Society of the Jiangsu Medical Association, member of the Pediatrics branch of the Jiangsu Medical Association, member of the Pediatric Intensive care group of the Jiangsu Medical Association. She studied at the Department of Pediatrics, Shanxi Medical University from 1994 to 1999, pursued a master's degree in Pediatrics, SuZhou University from 1999 to 2002, worked at the Department of Pediatrics, Jinling Hospital from 2002 to present, and pursued a doctorate in pediatrics, Nanjing Medical University from 2007 to 2010. Her main research area is pediatric kidney diseases. She has published 56 papers, including 26 SCI papers. She also served as the editorial board member of the *Journal of Clinical Pediatrics*, *Pharmacy and Clinical Research*, *Parenteral and Enteral Nutrition*, *Medical Research and Trauma Treatment*, and the corresponding editorial board member of the 6th editorial Board of the *Chinese Journal of Contemporary Pediatrics*.

Dr. Chun-lin Gao declared no competing interests.

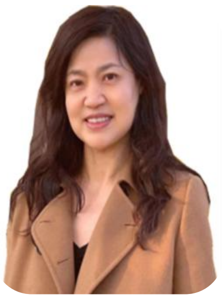

Xiao-rong Liu, MD, is the deputy leader of Chinese Medical Doctor Association children's blood purification committee. She graduated from Capital Medical University and worked in Beijing Children's Hospital. Her research area is chronic kidney disease. She has published over 110 papers. She is also a member of the editorial board of the *Chinese Journal of Practical Pediatrics*.

Dr. Xiao-rong Liu declared no competing interests.

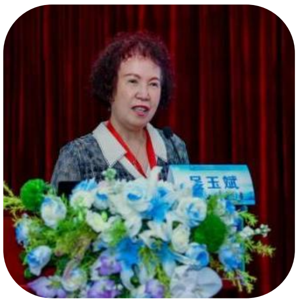

Yu-bin Wu, M.D., Ph.D., is the member of the 15th-17th session of Chinese Medical Nephrology Group, the Chairman of the third Special Committee of Children's Blood Purification, Pediatrician Branch of Chinese Medical Doctor Association. She has been working in the Department of Pediatrics of Shengjing Hospital since 1988. Her main research area is pediatric kidney diseases. She has published more than 100 papers. She is also the editorial board member and special editorial board member of *Chinese Journal of Practical Pediatrics*, the editorial board member of *Chinese Journal of Practical Pediatrics*, the editorial board member of *Chinese Journal of Pediatric First Aid*, and the deputy editor of *Chinese Journal of Integrated Traditional and Western Medicine Pediatrics*.

Dr. Yu-bin Wu declared no competing interests.

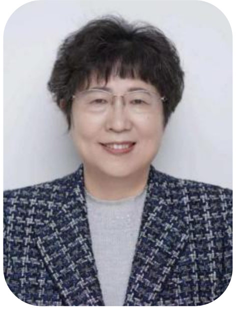

Shu-Zhen Sun, M.D., Ph.D., is the member of 15th-17th session of the nephrology Group of the Pediatric Branch of the Chinese Medical Association, member of the Standing Committee of the Kidney Professional Committee of the Pediatric branch of the Chinese Medical Doctor Association and member of the Blood purification Group, member of the National Pediatric Education Advisory Committee of the Ministry of Education, expert member

of the pediatric subprofessional group of the National Kidney Disease Professional Medical Quality Control Center, deputy director of the pediatric branch of the Shandong Medical Association and leader of the renal Immunology Group, and Shandong Physicians Association Standing Committee member of Pediatrics Branch and Chairman of renal specialty. She studied in the Medical Department of Shandong Medical University and obtained a bachelor's degree (1983-1988). She studied at Shandong University and received a master's degree (1998 - 2001) and a doctoral degree (2003 - 2006). She has been working in the pediatrics department of Shandong Provincial Hospital, Shandong Medical University since 1988. Her research focuses on pediatric renal rheumatism and rare diseases. She has published 12 SCI papers. She also served as a guest reviewer of the *Chinese Clinical Journal of Practical Pediatrics* and a guest editorial member of the *International Journal of Pediatrics*.

Dr. Shu-zhen Sun declared no competing interests.

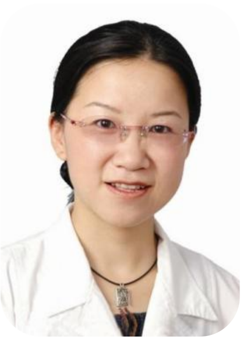

Xiao-shan Shao, M.D., Ph.D., is the member of nephrology Group of Pediatrics Branch of Chinese Medical Association, member of Pediatrics Branch of Chinese Medical Doctor Association, member of nephrology Group of Pediatrics Branch of Chinese Medical Doctor Association, member of nephrology Group of Children's Diseases and Health Branch of Chinese Maternal and Child Health Society, member of Asia-Pacific Society of Medical Biological Immunology. From 2006 to 2009, she received her doctorate degree from Chongqing Medical University. She participated in the International Pediatric Kidney Training Class in Fudan Pediatric Hospital and Princess Margaret Hospital of Hong Kong and obtained the fellow qualification certificate (2010-2011). She was a senior visiting scholar at the Children's Hospital Affiliated to University of California, San Diego (2015-2016). Her research interests are renal rheumatic immunity diseases and blood purification in children. She has published 8 SCI papers. She served as the director of the Pediatric renal Rheumatology Department of Guiyang Maternal and Child Health Hospital (2005-2012). She served as the vice president and member of the Party Committee of Guiyang Maternal and Child Health Hospital (2012-2020). She served as the president and deputy secretary of the Party Committee of Guiyang Maternal and Child Health Hospital (2020 to now).

Dr. Xiao-shan Shao declared no competing interests.

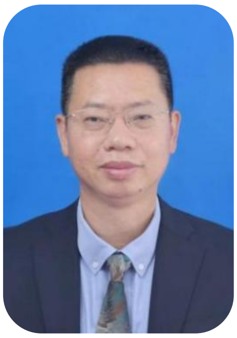

Yu-hong Tao, M.D., Ph.D., is the vice Chairman of Blood Purification Professional Committee of Pediatricians Branch of Chinese Medical Doctor Association, leader of Nephrology Group of Pediatrics Professional Committee of Sichuan Medical Association, Standing Committee of Nephrology Professional Committee of Sichuan Medical Association, Chairman of Pediatric Enuresis Disease Management Professional Committee of Sichuan Medical Communication Society, member of Pediatrics Professional Committee of Sichuan Medical Doctor Association, Member of nephrology Expert Committee of Pediatricians Branch of Chinese Medical Doctor Association, member of Nephrology Physician Branch of Sichuan Medical Doctor Association, Standing Committee of Nephrology Committee of Sichuan Association of Integrative Chinese and Western Medicine, Secretary of Nephrology Group of Pediatrics Branch of Chinese Medical Association, etc. He received his Master's degree in Medicine in 2001 and his doctor's degree in 2007. He was a visiting scholar in the nephrology Department at Johns Hopkins University School of Medicine from 2010 to 2011. He has been working in the pediatrics department of West China Second Hospital of Sichuan University since 1991. His research focuses on pediatric kidney diseases. He has published 4 SCI papers. He is also an expert reviewer of the *Chinese Journal of Pediatrics* and an editorial member of the *Chinese Journal of Contemporary Pediatrics*.

Dr. Yu-hong Tao declared no competing interests.

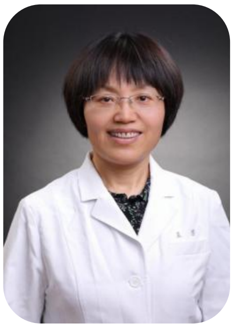

Fang Wang, M.D., Ph.D., is the deputy leader of the 18th Nephrology Group of the Pediatrics Branch of the Chinese Medical Association, deputy leader of the Pediatric Nephrology Group of the third Committee of the Rare Disease Branch of the Beijing Medical Association, standing member of the Pediatric

Rheumatology and Immunology Committee of the Beijing Society of Integrative Medicine, and standing member and secretary-general of the first Rare disease Professional Committee of the Chinese Women Medical Association. Since 2003, she has been working in Peking University First Hospital. Her research focuses on pediatric kidney diseases. As the (co-) first author or corresponding author, she has published nearly 70 articles at home and abroad, including the authoritative academic journal *Kidney International*. She also serves on the editorial board of the *Chinese Journal of Pediatrics*, the *Chinese Journal of Contemporary Pediatrics*, and serves as a reviewer of Kidney *International*, *American Journal of Kidney Disease*, *Clinical Genetics*, *BMC Nephrology*, *Nephrology* and other journals.

Dr. Fang Wang declared no competing interests.

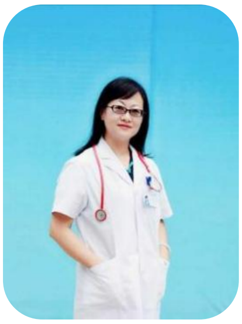

Li-Jun Zhao, M.M., is the member of nephrology Group of the 18th Committee of Pediatrics Branch of Chinese Medical Doctor Association, member of Pediatric Kidney Disease Group of the 4th Committee of Pediatricians Branch of Chinese Medical Doctor Association, member of Children's Blood Purification Group of Pediatricians Branch of Chinese Medical Doctor Association, Member of Kidney Disease and Health Science Group, Children's Diseases and Health Care Branch of China Maternal and Child Health Care Association, Member of Collaborative Innovation Community Committee of Children's Purpura and kidney disease of Chinese Society of Traditional Chinese Medicine, member of the first Professional Committee of China Rare Disease Alliance/Beijing Rare Disease Diagnosis, Treatment and Protection Society of Atypical Hemolytic uremic Syndrome in Children, Member of the Medical Quality Control Center of Renal Department of Shanxi Provincial Health Commission, Director of Shanxi Provincial Pediatric Medical Quality Control Center. Since 1998, she has been working in Shanxi Children's Hospital. In 2012, she participated in the International Pediatric Nephrology Society (IPNA) training at the Renal Center of the Children and Adolescents Department of Princess Margaret Hospital, Hong Kong. Her research interests are pediatric kidney diseases and blood purification. She has published 4 papers. She also served on the editorial board of *Anhui Medical Journal*.

Dr. Li-jun Zhao declared no competing interests.

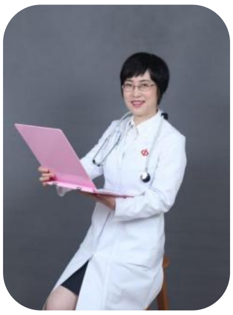

Xiao-yun Jiang, M.D., Ph.D., is the deputy head of the General Medicine Group, Pediatrics Branch of Chinese Medical Association, member of the Pediatrics Branch of Chinese Medical Doctor Association, and chair-designate of the Pediatrics Branch of Guangdong Medical Association, the chairman of the special Committee of Pediatrics and Adolescent Health Management of Guangdong Provincial Society of Health Management, and a member of the teaching Steering Committee of Clinical Teaching Base of Undergraduate universities in Guangdong Province. She received her Ph.D. from Sun Yat-sen University in 2002. She is currently the director of the Department of Pediatrics, the director of the Teaching and Research section, and the director of the pediatric training Base of the First Affiliated Hospital of Sun Yat-sen University. She was a senior Visiting Scholar at Monash University in Australia in 2018 and a senior visiting scholar at Children's Hospital of Philadelphia in the United States in 2013. She has published 5 SCI papers. She was responsible for and participated in the formulation and updating of six evidence-based Guidelines for the Diagnosis and Treatment of Common Kidney Diseases in Children, and participated in 25 guidelines or expert consensus.

Dr. Xiao-yun Jiang declared no competing interests.

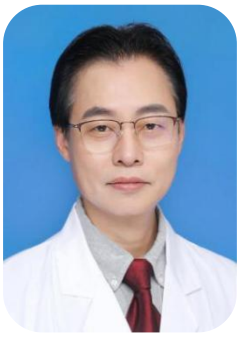

Ying-jie Li, B.M., is the member of the 16th, 17th and 18th Pediatric nephrology Group of Chinese Medical Association. Since July 1985, he has been working in the internal medicine department of Guangzhou Women and Children Medical Center (formerly Guangzhou Children's Hospital). His research area is pediatric kidney. He has published 4 SCI papers. He also served as an expert reviewer of Hubei Chen Xiaoping Science and Technology Development Foundation.

Dr. Ying-jie Li declared no competing interests.

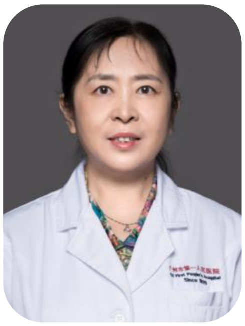

Li Yu, M.M., is the member of the nephrology Group of the Pediatric Branch of the Chinese Medical Association, deputy chairman of the pediatric special Committee of the Chinese Women Medical Association, Standing Committee of the pediatric kidney special committee of the Chinese Maternal and Child Health Association, member of the pediatric special committee of the Chinese research Hospital, deputy chairman of the pediatric branch of the Guangdong Medical Association. From 1983 to 1992, she worked in the pediatrics department of the Affiliated Hospital of Chengde Medical College, Hebei Province. She received her master's degree in 1995. Since 1996, she has been working in the pediatrics department of Guangzhou First People's Hospital. Her research interests are renal and immune diseases in children. She has published 6 papers. She is also a member of the editorial board of the *Chinese Clinical Journal of Practical Pediatrics*, the *Journal of Clinical Pediatrics*, the *Journal of Guangdong Medicine* and the *Journal of Practical Medicine*.

Dr. Li Yu declared no competing interests.

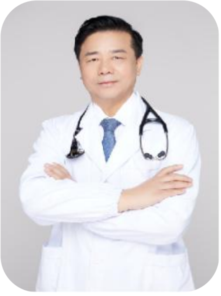

Zheng-kun Xia, M.D., Ph.D., is the member of the Pediatrics Branch of the Chinese Medical Association and deputy head of the nephrology Group, member of the Pediatrics Branch of the Chinese Medical Association, deputy head of the nephrology Group of the 15th and 16th Pediatrics Branch of the Chinese Medical Association, Chairman of the Pediatrics Branch of the 10th Jiangsu Medical Association and the head of the children's Nephrology Group, vice president of the Pediatrics branch of the Jiangsu Medical Association, deputy chairman of the army pediatric Professional committee and the child kidney Group leader. He received his doctorate from the Second Military Medical University in 2007. He was a visiting scholar at Sydney Children's Hospital, University of New South Wales, Australia in 2012 and 2014 respectively. Since 1994, he has worked in the General Hospital of the Eastern Theater Command. His research interests include pediatric refractory kidney disease, IgA nephropathy, purpura nephritis, lupus nephritis, unexplained hematuria proteinuria, blood purification, critical illness, vasculitis, and nocturnal enuresis. He has published 10 SCI papers. He also serves on the editorial board of the *Chinese Journal of Pediatrics*, the *Journal of Graduate Medical Journal*, the *Journal of Clinical Pediatrics*, the *International Journal of Pediatrics* and the *Journal of Modern Medicine*.

Dr. Zheng-kun Xia declared no competing interests.

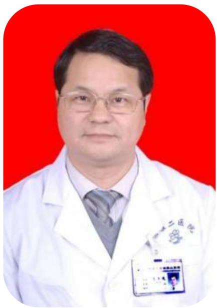

Xi-qiang Dang，M.D., Ph.D.， is the member of the nephrology Group of the Pediatric Branch of the Chinese Medical Association, member of the pediatric kidney Disease Group of the Pediatrician Branch of the Chinese Medical Doctor Association, standing member of the Pediatric Professional Committee of the Chinese Society of Research Hospitals, vice Chairman of the Collaborative Innovation Community Committee of the Chinese Society of Traditional Chinese Medicine in Children with Purpura Nephropathy, etc. He Graduated in July 1986, assigned to the Second Xiangya Hospital (formerly the Second Affiliated Hospital of Hunan Medical University), has been engaged in pediatric clinical, teaching, scientific research work. He received a Master's degree in medicine in 1999. He was promoted to deputy chief physician and associate professor in 2000 and was promoted to chief physician and professor in 2012. From 2013 to 2014, he studied at Children's Hospital Los Angeles. From 2006 to 2012, he served as deputy director of the Clinical Center of Pediatric Kidney Disease in Hunan Province; from 2012 to 2015, he served as director of the Clinical Center of Pediatric Kidney Disease; from 2011 to 2021, he served as the first person in charge of the clinical trial base of pediatric kidney drugs. In 2012, he was the first director of the pediatric kidney Department of the Children's Medical Center of Xiangya Second Hospital. His research interests include pediatric kidney and rheumatic immunity diseases. He has published 6 papers. He also serves as the editorial board member of the *International Journal of Pediatrics*, the special editorial board member of the *Chinese Journal of Practical Pediatrics* and the corresponding editorial board member of the *Chinese Journal of Contemporary Pediatrics*.

Dr. Xi-qiang Dang declared no competing interests.

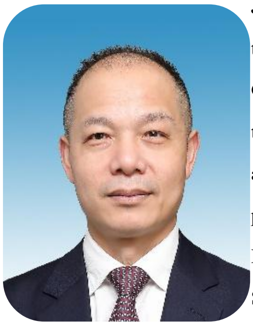

Jian-hua Mao, M.D., Ph.D., is the consultant of the nephrology Group of the Pediatric Branch of the Chinese Medical Association and the deputy director of the pediatric Branch of the Zhejiang Medical Association. From September 1993 to August 1996, he studied for a master's degree in Kunming Medical University. From September 2002 to June 2005, he studied for his doctorate at Zhejiang University. His research interests are the immunopathogenesis and genetic background of primary nephrotic syndrome in children. He has published many high-quality papers. He also serves as a member of the editorial boards of several journals, including *Acta Physiol (Oxf)*, *Pediatric Nephrology*, *World Journal of Pediatrics*, *Chinese Journal of Pediatrics*, *Chinese Journal of Nephrology*, *Chinese Journal of Evidence-based Pediatrics*, *Chinese Journal of Contemporary Pediatrics*, etc.

Dr. Jian-hua Mao declared no competing interests.

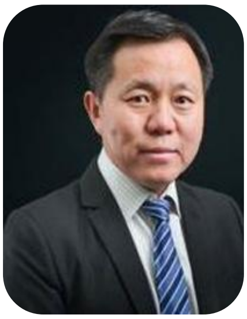

Jian-hua Zhou, M.D., Ph.D., is the director of pediatric nephrology，Tongji Hospital, Tongji Medical College, Huazhong University of Science & Technology. He obtained a bachelor's degree in Medicine (1980/9-1985/7) from Tongji Medical University, and a master's degree in Pediatrics (1987/9-1990/7), and a Ph.D. degree in Medicine (1999/5-2001/12). He served as an Attending Physician in Department of Pediatrics, Tongji Hospital, Tongji Medical University (1990/7-1998/9), and was promoted as an Associate Professor (1998/10-2004/9). He has been working as a Professor/Chief Physician/Director of Pediatric Nephrology in Tongji Hospital since 2004/10. He is the Vice Chairman of Translational Medicine Committee, Chinese society of Pediatrics; Member, Pediatrics Branch, Chinese Physicians Association; Vice Chairman, Renal Disease Expert Committee, Chinese Pediatrician Association; Vice Chairman, Blood Purification Experts Committee, Chinese Pediatrician Association; Vice Chairman, Pediatrics Branch, Hubei Provincial Pediatrician Association etc. He is the Editorial Board Member of 9 Journals: *Chinese Journal of Pediatrics*; *Chinese Clinical Journal of Practical Pediatrics*; *Chinese Journal of Evidence-Based Pediatrics*; *Chinese Journal of Practical Pediatrics*; *Journal of Clinical Pediatrics*; *Journal of Clinical Nephrology*, and etc. He has published 32 SCI papers.

Dr. Jian-hua Zhou declared no competing interests.

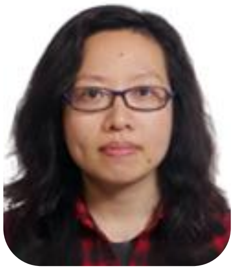

Qian Shen, M.D., Ph.D., obtained her Bachelor's degree in Clinical Medicine from Shanghai Medical University (1993-1998), followed by a Master's degree (2001-2004) and a Ph.D. (2005-2010) in Pediatric Nephrology from Shanghai Medical College of Fudan University. She established China's first and largest pediatric dialysis and renal transplantation center for uremia, achieving survival rates on par with international standards. Her groundbreaking research spans the discovery of novel pathogenic genes to multidimensional analyses (genetic, epigenetic, and environmental-genetic interactions) of hereditary kidney diseases—the leading cause of pediatric uremia—providing pivotal insights for clinical decision-making. Currently, she holds leadership roles including Vice Chairperson of the Nephrology Group, Pediatric Branch, Chinese Medical Association; Member of the Renal Physiology Committee, Chinese Society of Physiology; Member and Secretary-General of the Pediatric Branch, Shanghai Medical Association, and Chairperson of its Nephrology Group, etc. She is leading four National Natural Science Foundation of China projects, has published 40 SCI papers as first or corresponding author, and contributed to the international authoritative textbook Pediatric Nephrology (8th Edition, 2022) and two international guidelines (Peritoneal Dialysis International 2024 and Pediatric Nephrology 2024). Additionally, she serves as an Editorial Board Member for the *Journal of Clinical Pediatrics* and *BMC Pediatrics*, and as a Reviewer for journals such as *Chinese Journal of Nephrology*, *Chinese Journal of Evidence-Based Pediatrics*, *Rare Disease Research*, *Kidney International*, *Kidney Diseases*, *Pediatric Nephrology*, and *Chinese Medical Journal*.

Dr. Qian Shen declared no competing interests.

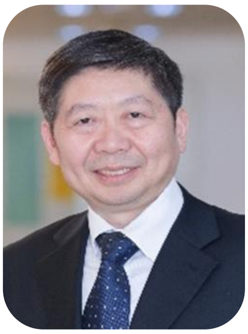

Wen-yan Huang, M.D., Ph.D., is the member of the Nephrology Group of the Pediatrics Branch of the Chinese Medical Association, member of the Pediatric Blood Purification Professional Committee of the Chinese Medical Doctor Association, member of the Pediatrics Professional Committee of the Second Chinese Society of Research Hospitals, Member of the 12th Committee of the Nephrology Specialized Branch of the Shanghai Medical Association, member of the 7th Nephrology Specialized Committee of the Shanghai Branch of Integrated Traditional Chinese and Western Medicine, member of the Nephrology Group of the Shanghai Medical Doctor Association. He graduated from the Department of Pediatric Nephrology of Nanjing Medical University in July 1997, obtaining a Master of Medicine degree. He graduated from the Department of Pediatric Nephrology of Nanjing Medical University in July 2003, obtaining a Doctor of Medicine degree. He graduated from the Department of Pediatric Nephrology of Georgetown University in the United States in August 2006, obtaining a postdoctoral degree. He has published 9 SCI papers and serves as an editorial board member of the *Chinese Journal of Pediatrics*, the *Journal of Clinical Pediatrics*, the *International Journal of Pediatrics*, and the *Chinese Journal of Pediatric Emergency Care*.

Dr. Wen-yan Huang declared no competing interests.

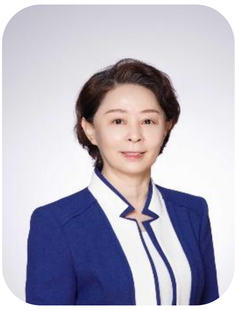

Ying Shen, B.M., Professor, obtained her Bachelor's degree from Beijing Second Medical College (now Capital Medical University) (1978-1982), and later pursued advanced training in pediatric nephrology at Regina Margherita Children’s Hospital in Turin, Italy (1995-1997). Currently, she holds prominent academic roles, including Chairperson of the First Blood Purification Committee and Vice Chairperson of the First Pediatric Nephrology Committee under the Chinese Medical Doctor Association’s Pediatrician Branch, Chairperson of the 13th Pediatrics Committee of the Beijing Medical Association, Vice Chairperson of its Rare Diseases Committee, and Vice President of the China Healthy Birth Science Association. With a career dedicated to pediatric blood purification and kidney disease research, she pioneered pediatric hemodialysis in China, authored the nation’s first authoritative textbook on pediatric blood purification, and spearheaded the nationwide adoption of pediatric blood purification techniques. She has led over 20 national and provincial/ministerial-level research projects, published more than 170 academic papers (including more than 60 SCI-indexed articles), and authored 23 monographs. Additionally, she serves on the editorial boards of leading medical journals such as *Chinese Journal of Pediatrics*, *Chinese Pediatric Emergency Medicine*, *Chinese Journal of Integrated Traditional and Western Medicine*, *Chinese Journal of Applied Clinical Pediatrics*, and *Beijing Medical Journal*.

Dr. Ying Shen declared no competing interests.

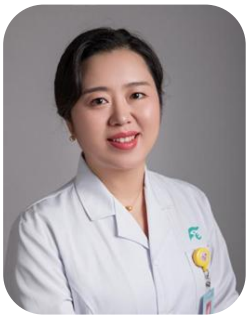

Xiao-wen Wang, M.D., Ph.D., obtained a master's degree from Hubei University of Chinese Medicine in June 2000 and a doctoral degree in pediatrics from Fudan University in June 2015. Her research field is pediatric kidney diseases, including the pathogenesis of congenital renal urinary tract malformations, mammalian kidney development, hereditary kidney diseases, primary and secondary glomerular diseases, and replacement therapy for CKD. She is the member of the Nephrology Youth Group of the Pediatrics Branch of the Chinese Medical Association, blood Purification Committee of the Pediatrics Branch of the Chinese Medical Doctor Association, member of the Pediatrics Expert Committee on Capacity Building and Continuing Education of the National Health Commission, member of the Collaborative Innovation Community Committee for Children's Purpura Nephropathy of the China Association of Chinese Medicine, member of the Nephrology Branch of Hubei Medical Association and Director of the Hubei Pediatric Quality Control Center, etc. She has published five SCI papers and serves as an editorial board member of the *Journal of Clinical Nephrology* and a reviewer of the *Journal of Pediatric Nephrology*.

Dr. Xiao-wen Wang declared no competing interests.

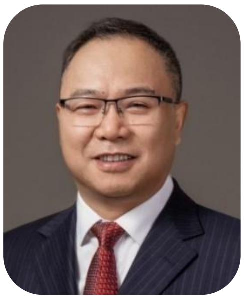

Song-ming Huang, M.D., Ph.D., is the stand member of Chinese Pediatric Society, Chinese Medical Association. He graduated from Nanjing Medical University and work in Nanjing Children's Hospital, Affiliated Nanjing Medical University. His research interests are kidney diseases and renal pathology. His research papers were published in *AJP renal*, *Pediatr Nephol* and other journals. He is also the member of editorial board of the *Chinese Journal of Practical Pediatrics* and *Word Journal of Pediatrics*.

Dr. Song-ming Huang declared no competing interests.

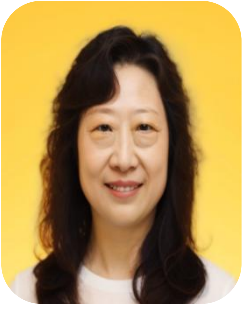

Dong-feng Zhang, M.M., is the member of Nephrology Group, Pediatrics Society of Chinese Medical Association; Member of Pediatric Nephrology Expert Committee, Pediatricians Branch, Chinese Medical Doctor Association; Committee member of Pediatric Rheumatism and Immunology, Pediatrician Branch of Chinese Medical Doctor Association, Executive Director of Hebei Pediatric Society; Chairman of the Special Committee on Childhood Kidney and Rheumatism; Chairman of Pediatric Kidney and Rheumatism Immunity Professional Committee of Hebei Association of Integrated Traditional Chinese and Western Medicine; Director of Hebei Association of Integrative Chinese and Western Medicine; Member of Pediatrics Professional Committee of Hebei Association of Integrated Chinese and Western Medicine; Member of the Standing Committee of Nephrology Branch of Hebei Medical Association; Hebei Provincial Hospital Association blood purification center management committee member. She has been working in Hebei Children's Hospital since 1986. Her research interests are pediatric kidney and rheumatic immunity diseases. She has published 5 papers.

Dr. Dong-feng Zhang declared no competing interests.

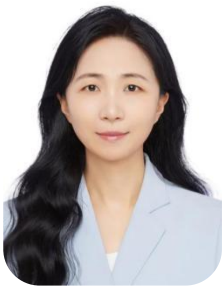

Hui-mei Huang, M.M., is the member of Nephrology Group, Pediatrics Society of Chinese Medical Association; Member of Pediatric Nephrology Group and Youth Group, Pediatricians Branch, Chinese Medical Doctor Association; Member of Pediatric Committee of Chinese Association of Integrated Traditional Chinese and Western Medicine, Renal Rheumatism and Immunology Group, Member of Child Health and Drug Research Committee of Chinese Association of Traditional Chinese Medicine; Member of the National Professional Group of Integrative Pediatrics, Chinese Integrative Medicine Expert Volunteer Committee; Member of Adolescent Health and Medical Professional Committee of Shaanxi Provincial Doctors Association; Member of Shaanxi Region Child Enuresis disease Management Cooperation Group; Member of Children's Disease Prevention and Treatment Committee of Shaanxi Health Association; Member and secretary of Pediatrics Branch of Xi 'an Medical Association. She graduated from Xi'an Jiaotong University School of Medicine, and has been working in the nephrology Department of Xi'an Children's Hospital since 2005. From March to June 2011, she studied blood purification in the Blood Purification Center of Beijing Children's Hospital Affiliated to Capital Medical University. Her research interests are kidney diseases in children. She has published 11 papers.

Dr. Hui-mei Huang declared no competing interests.
